# Human Health and Ocean Pollution

**DOI:** 10.5334/aogh.2831

**Published:** 2020-12-03

**Authors:** Philip J. Landrigan, John J. Stegeman, Lora E. Fleming, Denis Allemand, Donald M. Anderson, Lorraine C. Backer, Françoise Brucker-Davis, Nicolas Chevalier, Lilian Corra, Dorota Czerucka, Marie-Yasmine Dechraoui Bottein, Barbara Demeneix, Michael Depledge, Dimitri D. Deheyn, Charles J. Dorman, Patrick Fénichel, Samantha Fisher, Françoise Gaill, François Galgani, William H. Gaze, Laura Giuliano, Philippe Grandjean, Mark E. Hahn, Amro Hamdoun, Philipp Hess, Bret Judson, Amalia Laborde, Jacqueline McGlade, Jenna Mu, Adetoun Mustapha, Maria Neira, Rachel T. Noble, Maria Luiza Pedrotti, Christopher Reddy, Joacim Rocklöv, Ursula M. Scharler, Hariharan Shanmugam, Gabriella Taghian, Jeroen A.J.M. van de Water, Luigi Vezzulli, Pál Weihe, Ariana Zeka, Hervé Raps, Patrick Rampal

**Affiliations:** 1 Boston College, US; 2 Woods Hole Center for Oceans and Human Health, Woods Hole Oceanographic Institution, US; 3 European Centre for Environment and Human Health, GB; 4 University of Exeter Medical School, GB; 5 Centre Scientifique de Monaco, MC; 6 Centers for Disease Control and Prevention, US; 7 Université Côte d’Azur, FR; 8 Centre Hospitalier Universitaire de Nice, Inserm, C3M, FR; 9 International Society of Doctors for the Environment (ISDE), CH; 10 Health and Environment of the Global Alliance on Health and Pollution (GAHP), AR; 11 Intergovernmental Oceanographic Commission of UNESCO, FR; 12 IOC Science and Communication Centre on Harmful Algae, University of Copenhagen, DK; 13 Ecotoxicologie et développement durable expertise ECODD, Valbonne, FR; 14 Centre National de la Recherche Scientifique, FR; 15 Muséum National d’Histoire Naturelle, Paris, FR; 16 Scripps Institution of Oceanography, University of California San Diego, US; 17 Trinity College Dublin, IE; 18 Institut Français de Recherche pour l’Exploitation des Mers, FR; 19 University of Exeter, GB; 20 CIESM The Mediterranean Science Commission, MC; 21 Harvard University T.H. Chan School of Public Health, US; 22 University of California at San Diego, US; 23 Universidad de la República, UY; 24 Institute for Global Prosperity, University College London, GB; 25 Strathmore University Business School, Nairobi, KE; 26 Nigerian Institute for Medical Research, Lagos, NG; 27 Imperial College London, GB; 28 World Health Organization, CH; 29 University of North Carolina at Chapel Hill, US; 30 Sorbonne Université, FR; 31 Department of Marine Chemistry and Geochemistry, Woods Hole Oceanographic Institution, US; 32 Department of Public Health and Clinical Medicine, Section of Sustainable Health, Umeå University, Umeå, SE; 33 University of KwaZulu-Natal, ZA; 34 University of Genoa, IT; 35 University of the Faroe Islands and Department of Occupational Medicine and Public Health, FO; 36 Brunel University London, GB; 37 WHO Collaborating Centre for Health and Sustainable Development, MC

## Abstract

**Background:**

Pollution – unwanted waste released to air, water, and land by human activity – is the largest environmental cause of disease in the world today. It is responsible for an estimated nine million premature deaths per year, enormous economic losses, erosion of human capital, and degradation of ecosystems. Ocean pollution is an important, but insufficiently recognized and inadequately controlled component of global pollution. It poses serious threats to human health and well-being. The nature and magnitude of these impacts are only beginning to be understood.

**Goals:**

(1) Broadly examine the known and potential impacts of ocean pollution on human health. (2) Inform policy makers, government leaders, international organizations, civil society, and the global public of these threats. (3) Propose priorities for interventions to control and prevent pollution of the seas and safeguard human health.

**Methods:**

Topic-focused reviews that examine the effects of ocean pollution on human health, identify gaps in knowledge, project future trends, and offer evidence-based guidance for effective intervention.

**Environmental Findings:**

Pollution of the oceans is widespread, worsening, and in most countries poorly controlled. It is a complex mixture of toxic metals, plastics, manufactured chemicals, petroleum, urban and industrial wastes, pesticides, fertilizers, pharmaceutical chemicals, agricultural runoff, and sewage. More than 80% arises from land-based sources. It reaches the oceans through rivers, runoff, atmospheric deposition and direct discharges. It is often heaviest near the coasts and most highly concentrated along the coasts of low- and middle-income countries. Plastic is a rapidly increasing and highly visible component of ocean pollution, and an estimated 10 million metric tons of plastic waste enter the seas each year. Mercury is the metal pollutant of greatest concern in the oceans; it is released from two main sources – coal combustion and small-scale gold mining. Global spread of industrialized agriculture with increasing use of chemical fertilizer leads to extension of Harmful Algal Blooms (HABs) to previously unaffected regions. Chemical pollutants are ubiquitous and contaminate seas and marine organisms from the high Arctic to the abyssal depths.

**Ecosystem Findings:**

Ocean pollution has multiple negative impacts on marine ecosystems, and these impacts are exacerbated by global climate change. Petroleum-based pollutants reduce photosynthesis in marine microorganisms that generate oxygen. Increasing absorption of carbon dioxide into the seas causes ocean acidification, which destroys coral reefs, impairs shellfish development, dissolves calcium-containing microorganisms at the base of the marine food web, and increases the toxicity of some pollutants. Plastic pollution threatens marine mammals, fish, and seabirds and accumulates in large mid-ocean gyres. It breaks down into microplastic and nanoplastic particles containing multiple manufactured chemicals that can enter the tissues of marine organisms, including species consumed by humans. Industrial releases, runoff, and sewage increase frequency and severity of HABs, bacterial pollution, and anti-microbial resistance. Pollution and sea surface warming are triggering poleward migration of dangerous pathogens such as the *Vibrio* species. Industrial discharges, pharmaceutical wastes, pesticides, and sewage contribute to global declines in fish stocks.

**Human Health Findings:**

Methylmercury and PCBs are the ocean pollutants whose human health effects are best understood. Exposures of infants *in utero* to these pollutants through maternal consumption of contaminated seafood can damage developing brains, reduce IQ and increase children’s risks for autism, ADHD and learning disorders. Adult exposures to methylmercury increase risks for cardiovascular disease and dementia. Manufactured chemicals – phthalates, bisphenol A, flame retardants, and perfluorinated chemicals, many of them released into the seas from plastic waste – can disrupt endocrine signaling, reduce male fertility, damage the nervous system, and increase risk of cancer. HABs produce potent toxins that accumulate in fish and shellfish. When ingested, these toxins can cause severe neurological impairment and rapid death. HAB toxins can also become airborne and cause respiratory disease. Pathogenic marine bacteria cause gastrointestinal diseases and deep wound infections. With climate change and increasing pollution, risk is high that *Vibrio* infections, including cholera, will increase in frequency and extend to new areas. All of the health impacts of ocean pollution fall disproportionately on vulnerable populations in the Global South – environmental injustice on a planetary scale.

**Conclusions:**

Ocean pollution is a global problem. It arises from multiple sources and crosses national boundaries. It is the consequence of reckless, shortsighted, and unsustainable exploitation of the earth’s resources. It endangers marine ecosystems. It impedes the production of atmospheric oxygen. Its threats to human health are great and growing, but still incompletely understood. Its economic costs are only beginning to be counted.

Ocean pollution can be prevented. Like all forms of pollution, ocean pollution can be controlled by deploying data-driven strategies based on law, policy, technology, and enforcement that target priority pollution sources. Many countries have used these tools to control air and water pollution and are now applying them to ocean pollution. Successes achieved to date demonstrate that broader control is feasible. Heavily polluted harbors have been cleaned, estuaries rejuvenated, and coral reefs restored.

Prevention of ocean pollution creates many benefits. It boosts economies, increases tourism, helps restore fisheries, and improves human health and well-being. It advances the Sustainable Development Goals (SDG). These benefits will last for centuries.

**Recommendations:**

World leaders who recognize the gravity of ocean pollution, acknowledge its growing dangers, engage civil society and the global public, and take bold, evidence-based action to stop pollution at source will be critical to preventing ocean pollution and safeguarding human health.

Prevention of pollution from land-based sources is key. Eliminating coal combustion and banning all uses of mercury will reduce mercury pollution. Bans on single-use plastic and better management of plastic waste reduce plastic pollution. Bans on persistent organic pollutants (POPs) have reduced pollution by PCBs and DDT. Control of industrial discharges, treatment of sewage, and reduced applications of fertilizers have mitigated coastal pollution and are reducing frequency of HABs. National, regional and international marine pollution control programs that are adequately funded and backed by strong enforcement have been shown to be effective. Robust monitoring is essential to track progress.

Further interventions that hold great promise include wide-scale transition to renewable fuels; transition to a circular economy that creates little waste and focuses on equity rather than on endless growth; embracing the principles of green chemistry; and building scientific capacity in all countries.

Designation of Marine Protected Areas (MPAs) will safeguard critical ecosystems, protect vulnerable fish stocks, and enhance human health and well-being. Creation of MPAs is an important manifestation of national and international commitment to protecting the health of the seas.

## Introduction

The oceans are vast. They cover more than 70% of the earth’s surface, hold 97% of the world’s water, host some of the planet’s most diverse ecosystems, and support economies in countries around the world [1,2]. Microscopic organisms in the seas are a major source of atmospheric oxygen [3,4,5,6]. By absorbing more than 90% of the excess heat released into the earth’s environment and nearly one-third of carbon dioxide emissions, the oceans slow planetary warming and stabilize the global climate [7].

The oceans are essential to human health and well-being [8,9,10,11,12,13]. They provide food to billions, livelihoods for millions and are the source of multiple essential medicines [14]. They have traditional cultural value and are a source of joy, beauty, peace, and recreation [15,16]. The oceans are particularly important to the health and well-being of people in small island nations [17], the high Arctic, and coastal communities, especially those in the Global South [1]. The very survival of these vulnerable populations depends on the health of the seas [10,12].

Despite their vast size, the oceans are under threat, and human activity is the main source of the threat [1,2]. Climate change and other environmental disruptions of human origin have caused sea surface temperatures to rise, glaciers to melt, and harmful algal species and pathogenic bacteria to migrate into waters that were previously uncontaminated. Rising seas and increasingly violent coastal storms endanger the 600 million people worldwide who live within 10 m of sea level [1]. Rising concentrations of atmospheric CO_2_ have caused acidification of the oceans, which in turn destroys coral reefs, impairs development of oysters and other shellfish, and dissolves calcium-containing microorganisms at the base of the food web [1,18,19]. The oceans are losing oxygen [1]. Fish stocks are declining [20,21,22]. Dredging, mechanized trawling, oil exploration, and planned deep undersea metal mining threaten the seabeds [23].

Pollution – unwanted, often hazardous waste material released into the environment by human activity – is one of the existential challenges of the present age [24]. Like climate change, biodiversity loss, and depletion of the world’s fresh water supply, pollution endangers the stability of the earth’s support systems and threatens the continuing survival of human societies [8].

Pollution is also a great and growing threat to human health. It is the largest environmental cause of disease in the world today, responsible for an estimated 9 million premature deaths per year [24]. It causes enormous economic losses, undermines national trajectories of economic development, and impedes attainment of the Sustainable Development Goals (SDGs) [22].

Pollution has until recently been overlooked in international development planning and largely neglected in the global health agenda [25]. For too long, pollution has been regarded as the unavoidable price of economic progress [25], a view that arose out of the experience of the 19th and 20th centuries when combustion of fossil fuels – coal in particular – was the engine of economic growth and pollution was seen as unavoidable. Today, however, the claim that pollution is inevitable and that pollution control costs jobs and stifles economies is no longer tenable. It has been disproven by the experience of the many countries that have more than doubled their GDPs in the past half century while greatly reducing pollution [24,25,26]. It has become irrelevant with the increasing availability of low-cost, renewable sources of energy and advances in green chemistry.

Ocean pollution is a critically important but underrecognized component of global pollution [26,27]. It has multiple direct and indirect impacts on human health [28,29,30,31,32,33,34,35]. The nature and magnitude of these effects are only beginning to be understood.

The purpose of this review is to examine the impacts of ocean pollution on human health and well-being, identify gaps in knowledge, project future trends, and offer scientifically based guidance for effective interventions. Information presented in this review will guide attainment of the Sustainable Development Goals (SDGs), in particular, SDG 14, which calls for prevention and significant reduction of all marine pollution, and SDG 3, which calls for improvement of human health and well-being.

The ultimate aim of this report is to increase awareness of ocean pollution among policy makers, elected leaders, civil society and the public and to catalyze global action to monitor, control, and prevent pollution of the seas.

By focusing our analysis on human impacts, we underscore the fact that pollution of the oceans poses a clear and present danger to human health. It is causing disease, disability, and premature death in countries around the world today.

On the positive side, pollution of the oceans is not inevitable. It is a problem of human origin, and the successes in pollution control that have been achieved in many countries show that it can be controlled and prevented.

World leaders who recognize the great magnitude of ocean pollution, acknowledge its grave dangers to human health, engage civil society and the global public, and take bold, evidence-based action will be key to stop ocean pollution at its source and safeguarding human health.

## Methods

This report consists of a series of topic-focused reviews that critically examine current knowledge of each ocean pollutant – its sources, magnitude, geographic extent, populations at greatest risk, and its known and potential effects on human health. We examine the strength of the evidence linking pollutants to health effects [29].

To the extent possible, we consider health effects not only of individual pollutants, but also of the complex mixtures of chemical pollutants and biological contaminants found in the seas today. We examine interactions and synergies among pollution, climate change and ocean acidification. Because the effects of pollution are disproportionately concentrated in low-income countries in the Global South, small island nations, and indigenous populations in the far north [12], we specifically examine ocean pollution’s impacts on these vulnerable populations. Finally, we consider the prospects for prevention and control of ocean pollution and present case studies of success in pollution control.

## Findings

### The Current State of Ocean Pollution

**Figure 1:**
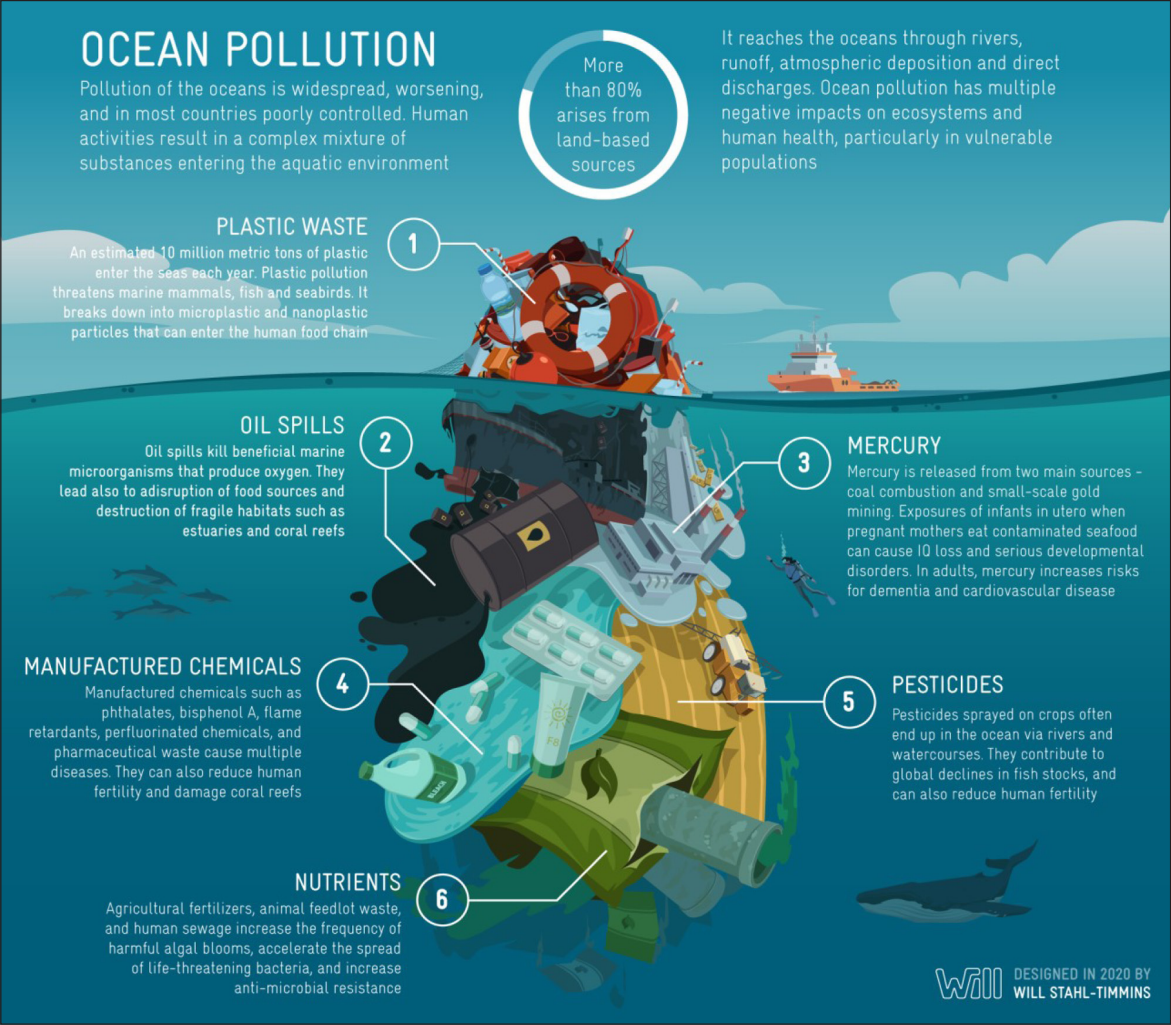
Ocean Pollution – A Complex Mixture.

Pollution of the oceans is widespread, it is worsening, and its geographic extent is expanding [26,27,30]. Ocean pollution is a complex and ever-changing mixture of chemicals and biological materials that includes plastic waste, petroleum-based pollutants, toxic metals, manufactured chemicals, pharmaceuticals, pesticides, and a noxious stew of nitrogen, phosphorus, fertilizer, and sewage (Figure 1).

Some ocean pollutants are “legacy” pollutants, materials deposited in the seas decades ago, while others are new. The relative concentrations of pollutants vary in different regions of the oceans and at different seasons of the year. Plastic pollution is the most visible component of ocean pollution. It is growing rapidly, but it is only the obvious tip of a much larger problem.

Land-based sources account for approximately 80% of ocean pollution, while discharges from marine shipping, offshore industrial operations, and waste disposal at sea account for the remaining 20% [26]. Pollution is most severe along coastlines and in bays, harbors, and estuaries where wastewater discharges, industrial releases, agricultural runoff, and riverine pollution cause massive in-shore contamination. Some of the world’s worst ocean pollution is seen along the coasts of rapidly developing countries in the Global South [26].

The European Environment Agency (EEA) reports that pollution by toxic metals, industrial chemicals and plastic wastes is at problem levels in 96% of the Baltic Sea, in 91% of the Black Sea, in 87% of the Mediterranean Sea, and in 75% of the North-East Atlantic Ocean [27]. Pollution by plastic waste has become a global threat [31].

The drivers of ocean pollution are rapid industrialization; continuing increases in the manufacture and release into the environment of chemicals and plastics; expansion of chemically intensive agriculture; massive releases of liquid and solid waste into rivers, harbors, and estuaries; and insufficient re-use and recycling of feedstock materials [16,32]. Specific sources of ocean pollution are:

- Coal combustion and gold-mining are the two main sources of marine mercury pollution [33].
- Exponential growth in chemical production coupled with inadequate controls on chemical releases are the main drivers of pollution of the oceans by manufactured chemicals [34].
- Marine pollution by plastic waste reflects massive global growth in plastic production, which now exceeds 420 million tons per year [35].
- Uncontrolled economic development and rapid population growth along the world’s coasts has led to pollution of in-shore waters by industrial releases, agricultural runoff and sewage [36,37,38,39]. Many populated coastal areas are now covered by buildings and impervious surfaces, which increases runoff. This runoff as well as discharges of wastewater and storm water, much of it inadequately treated, further increases pollution. The consequences are increasing abundance of pathogenic bacteria, viruses, and parasites [40], eutrophication, and increased frequency and severity of harmful algal blooms (HABs) – “red tides”, “brown tides”, and “green tides” – some of which produce potent disease-causing toxins.

Despite the great magnitude of ocean pollution and growing recognition of its effects on human and ecosystem health, great gaps remain in knowledge about pollution sources, levels of pollution in many areas of the seas, the sizes of high-risk populations, the extent of human exposure, and the magnitude of health effects. Because of these gaps, the impacts of ocean pollution on human health and well-being are underestimated, and it is not yet possible to fully quantify the contribution of ocean pollution to the global burden of disease [41].

### Climate Change, Global Warming, Ocean Acidification, and Pollution

Since the 1970s, the oceans have warmed steadily in concert with global climate change [42]. They have taken up more than 90% of the excess heat released into the climate system [1]. Mean sea surface temperature is rising by 0.13°C per decade [43]. The frequency of marine heatwaves has more than doubled [1].

Further impacts of climate change on the oceans are increases in the intensity and frequency of extreme weather events such as heat waves, heavy rainstorms, and major hurricanes, and changes in large-scale planetary phenomena such as El Niño events [44] and the Indian Ocean Dipole [1,45,46].

Ocean acidification is another consequence of climate change. The oceans absorb nearly one-third of the carbon dioxide (CO_2_) emitted into the atmosphere, and the amount of CO_2_ absorbed by the seas has increased in recent decades as CO_2_ emissions of human origin have increased. Ocean acidification is the result [7]. Since the late 1980s, the surface pH of the open ocean has declined by about 0.1 pH units relative to preindustrial time (i.e., a 26% increase in acidity [hydrogen ion concentration]), and the rate of increase is 0.017–0.027 pH units per decade [1].

Ocean acidification threatens the integrity of coral reefs. It impairs the development of oysters and other commercially important shellfish, thus impacting commercial fisheries. It endangers the survival of calcium-containing microorganisms at the base of the marine food web [1,47]. Ocean acidification may also increase the toxicity of certain heavy metals and organic pollutants [1,48].

Global warming liberates legacy pollutants from ice and permafrost, alters the geographic distribution of chemical pollutants in the oceans, and increases exposures of previously unexposed populations. All of these effects have potential to magnify the ocean pollution’s impacts on human health [49].

**Figure 2:**
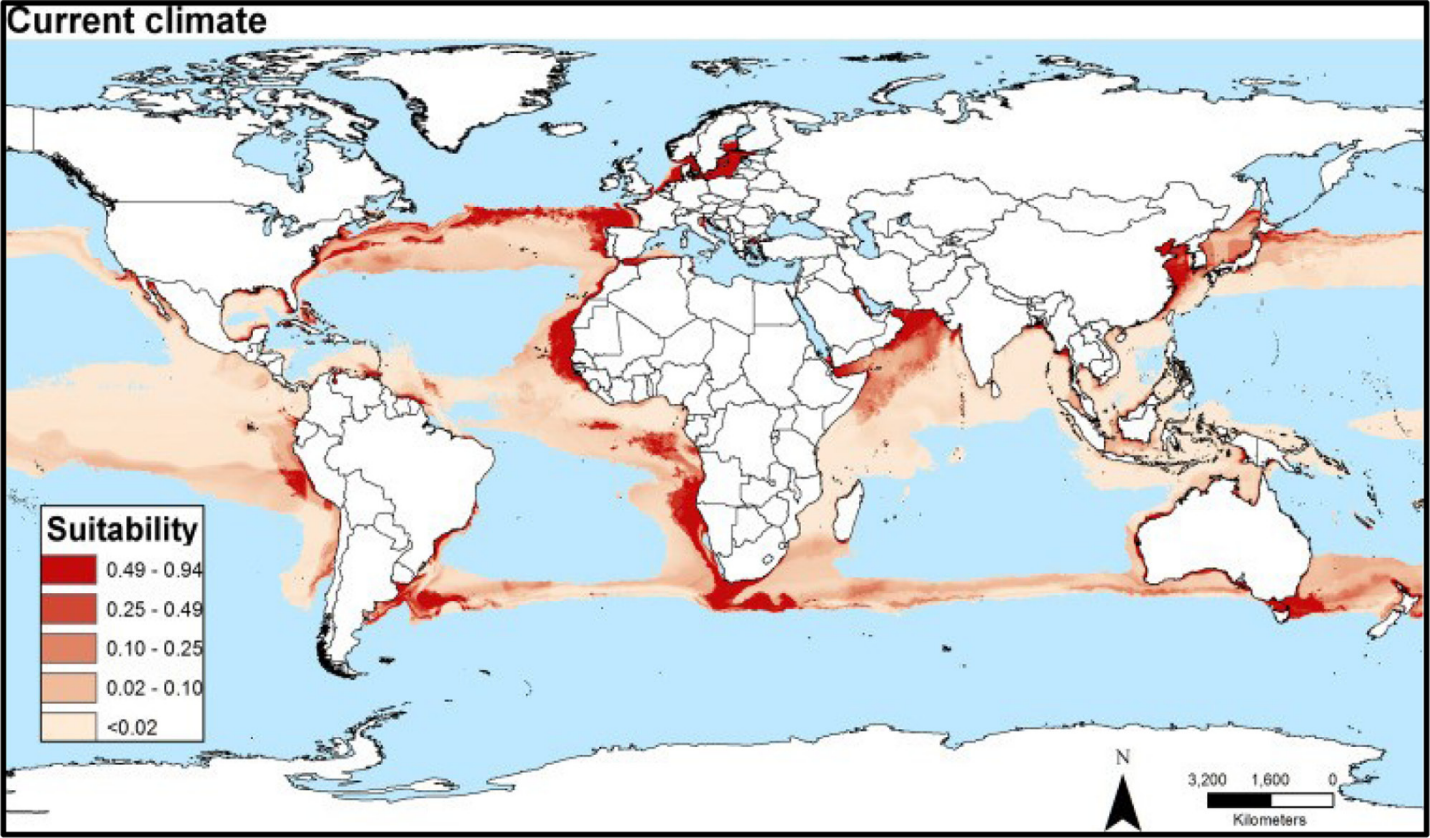
Areas considered suitable for Vibrio cholerae [50]. *Source*: Escobar et al., (2015) (https://doi.org/10.1016/j.actatropica.2015.05.028) CC BY 4.0.

Rising sea surface temperatures and increasing ocean pollution result in greater abundance and expanded geographic ranges of naturally occurring marine pathogens, such as *Vibrio* species, among them *Vibrio cholerae*, the causative agent of cholera [50,51] (Figure 2). The likely consequences will be increases in the frequency of *Vibrio-*associated illnesses and spread of these infections to new, previously unaffected areas. Risk is especially high in low-income countries where coastal development is intense and sanitation systems are dysfunctional due to civil unrest, conflict, sea level rise, coastal over-development, and natural disasters [52].

In a similar manner, climate change, sea surface warming, and ocean pollution appear to be increasing the frequency, severity, and global geographic extent of harmful algal blooms (HABs) [53,54]. Some dangerous algal species are moving poleward in response to the warming of coastal waters [54,55], changes in ocean stratification, alteration of currents, changes in nutrient upwelling, and changes in land runoff and micronutrient availability [56,57]. The likely consequences will be the occurrence of HABs in previously unaffected areas and exposures of previously unexposed populations in the circumpolar regions to HAB toxins.

## Impacts of Ocean Pollution on Human Health

### Chemical Pollutants

#### Toxic Metal Pollutants

Releases of toxic metals to the environment began millennia ago with the inception of mining and smelting. These releases have increased since the beginning of the Industrial Revolution and risen especially in the past two centuries [58,59,60].

**Figure 3:**
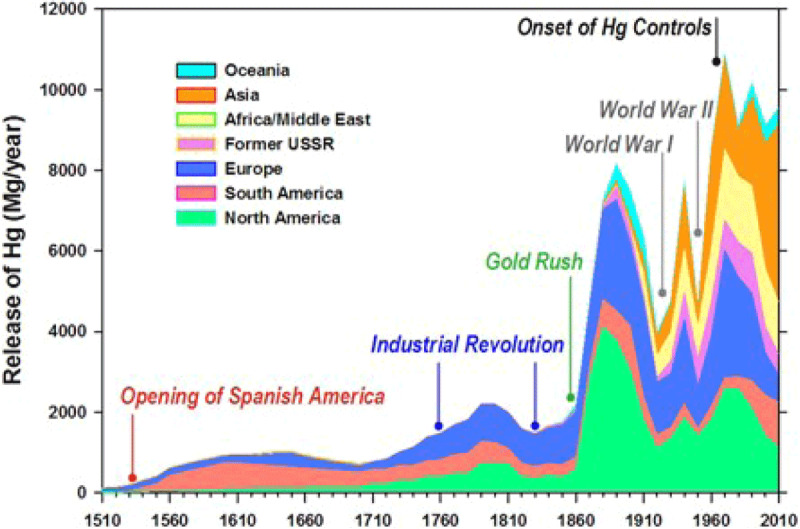
Total global mercury releases and relevant historical factors, 1510–2010. *Source*: Street et al., (2019) (https://doi.org/10.1088/1748-9326/ab281f) CC BY 3.0.

Mercury is the metal pollutant in the oceans of greatest concern for human health [34]. Over the past 500 years, human activities have increased total environmental mercury loading by about 450% above natural background. About 70% of the mercury circulating in the environment today consists of mercury emitted from human sources in the past, termed *legacy mercury* [*61*] (Figure 3). The presence of large quantities of legacy mercury in the global environment and the potential for climate change to remobilize this mercury complicate projections of future exposures and health impacts.

#### Current Sources of Mercury Pollution

An estimated 2,220 tons of mercury are currently emitted to the environment each year as the direct result of human activity. These emissions account for about 30% of current mercury emissions. Another 60% of current mercury emissions result from environmental recycling of anthropogenic mercury previously deposited in soils and water. The remaining 10% comes from natural sources such as volcanoes.

Combustion of coal and artisanal/small-scale gold-mining (ASGM) are the two principal human sources of current mercury emissions. All coal contains mercury and when coal is burned, mercury is released into the atmosphere where it can travel for long distances until ultimately it precipitates into rivers, and lakes and the oceans.

In ASGM, mercury is used to form an amalgam to separate gold from rock. The amalgam is heated to boil off the mercury leaving the gold behind. ASGM operations release mercury to the environment through vaporization and through runoff of spilled mercury into waterways [34]. Metal mining and oil and gas exploration can be additional sources of mercury release. In rivers, lakes and the oceans, the metallic, inorganic mercury released to the environment from these sources is converted by marine microorganisms into methylmercury, an organic form of mercury that is a potent neurotoxicant.

The largest fraction of global mercury emissions – about 49% – originate today in East and South-East Asia. Coal combustion and industrial releases are the major sources there. South America accounts for 18% of global mercury emissions and Sub-Saharan Africa for 16%. In both of these regions, ASGM is the major source of mercury releases.

Methylmercury is a persistent pollutant in the marine environment. It bioconcentrates as it moves up the food web, so that top predator species such as tuna, striped bass and bluefish as well as marine mammals can accumulate concentrations of methylmercury in their tissues that are 10 million or more times greater than those in surrounding waters [34].

**Figure 4:**
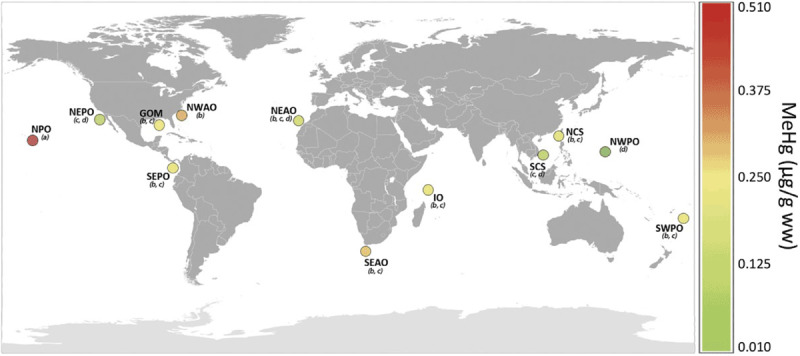
Geographic differences in methylmercury concentrations of yellowfin tuna (*Thunnus albacares*). *Source*: Reprinted from Nicklish et al., Mercury levels of yellowfin tuna (*Thunnus albacares*) are associated with capture location. *Environmental Pollution* 2017: 87–93, doi.org/10.1016/j.envpol.2017.05.070 with permission from Elsevier.

Mercury levels vary substantially in different regions of the ocean. This variation is seen in a recent survey of methylmercury concentrations in yellowfin tuna, in which levels differed by 26-fold around the world. Highest levels were found in tuna from the North Pacific Ocean (Figure 4), and these high concentrations reflect mercury releases from coal-fired power plants and steel mills in Asia that are carried northeastward across the Pacific on the prevailing winds [62,63].

Human exposure to methylmercury occurs primarily through consumption of contaminated fish and marine mammals [34,64] Populations in the circumpolar region are heavily exposed to mercury in their diets – principally in the form of methylmercury – as a consequence of their traditional consumption of a diet rich in fish and marine mammals. Most of the mercury to which these populations are exposed originates from sources far away.

#### Neurobehavioral Toxicity of Methylmercury

The brain is the organ in the human body most vulnerable to methylmercury. This vulnerability is greatest during periods of rapid brain growth – the nine months of pregnancy and the first years of postnatal life [65].

There appears to be no safe level of methylmercury exposure in early human development.

Prospective epidemiological cohort studies undertaken in the Faroe Islands demonstrate that children exposed to methylmercury *in utero* exhibit decreased motor function, shortened attention span, reduced verbal abilities, diminished memory and reductions in other mental functions. Follow-up of these children to age 22 years indicates that these deficits persist and appear to be permanent [66].

A similar study conducted in Nunavik of child development at age 11 years showed that methylmercury exposure in early life is associated with slowed processing of visual information, decreased IQ, diminished comprehension and perceptual reasoning, impaired memory, shortened attention span, and increased risk of attention deficit/hyperactivity disorder (ADHD) [67,68]. Other prospective studies have also reported neurobehavioral deficits in children with elevated prenatal exposure to methylmercury [69].

Mercury exposure later in childhood and also in adolescence can also cause damage because the human brain continues to develop throughout this time [70]. Genetic factors may increase vulnerability to methylmercury in some individuals [71].

#### Accelerated Loss of Neurocognitive Function in Adults Exposed to Methylmercury

Recent studies have shown that adult exposures to methylmercury can also have negative effects on brain function [72]. Thus, in a cross-sectional study of 129 men and women living in six villages on the Cuiaba River in Brazil, elevations in hair mercury concentrations were associated with reductions in motor speed, manual dexterity, and concentration [73]. Some aspects of verbal learning and memory were also impaired. The magnitude of these effects increased with increasing concentrations of mercury in hair. The brain functions disrupted in adults by methylmercury – attention span, fine-motor function, and verbal memory – are similar to those previously reported in children with prenatal exposures but appear to occur at substantially higher levels of exposure.

#### Cardiovascular Effects of Methylmercury Pollution

Elevated concentrations of methylmercury in blood and tissue samples are associated with increased risk for acute coronary events, coronary heart disease, and cardiovascular disease [74]. The US National Research Council concluded in 2000 that methylmercury accumulation in the heart leads to blood pressure alterations and abnormal cardiac function [75].

Subsequent research has strengthened these findings. An expert panel convened by the US Environmental Protection Agency in 2011 concluded that methylmercury is directly linked to acute myocardial infarction and to increases in cardiovascular risk factors such as oxidative stress, atherosclerosis, decreased heart rate variability, and to a certain degree, hypertension [76]. Likewise, a 2017 systematic review found that methylmercury enhances production of free radicals resulting in a long-lasting range of effects on cardiac parasympathetic activity that increase risk for hypertension, myocardial infarction, and death [77]. Further research has confirmed these findings [78,79].

#### The Contribution of Marine Mercury Pollution to the Global Burden of Disease

Efforts have begun to estimate the contribution of mercury pollution of the oceans to the global burden of disease (GBD). A recent estimate finds that between 317,000 and 637,000 babies are born in the United States each year with losses of cognitive function that are the consequence of prenatal exposures to methylmercury resulting from consumption of mercury-contaminated fish by their mothers during pregnancy. These losses range in magnitude from 0.2 to 5.13 IQ points depending on the severity of exposure. These authors found additionally that population-wide downward shifts in IQ caused by widespread exposure to methylmercury are associated with excess cases of mental retardation (IQ below 70), amounting to 3.2% (range: 0.2–5.4%) of all cases of mental retardation in the United States [80].

#### Impacts of Ocean Acidification on Metals Toxicity

The alterations of carbonate chemistry in the seas – i.e. decrease in pH, decrease in [CO_3_^2–^] and increase in [HCO_3_^–^]) – that are the consequences of increasing CO_2_ absorption induce changes in the speciation of metals that alter their solubility and bioavailability and therefore their toxicity [48,81].

For example, by 2100, the projected pH of the oceans will be approximately 7.7, resulting in a 115% increase in the mean free ionic form of copper (Cu^2+^) in certain estuaries [82]. Consequently, the biotoxicity of copper to invertebrates [83] and to plankton photosynthesis and productivity will be enhanced. At the same time, however, ocean acidification will increase the concentration of dissolved iron, which could partially alleviate the inhibitory effect of copper on photosynthesis [84]. Ocean acidification appears in some instances to mitigate [85] or even reduce [86] the toxicity of mercury. As metals may play a role in the biodegradation of organic pollutants, changes in metal speciation could slow these processes and therefore potentiate the toxicity of some organic pollutants [87].

#### Prevention of Mercury Pollution

Evidence has shown that two actions will be key to preventing further addition of mercury to the oceans. These are a cessation of coal combustion and reduction of mercury use in artisanal and small-scale gold mining (ASGM). Cessation of coal combustion will not only slow the pace of climate change and reduce particulate air pollution, but will also greatly reduce atmospheric emissions of mercury and thus reduce additional deposition of mercury into the oceans. ASGM is a major source of mercury pollution of the oceans in the Global South. Actions underway under the aegis of the Minamata Convention are seeking to identify and control major sources of mercury pollution from ASGM [34].

### Plastic Pollution of the Oceans

Plastic waste represents approximately 80% of all marine litter [88]. An estimated 10 million metric tons of plastics – range of estimate, 4.8 to 12.7 million – are released to the oceans each year [89]. The total amount of plastic waste circulating in the world’s oceans is projected to be 150 million tons by 2025 [89,90]. Marine plastic waste ranges in size from floating barrels, plastic bottles and plastic sheets down to sub-microscopic particles and fibers.

**Figure 5:**
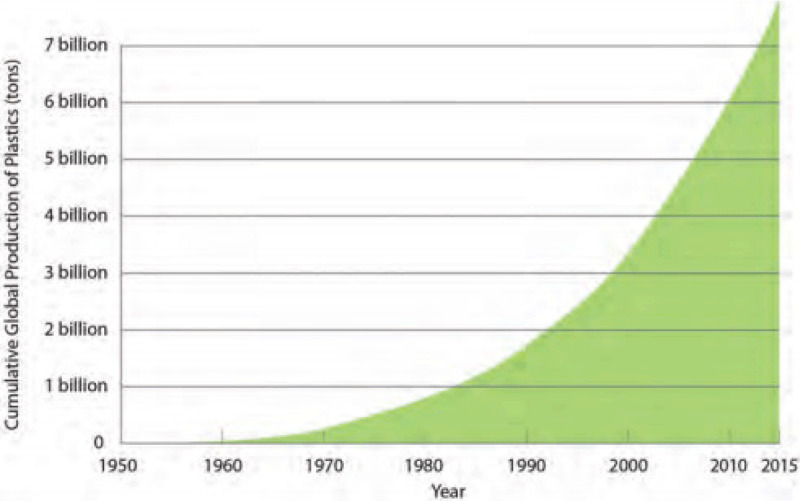
Cumulative Plastic Production since 1960. Calculated as the sum of annual global polymer resin, synthetic fiber, and plastic additive production. Most of this plastic still exists. *Source*: Our World in Data (https://ourworldindata.org/plastic-pollution), CC BY 4.0).

Recent increases in marine plastic pollution reflect massive growth in plastic production (Figure 5), which now exceeds 420 million tons per year. Much of this plastic goes into consumer products, and over 40% is used in products that are discarded within one year of purchase – often after only a single use [91]. The consequence is massive global accumulation of plastic waste [92].

Plastics are produced by the polymerization of highly reactive and often toxic chemical monomers, 98% of them derived from fossil fuels. They are designed to be stable, durable and resistant to degradation [93]. Because of these properties, discarded plastic that reaches the marine environment can persist for decades and travel long distances. Plastic waste is now ubiquitous in surface waters, on the coasts, in estuaries, on the high seas, and even in the deepest and most remote parts of the ocean [94,95,96,97,98,99,100].

#### Sources of Plastic Pollution

The United Nations Joint Group of Experts on the Scientific Aspects of Marine Pollution (GESAMP) [101] estimates that land-based sources account for up to 80% of the world’s marine pollution with 60–95% of this waste comprised plastic debris.

Rivers are a major source of plastic waste in the oceans, and riverine input is estimated to be between 1.15 and 2.41 metric tons per year, corresponding to between 9 and 50% of all plastic transported to the oceans. Rivers draining densely populated, rapidly developing coastal regions with weak waste collection systems are particularly important sources [102], and it is estimated that between 88–95% of marine plastic comes from only 10 rivers [103]. Largest inputs, accounting for approximately 86% of the plastic waste entering the marine environment, are from the coasts of Asia, mainly China [89,104]. Additional sources include aquaculture, fishing and shipping [27].

Plastic wastes are gathered by oceanic currents and collect in five large, mid-ocean gyres located in the North Pacific, South Pacific, North Atlantic, South Atlantic, and Indian Oceans. The North Pacific gyre is a relatively stationary area twice the size of France that has waste from across the North Pacific Ocean, including material from the coastal waters of North America and from Japan.

#### Marine Pollution by Plastic Microparticles

Weathering, mechanical abrasion, and photodegradation break plastic waste in the oceans down into smaller particles termed microplastics (<5 mm in diameter) and still smaller particles termed nanoplastics (<1μm in diameter; defined as <100 nm by some authors) [105,106,107]. The size distribution of ocean microplastics is highly skewed, with increasing numbers of particles at smaller particle sizes [108,109]. Microplastic particles can sink downward through the water column and accumulate on the ocean floor. In contrast to microplastics, which have been measured widely in the marine environment (e.g., **Text Box 1**) and in marine organisms, concentrations of nanoplastics are poorly defined [110,111,112,113,114,115].

**TEXT BOX 1: Microplastic contamination in Massachusetts beaches and blue mussels, *Mytilus edulis*.**

**Background.** Microplastic particles have been increasing in prevalence in the oceans since the late 1900s and are found today on beaches across the world [101,182]. The majority are produced through weathering and fragmentation of larger macroplastics. Toxic and endocrine disrupting chemicals such as phthalates and bisphenol A may be incorporated into plastics during manufacture, and microplastics can also absorb toxic chemicals from seawater. Because of their small size, microplastics are easily absorbed by microscopic marine organisms and thus can enter the food chain where they bioconcentrate [101]. Current studies are examining the possible effects of microplastics on ecosystem dynamics and also on the health of humans who consume fish and shellfish.

**Goal.** The two goals of this study were to examine (1) the physical characteristics, spatial distribution and abundance of microplastics on Massachusetts beaches, and (2) the characteristics of microplastics in wild blue mussels harvested in Massachusetts.

**Methods.** Six Massachusetts beaches were targeted – beaches in and around Boston (high urban density) and in more remote areas (Provincetown, Cape Cod, low population density). Sediment samples were collected from representative beaches and microplastics were prepared by density separation [183]. Blue mussel (*Mytilus edulis*) samples were collected from Provincetown. Samples were prepared following tissue digestion with concentrated KOH [184]. All samples were visualized by standard light microscopy and select samples were further analyzed by Raman spectroscopy.

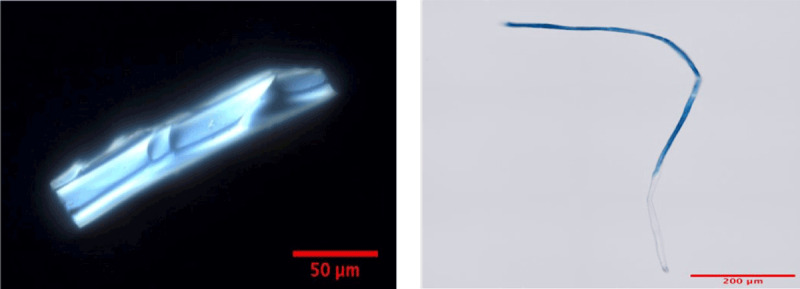

**Findings.** Microplastics were found in all beach samples examined and in most mussels screened. Microplastics in select blue mussel samples showed Raman spectra similar in appearance to those associated with polycarbonate plastics.

**Conclusion.** This study demonstrates that microplastics are ubiquitous on Massachusetts beaches and that they can enter the human food chain through consumption of blue mussels.

**Further studies.** Future studies are targeting additional beaches (including freshwater beaches) and examining species higher on the food chain (crustaceans and fish). Laboratory-based weathering studies are underway to examine the processes involved in microplastic generation. Studies in *Drosophila melanogaster* are examining the effects of off-the-shelf and laboratory-generated microplastic exposure via feeding on behavior, phenotype and gene expression.

Microplastics are also manufactured. They are produced in the form of microplastic beads – polystyrene spheres 0.5 to 500 μm in diameter. These beads are used in industrial processes such as 3D printing. They also have multiple applications in human and veterinary medical products to enhance drug delivery to tissues, and in cosmetics such as toothpaste, abrasive scrubbers and sunscreen. Manufactured microplastic beads are released to the environment from these products. They enter the oceans by way of urban runoff, sewage discharge, and direct wash-off of cosmetics and sunscreens from the skin of swimmers and surfers.

Microplastics degrade in the marine environment at varying rates depending on the core material and weathering conditions. Some petroleum-based plastics can take hundreds of years to degrade, although under some circumstances photochemical degradation can be significant [97,116,117].

Microplastic particles contain substantial quantities of toxic chemicals. Toxic chemical additives are incorporated into plastics during their manufacture to convey specific properties such as flexibility, UV protection, water repellence, or color [118,119,120,121,122]. These additives can comprise as much as 60% of the total weight of plastic products. They include plasticizers such as phthalates, brominated flame retardants, antioxidants, UV stabilizers, and pigments [106,123]. Due to their large surface-to-volume ratio, microplastic particles can also adsorb toxic chemical pollutants from the marine environment – polycyclic aromatic hydrocarbons (PAHs), PCBs, DDT, and toxic metals [106].

Some plastic additives such as synthetic dyes, are classified as mutagens and carcinogens [124,125,126]. Others such as bisphenol A and phthalates are endocrine disruptors – chemicals that can mimic, block, or alter the actions of normal hormones. Perfluorinated additives, widely used in plastic to make them water-repellent, are deleterious to human reproduction. Still other plastic additives can reduce male fertility and damage the developing human brain [127,128]. Also of concern are residual unreacted monomers and toxic chemical catalysts that may be trapped in plastic during its manufacture.

Chemical additives and adsorbed chemicals can leach out of microplastic and nanoplastic particles. They can enter the tissues of marine organisms that ingest these particles, including species consumed by humans as seafood. Concentrations of some chemical additives have been found to be orders of magnitude higher in microplastic particles than in surrounding seawater [129].

#### Marine Pollution by Plastic Microfibers and Tire-Wear Particles

Microfibers and tire-wear particles are distinct sub-categories of microplastics. Microfibers originate mainly from the clothing and textile industries [130,131,132]. Tire-wear particles are formed by the abrasion of car and truck tires. These materials reach surface waters and ultimately the oceans through runoff from roadways [133,134,135].

Plastic microfibers are distributed globally in both water and air [129,136,137,138]. They have become ubiquitous in all ecosystems. They are found in seafood [139,140]. Humans can be exposed to microfibers through consumption of contaminated fish or shellfish. Inhalation of airborne microfibers may represent an even greater source of human exposure [141,142].

#### Effects of Plastic Pollution on Marine Species

Elucidation of the toxicological impacts of microplastics, including microfibers, is challenging because of their heterogeneity and great complexity [106]. Microplastics span a wide range of sizes and shapes, they are comprised of various polymer materials, and as noted above they contain myriad chemical additives, the identity of which may be proprietary and therefore not generally known. Once in the marine environment, plastics undergo weathering and adsorb additional contaminants, further enhancing their complexity. Finally, marine species exhibit a range of sensitivity to microplastics [143]. All of these factors complicate assessments of toxicity and health hazard [144,145].

Although there is evidence for transfer of additives and adsorbed chemicals from plastics to organisms, the relative contribution of plastics to total chemical exposure by all pathways is thought in most situations to be minor [146,147,148,149,150,151,152]. Likewise, although some additives and sorbed contaminants are able to bioaccumulate and biomagnify in aquatic food webs, there is not yet strong evidence that plastic particles themselves are able to undergo biomagnification [153].

Microplastics have potential to harm living organisms through several mechanisms:

***Physical toxicity*.** Macroscopic plastic wastes, such as bottle caps, small bottles, and food packaging, can be ingested by fish, seabirds, and marine mammals that mistake them for food. Undigested plastic accumulates in these animals’ gastrointestinal tracts where it can cause obstruction that leads to malnutrition, reproductive impairment and death [129,154,155,156,157,158,159,160]. Marine species can also be harmed and killed by becoming entangled in abandoned fishing gear, plastic nets and plastic rings that are caught on reefs or drifting in the water column. An estimated 5.7% of all fishing nets, 8.6% of all traps, and 29% of all lines are lost each year [161,162].

Plastic pollution is a threat to coral reefs [163]. Large plastic debris such as plastic bags and sheeting can smother coral colonies by preventing light from reaching the phototrophic organisms that build reefs and can also cause physical damage.

***Particle effects.*** Microplastics can harm living organisms by virtue of their ability to damage cells, injure tissues, and cause inflammation [164]. While microplastics cannot easily pass through cell membranes, nanoplastic particles can cross the gut lining and accumulate in tissues [165,166,167] where they may have the potential to cause deleterious effects [168]. Leachates containing tire-wear particles have been associated with storm water-associated mortality in salmon [169].

***Chemical Toxicity.*** The toxic chemical additives and the sorbed pollutants in and on microplastics and nanoplastics can leach from plastic particles and enter the tissues of marine organisms [123,170,171,172]. Although plastic particles may not be a major source of chemical exposure [146,147,148,149,150,151,152], there is evidence that in some instances they can be significant contributors to chemical body burden [173].

The challenges associated with assessing the impacts of microplastics on marine organisms are evident in the divergent results of studies reported to date. A recent meta-analysis and review of published research on the effects of microplastics and macroplastics found similar numbers of positive and negative results [174]. A major conclusion from this and other reviews is that most of the experimental work to date has been done using concentrations of microplastics that are not environmentally relevant [144,174,175]. Future research should be conducted under more environmentally relevant conditions [174].

#### Microplastics as Vectors for Microbial Pathogens

An additional hazard of microplastic particles and fibers in the marine environment is that they can transport and shelter hazardous microorganisms, including vectors for human disease [176]. Pathogenic bacteria have been detected on sub-surface microplastics comprised of polyethylene fibers, in plastic-containing sea surface films, and in polypropylene fragments sampled in a coastal area of the Baltic Sea [177]. Similarly, *E. coli* and other potentially pathogenic species have been found on plastics in coastal waters [178] and on public beaches [179]. Algal species involved in HABs [180] and ciliates implicated in coral diseases [181] have also been found attached to marine microplastics.

These findings suggest that harmful microbes and algae that colonize plastics in the marine environment may use microplastic particles to expand their geographical range (‘hitch-hiking’). Adhesion to marine plastic may also enable pathogens to increase their anti-microbial resistance thus facilitating their spread to new areas where they may cause disease and death in previously unexposed populations [177].

#### Human Exposure to Plastic Pollution in the Oceans

Consumption of contaminated fish and shellfish is a major route of human exposure to marine microplastics and their chemical contaminants [140,184,185]. Microplastic and nanoplastic particles are ingested by filter-feeders such as oysters and mussels that are then consumed by humans. Microplastic particles are found also in finfish that have consumed smaller organisms below them in the food web whose tissues are contaminated by microplastics and nanoplastics [123]. Greatest risks of human exposure are associated with consumption of small fish such as sardines that are eaten whole, including the gut [186]. The risk of microplastic ingestion may be especially great in fishing communities and in indigenous populations who rely heavily on seafood and marine mammals for their diet.

A recent study based on assessment of commonly consumed food items estimates that an average person consumes between 74,000 and 121,000 microplastic particles per year [161]. Particle consumption varies by age, sex and diet. Microplastic particles have been detected in human stool samples with about 20 particles detected per 10g of stool, indicating that these particles can reach the human gut [187]. Ingestion of contaminated drinking water and inhalation of airborne microplastic fibers are additional sources of human exposure, and inhalation may be an especially important source [138,141].

#### Human Health Effects of Plastic Pollution in the Oceans

The risks that marine microplastics may pose to human health are not yet well understood and uncertainty about their potential hazard is high [125,186,188,189]. A recent review by SAPEA, an arm of the European Academies of Science, concluded that at present there is “no evidence of widespread risk to human health” of marine plastic pollution [124]. This report goes on to state, however, that as disposal of plastic waste into the oceans continues to increase and more knowledge becomes available, the assessment could change [125,126,128].

Protection of human health against the potential hazards of marine plastic requires a precautionary approach. While current knowledge of health hazards is incomplete, there is sufficient information to justify urgent action to prevent the continuing discharge of plastic waste into the oceans [190,191].

### Pollution of the Oceans by Manufactured Chemicals

More than 140,000 new chemicals have been invented and manufactured in the past 75 years. These synthetic chemicals are largely produced from fossil fuels – coal, oil, and increasingly, gas. Some are used in the manufacture of plastics. Others are incorporated into millions of consumer goods and industrial products ranging from foods and food packaging to clothing, building materials, motor fuels, cleaning compounds, pesticides, cosmetics, toys, and baby bottles [37].

**Figure 6:**
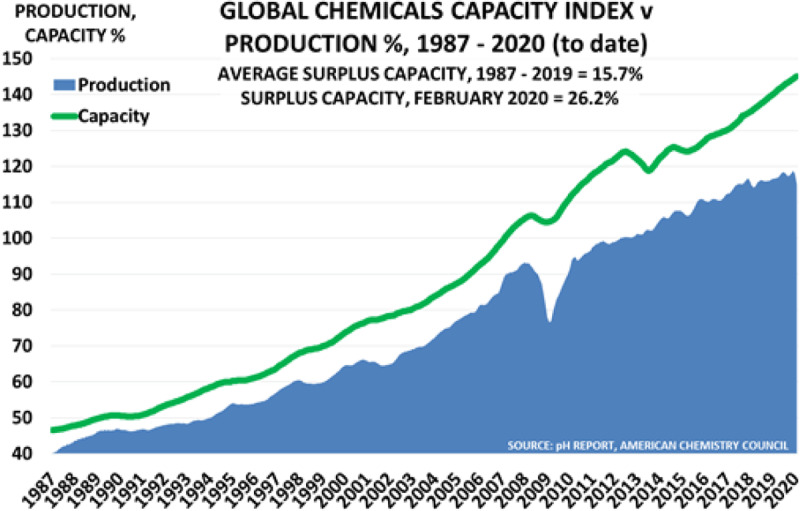
Global Chemical Production and Capacity Index (%) 1987–2020. *Source*: The pH Report, American Chemistry Council.

Global chemical manufacture is increasing by about 3.5% per year and is on track to double by 2045 (Figure 6). More than 60% of current chemical production is in low- and middle-income countries [192], where health and environmental protections are often scant and waste disposal not well controlled.

Manufactured chemicals have become widely disseminated in the environment and are found today in the most remote reaches of the planet [193]. Humans are exposed to these chemicals. In national surveys conducted across the United States by the Centers for Disease Control and Prevention, measurable quantities of more than 200 manufactured chemicals are routinely detected in human tissues [194].

The majority of manufactured chemicals have never been tested for safety or toxicity. Their potential to damage ecosystems or harm human health is therefore not known. In most countries, manufactured chemicals are allowed to enter markets with little scrutiny. Some are found belatedly – sometimes only after years or even decades of use – to have caused damage to planetary support systems (**Text Box 2**), or injury to health. Examples include DDT, asbestos, tetraethyl lead, and the chlorofluorocarbons. Even less is known about the possible combined effects of exposures to mixtures of manufactured chemicals [1,2,34,195].

The thousands of manufactured chemicals that pollute the world’s oceans are variously classified by source (e.g. industrial), chemical structure (e.g. polycyclic aromatic hydrocarbons [PAHs]), intended use (e.g. pesticides; flame-retardants; pharmaceuticals), and environmental and biological properties (e.g., persistent, bioaccumulative), and by mode of toxicity (e.g., endocrine disruptors) [196]. Many are “legacy” pollutants, deposited in the seas over decades, while others are newly recognized.

#### Major Classes of Marine Chemical Pollutants

- ***Halogenated aromatic hydrocarbons (HAHs):*** This group includes most of the chemicals known as persistent organic pollutants (POPs). The best-known members of the group are the polychlorinated and polybrominated biphenyls (PCBs and PBBs), polychlorinated dibenzo-*p*-dioxins (PCDDs) and dibenzofurans (PCDFs), polybrominated diphenyl ethers (PBDEs), and organochlorine (OC) pesticides such dichlorodiphenyltrichloroethane (DDT). These and other POPs are the focus of international efforts to restrict their production and use, such as the Stockholm Convention [199].
  PCBs are mixtures of related chemicals that are resistant to extreme temperature and pressure. In the past, PCBs were used widely in electrical capacitors and transformers, in hydraulic fluids, as heat transfer fluids, lubricants, and as plasticizers. Although production has been banned since the 1970s and 1980s, massive quantities are still present in electrical generators and capacitors and still larger amounts persist in the environment as legacy pollutants. PBBs and PBDEs have been used as flame retardants.
  Dioxins, including the highly toxic 2,3,7,8-tetrachlorodibenzo-*p*-dioxin (TCDD), and furans are by-products formed in the synthesis of chlorinated industrial chemicals and formed also in the incineration of PCBs, polyvinyl plastics, and other manufactured chemicals containing halogens.
  Although the HAHs of greatest concern are manufactured chemicals, the marine environment is also a rich source of naturally occurring HAHs, including hydroxylated PBDEs, halogenated bipyrroles, and halogenated indoles [200].
- ***Perfluoroalkyl substances (PFAS):*** This group contains hundreds of related compounds, all containing fluorine atoms on a carbon backbone. They are used in manufacture of a wide range of products, including non-stick cookware, stain-repellant carpets and furniture, water-repellent clothing, and firefighting foam. PFAS chemicals are highly persistent in the environment. They have caused extensive contamination of surface waters and groundwater, especially near airports and military bases where large quantities were used in firefighting foams. PFAS compounds have entered the oceans in substantial quantities and like other persistent chemicals have been incorporated into the marine food chain.
- ***Organophosphorus flame retardants (OPFRs):*** As the persistence and toxicity of first-generation flame retardants such as PBBs and then PBDEs became known, manufacturers turned to OPFRs, which have now also come to be contaminants in marine ecosystems.
- ***Polynuclear aromatic hydrocarbons (PAHs):*** These are multi-ring compounds that occur naturally in petroleum and oil products and also are generated as soot during incomplete combustion of organic material. Alkylated PAHs are common in petroleum.
- ***Pesticides:*** The term ‘pesticides’ encompasses insecticides, fungicides, and herbicides. These are a large and diverse group of manufactured chemicals designed to be toxic to target organisms (“pests”). Common classes of insecticides are organochlorines (e.g., DDT, and its metabolite DDE), organophosphates, carbamates, and pyrethroids. Herbicides include phenoxyacetic acids (2,4-D and 2,4,5-T), atrazine, and glyphosate.
- ***Organometals:*** Alkylated tin products, especially phenyltin compounds, were commonly used as antifouling agents added to marine paints used on the hulls of ships to prevent growth of barnacles.

#### Spatial and Temporal Distribution of Marine Chemical Pollutants

The oceans are the ultimate sink for chemical pollutants, and persistent pollutants that enter the seas from land-based sources will stay in the oceans for years and even centuries [201].

Concentrations of contaminants vary in different parts of the oceans. Therefore, tracking the levels, fate and geographic distribution of chemical pollutants is a fundamental prerequisite to predicting patterns of exposure, evaluating health effects, and designing evidence-based strategies for pollution control and disease prevention.

With the exception of crude oil, almost all of the chemical contaminants considered in this report originate on land and are transported to the ocean through atmospheric transport, river deposition, runoff, and direct discharges to the seas. In the oceans, pollutant concentrations are influenced by proximity to source, global transport patterns, and marine ecology. Highest concentrations tend to occur near population centers, industrial areas, and centers of industrialized agriculture such as concentrated animal feeding operation (CAFOs). Large-scale changes in ocean temperature and circulation induced by global climate change appear to be important drivers of pollutant distribution [202].

Atmospheric transport is a major factor governing the movement of certain manufactured chemicals from land-based sources to the sea [203]. For example, several classes of persistent organohalogen compounds, such as PCBs and fluorinated compounds volatilize at equatorial and temporal latitudes, move poleward in the atmosphere, and then precipitate to land and in water in the cool air of the polar regions, a phenomenon termed “atmospheric distillation” [204,205]. The consequences are high concentrations of persistent pollutants in marine microorganisms in the circumpolar regions as well as in top predator fish species and marine mammals. Indigenous peoples in the far north who rely heavily on marine species for food are therefore placed at high risk of exposure to POPs.

Direct dumping of industrial wastes into the sea is another source of pollution by toxic chemicals. For example, an estimated 336,000–504,000 barrels of acid sludge waste generated in the production of DDT have been dumped into the Southern California Bight [206]. The disposal process was sloppy and the contents of the barrels readily leaked leading to localized contamination. Once they are in the seas, chemical wastes can be further mobilized through natural or human-caused disturbances. For example, PCBs [207] in the Southern California Bight [206] have been mobilized by dredging of contaminated sediments from San Diego Bay.

Leaching from plastic waste is another route by which toxic chemical pollutants can enter the seas. As was described in the preceding section of this report, a wide range of toxic chemicals can leach out of the 10 million tons of plastic waste deposited in the oceans each year. These manufactured chemicals can enter the marine food chain, thus potentially resulting in ecosystem effects and human exposure.

Global efforts to reduce or eliminate pollution have resulted in some successes in control of ocean pollution, for example in reductions in PCBs and mercury in the seas surrounding Europe (EEA) [27,208]. In general, however, halogenated organic compounds, such as those governed by the Stockholm Convention, are highly resistant to degradation in the marine environment, and these persistent legacy pollutants remain widespread in marine environments.

#### Human Exposure to Marine Chemical Pollutants

An estimated 1–3 billion people depend on seafood as their principal source of dietary protein. Thus, contaminated seafood is the major route of human exposure to marine pollutants. The chemical pollutants most often identified in seafood are methylmercury, PCBs, dioxins, brominated flame retardants, perfluorinated substances, and pesticides.

**Figure 7:**
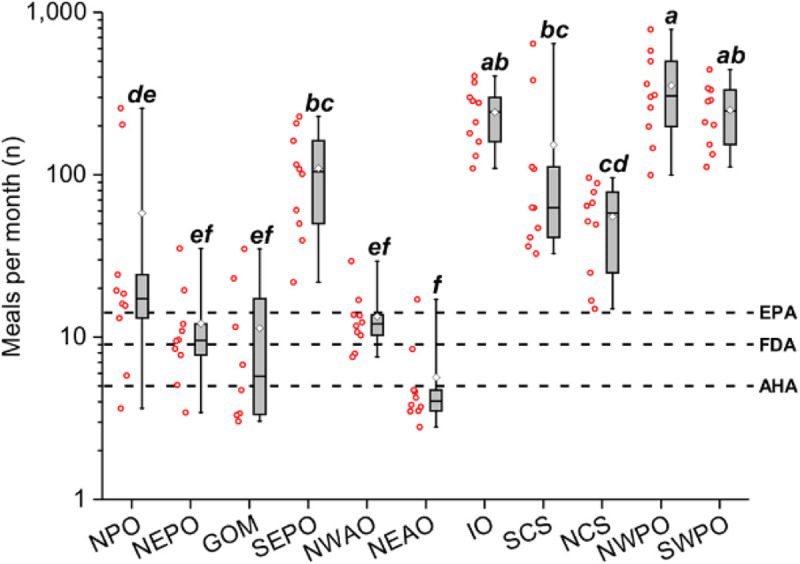
Impact of geographic variation on risk-based fish consumption advisories. Ranges of risk-based consumption limits for 11 sites, calculated in meals per month and based on multiple contaminant exposure with cancerogenic health endpoints, including total PCBs (n = 209), toxaphene and dieldrin. The red hollow spheres to the left of each box plot display the individual fish values. Letters in parenthesis represent subgroups of the sample population with means that were significantly different from each other using Tukey’s post hoc analysis. The U.S. Food and Drug Administration (FDA) and American Heart Association (AHA) recommended minimum monthly fish consumption levels and the U.S. Environmental Protection Agency (EPA) threshold for unrestricted (>16) fish meals per month are shown as dashed lines. Note: GOM, Gulf of Mexico, IO, Indian Ocean; NCS, North China Sea; NEAO, Northeast Atlantic Ocean; NEPO, Northeast Pacific Ocean; NPO, Northern Pacific Ocean; NWAO, Northwest Atlantic Ocean; NWPO, Northwest Pacific Ocean; SCS, South China Sea; SEPO, Southeast Pacific Ocean; SWPO, Southwest Pacific Ocean. *Source*: Nicklisch et al. (2017), https://doi.org/10.1289/EHP518.

Factors that influence concentrations of chemical pollutants in fish include geographic origin, fish age, fish size, and species. Geographic origin is a highly important determinant of pollutant load [209,210,211] and often outweighs the influence of other factors (Figure 7). Thus, fish that live and are caught near cities and major points of pollutant discharge typically contain highly elevated concentrations of POPs and other chemicals [193].

Predator fish species at the top of the food web generally accumulate higher concentrations of chemical pollutants than fish at lower trophic levels. Therefore, fish consumption advisories typically focus on limiting consumption of predator species. However, given the vast scale of the oceans and wide geographic variation in pollutant concentrations, it is perhaps not surprising that that these advisories do not always adequately protect consumers. For instance, one survey found that sardines, a species relatively low on the marine food web, can have higher concentrations of PCBs than cod or salmon [212].

#### Human Health Consequences of Marine Chemical Pollutants

Toxic chemical pollutants in the oceans have been shown capable of causing a wide range of diseases in humans. Toxicological and epidemiological studies document that toxic metals, POPs, dioxins [213], plastics chemicals, and pesticides can cause cardiovascular effects, developmental and neurobehavioral disorders, metabolic disease, endocrine disruption, and cancer (detailed references are provided in the following paragraphs). Effects in humans and laboratory animals are generally similar. Independent, systematic reviews undertaken by the US National Academy of Medicine and the International Agency for Research on Cancer confirm and validate these findings [214,215].

Appendix Table 1 in the Supplementary Appendix to this report summarizes the known links between exposures to toxic chemicals in the oceans and a range of human health outcomes. Key associations are the following:

- ***Cardiovascular disease.*** Multiple toxicological and epidemiologic studies indicate that PCBs, dioxins, PBDEs, OPs, OCs, PAHs and petroleum pollutants, can increase cardiovascular risk factors, including hypertension and atherosclerosis [216,217,218,219], and increase prevalence of cardiovascular disease, stroke, and heart failure. Powerful prospective cohort studies, such as the Nurses’ Health Study II and the Prospective Investigation of the Vasculature in Uppsala Seniors (PIVUS) study [220] provide compelling evidence that POPs exposures in humans are associated with a broad range of cardiovascular conditions.
- ***Developmental defects:*** The core concept of developmental toxicity is that that exposures to extremely low doses of toxic chemicals during windows of exquisite vulnerability in early development can have devastating, potentially lifelong effects on health [221]. Genetic imprinting appears to be a mechanism by which toxic exposures during vulnerable periods injure health and increase risk of disease [222,223]. The Developmental Origin of Human Adult Diseases (DOHAD) hypothesis encapsulates this concept [224], and DOHAD is now recognized to be a widespread phenomenon that explains the toxicity of many manufactured chemicals [225,226]. Some developmental toxicants act by disrupting endocrine function while others directly damage developing organs such as the lungs and the brain.
  The first well-described example of the unique susceptibility of infants and children to toxic chemicals in the environment was in the Minamata disaster in post-war Japan. In Minamata, prenatal exposures of human infants *in utero* to high concentrations of methylmercury in contaminated fish consumed by their mothers during pregnancy caused profound neurological impairment. The mothers, by contrast, sustained little or no physical toxicity [227].
  Manufactured chemicals now recognized to be developmental toxicants include:
  - PCBs and dioxins, which have been linked to neurological, behavioral, and metabolic effects [228,229] and also to reduced fetal growth and low birth weight [230].
  - PBDEs, which have been linked to cognitive impairment in children [231].
  - Phthalates, which are linked to reduced birth weight [232], behavioral abnormalities resembling attention deficit/hyperactivity disorder (ADHD), reproductive abnormalities in baby boys and decreased male fertility [233,234].
  - Bisphenol A, which is linked to behavioral disturbances in childhood [235].
  - Organophosphate compounds, which are associated with reduced head circumference at birth (a measure of delayed brain development), developmental delays, cognitive impairments, and autism spectrum disorder (ASD) [236,237,238].
  - Perfluorinated compounds, such as PFOA and PFOS, which have been linked to decreased fetal growth [239,240], decreased birth weight, reduced head circumference in newborn infants and increased risk of ADHD [241]. Exposures to PFAS compounds are associated additionally with hepatic toxicity, increases in serum lipid levels, increased risk of thyroid disease, suppression of immune function [242], and decreased fertility [239,240,243].
  - *p, p’-*DDE, the principal metabolite of the insecticide, DDT, which affects birth weight [232].
  - Organotin compounds, used extensively in anti-fouling marine paints, have been linked to neurotoxicity, hepatotoxicity, and renal toxicity as well as to ecosystem harm [244].
- ***Developmental neurotoxicity:*** The developing human brain is extremely sensitive to chemical toxicity. Damage done to the brain early in development can become evident at any point in infancy, in childhood, or later in life [245,246,247]. Systematic reviews have now linked early life exposures to several POPs and pesticides (e.g., OP pesticides) [248] to cognitive deficits, ADHD, and autism. Ongoing prospective cohort studies continue to identify new, previously unsuspected chemical causes of developmental neurotoxicity.
  Analysis of NHANES data suggests that PBDE exposure in early life is a major contributor to the burden of intellectual disability in children, resulting in loss of 162 million IQ points and more than 738,000 cases of intellectual disability [249] in the United States each year.
  Prenatal and adult exposures to PCBs are linked to a series of adverse neurodevelopmental outcomes related to cognition – IQ loss and deficits in language, memory and learning – as well as to problems in attention, behavior, executive function, and social behavior. Early-life exposures to PCBs have been associated also with increased risk for attention-deficit hyperactivity disorder (ADHD) and autism spectrum disorder (ASD) [215].
  The consequences of developmental neurotoxicity in early life appear to persist across childhood and adolescence and even into adult life [250]. Thus, the association between prenatal PBDE exposure and attention problems persists at least to age seven years [251]. Likewise, early exposures to PCB 153, DDE, β-HCH, and PFOS are associated with hyperactivity up to at least age 13 years [241,252]. Postnatal exposures may also contribute to these effects and post-natal exposure to PCBs are linked to deficits in fine motor function in Inuit children at age 11 years [253].
- ***Endocrine disruption:*** An endocrine disruptor is defined as “an exogenous substance that causes adverse health effects in an intact organism, or its progeny, secondary to changes in endocrine function” [254]. A number of manufactured chemicals have been found capable of damaging human and ecosystem health through disruption of endocrine function. Chemicals or chemical mixtures can interfere with natural hormones by blocking, mimicking, or disrupting their actions in development, in maintenance of homeostasis and in physiologic function [128].
  Many POPs are EDCs. Because they are environmentally persistent, these chemicals can continue cause damage to living organisms for years or even decades after their release to the environment [255]. Two examples are DDE, the stable metabolite of DDT and PCBs. Both DDT and PCBs have been banned for several decades, but both are still identified in most human blood, milk, and adipose tissues as well as in top predator fish species and marine mammals.
- ***Immune toxicity:*** Halogenated aromatic hydrocarbons, in particular dioxin and dioxin-like compounds have long been known to have harmful effects on the immune system in animals and humans, especially in the embryonic/developing stages [256,257,258]. Evidence suggests that these effects may persist into adolescence and adult life [259]. Some of the less highly persistent PAHs may also have immune effects [260]. Recent evidence indicates that PBDEs and PFAS also have negative effects on human immune function [261,262]. Thus, deficient vaccine antibody responses at age five years were associated with PFAS exposures prenatally and during early infancy [242]. Susceptibility to infectious diseases may also be increased.
- ***Increased Risks of Metabolic Syndrome and Diabetes:*** Consistent associations have been reported between several POPs and increased risk for diabetes and the metabolic disorder [263]. Altered lipid metabolism is another outcome linked to several POPs. A review of health effects linked to PFAS exposure identified dyslipidemia as the strongest metabolic outcome [262]. PCBs have been identified as possibly diabetogenic in the Nurses’ Health Study II [264]. A study in young adults examined changes in metabolism over a 23-year follow-up from exposure [265]. The findings suggest that PCBs and OCPs effects on glucose homeostasis may worsen after decades of exposure to background environmental levels.
- ***Carcinogenesis:*** Numerous toxicological and epidemiological studies have established that many PAHs are carcinogenic, and these studies have also elucidated many of the underlying biochemical mechanisms [266,267]. PAHs are proven human carcinogens and are linked to multiple human cancers, including lung cancer, skin cancer, and bladder cancer [268]. Rodent bioassays conducted by the US National Toxicology Program (NTP) have concluded that PCBs and dioxins are carcinogenic. Occupational and military exposures to these compounds are linked to increased incidence rates of lymphatic cancers, especially Non-Hodgkin’s Lymphoma (NHL), and also to diabetes [269]. Meta-analysis of results from the Yusho and Yu-Cheng cohorts report elevated lung, liver, and all cancers 30 to 40 years after prenatal poisoning by PCBs, chlorinated dioxins, and furans [270].
- ***Mortality:*** Studies in the PIVUS cohort suggest that mortality due to CVD is associated with higher body burdens of POPs [220]. In the US NHANES survey, some organochlorine pesticides have been found to be associated with increased all-cause mortality and others with increased non-cancer, non-cardiovascular mortality [271]. Higher concentrations of POPs in plasma are associated with decreased survival of patients with amyotrophic lateral sclerosis (ALS) [272]. Kim et al. found that an interaction between POPs concentrations and total body fat mass affected risk of mortality from chronic diseases [273]. Massive exposures in early life to PCBs, dioxins, and furans in the Yusho and Yu-Cheng episodes in Japan and Taiwan have been linked to increased risk of mortality from chronic diseases [273] and to elevated all-cause mortality [234,270].

**Table 1:** Major Oil Spills [299].

Spill	Year	Description

VLCC *Metula* Oil Spill, Chile	1974	A very large crude carrier hit a shoal in the Straits of Magellan and released nearly 200,000 tons of light Arabian crude oil.
*Amoco Cadiz* Oil Spill, France	1978	A very large crude carrier clipped shallow rocks off the coast of Brittany. The resulting oil slick polluted 200 miles of the French coast and significantly harmed wildlife (mollusks, crustaceans, birds).
*Atlantic Empress* Oil Spill, Trinidad	1979	Occurred 10 miles off the coast of Trinidad and Tobago. An estimated 90 million gallons of oil were released into the Atlantic Ocean.
Ixtoc Oil Spill, Mexico	1979	Spill occurred as a result of an explosion. 140 million gallons of oil were released into the Gulf of Mexico.
*Exxon Valdez* Oil Spill, Alaska, USA	1989	Released 37,000 metric tons of crude oil into Prince William Sound, Alaska, USA. Considered the worst oil spill worldwide in terms of environmental damage.
Persian Gulf War Oil Spill	1991	Between 252 and 336 million gallons of oil were released into the Persian Gulf during the Gulf War.
Deepwater Horizon Oil Spill, Texas, USA	2010	134 million gallons of crude oil were released into the Gulf of Mexico following an explosion and fire on a drilling platform.
Guarello Island, Patagonia, Chile	2019	40,000 liters of diesel fuel released into the Straits of Magellan from a mining operation.

#### Ocean Pollution by Pharmaceuticals and Personal Care Products (PPCPs)

More than 10,000 chemicals are used in the manufacture of pharmaceuticals and personal care products (PPCPs). These products include therapeutic drugs with both medical and veterinary applications, cosmetics, and cleaning products. They are a subset of the manufactured chemicals discussed in the preceding section. Like pesticides, pharmaceuticals are specifically designed to have biological effects, and thus even low-dose exposures can affect living organisms, including humans.

With increasing manufacture and use of pharmaceuticals by a growing global population, pharmaceutical wastes have entered ecosystems in increasing quantities. Pharmaceutical and cosmetic manufacturing plants, hospitals, nursing homes, confined animal feeding operations (CAFOs), and aquaculture can all release PPCPs into wastewater systems, rivers, and eventually the oceans. Environmentally persistent pharmaceutical pollutants (EPPPs) have been recognized as a “new and emerging issue” under the United Nations’ Strategic Approach to the International Management of Chemicals (SAICM) since 2015.

Therapeutic drugs commonly found in measurable quantities in urban wastewater and coastal waters include ibuprofen and other painkillers, anti-depressants, steroids, caffeine, estrogens and other hormone-containing products, anti-epileptics, cancer drugs, antimicrobials such as triclosan, and antibiotics [274,275,276,277]. Many pharmaceutical and cosmetic products in current use contain manufactured plastic nanoparticles [278].

Some PPCPs have potential to accumulate in fish and shellfish species consumed by humans and thus have potential to affect human health [279]. Concern is growing that pharmaceutical chemicals and their metabolites can damage marine species through a range of toxicological mechanisms, including endocrine disruption and neurotoxicity. A recent case study suggests that the widely used sunscreen chemical, oxybenzone (benzophenone-3) may have toxic effects on the larval forms of several coral species [280]. The study reports that these effects include transformation of coral larvae from a motile state to a deformed, sessile condition; increased coral bleaching; leading to deformed skeleton formation; and DNA lesions.

#### Hazards of Combined Exposures to Multiple Chemical Pollutants

Manufactured chemicals are rarely present in the environment in isolation, but instead are found in complex mixtures. This complicates assessment of health impacts, because toxicological tests most often are conducted on one chemical at a time, thus potentially missing additive, antagonistic, or synergistic actions that could result from simultaneous exposures to mixtures of POPs and other manufactured chemicals that occur together in the oceans as “chemical cocktails” [281,282]. Future public health studies should pay additional attention to complex mixtures and cumulative risk assessment. The possibility of interaction among multiple POPs raises the question as to whether any one chemical that shows an association with disease is really acting a “proxy” for the combined effect of all the chemicals [283,284].

Consideration of the susceptibility of exposed populations is also important. The safe limit for exposure at sensitive life stages of development, *in utero* or in nursing infants, will be lower than for adults. And in the adult population, underlying disease may modify risk. Finally, “safe” levels for one pollutant may not pertain to the combined risk from simultaneous exposure to the many pollutants to which a person may be exposed.

#### Balancing Risks and Benefits of Exposure to Chemical Pollutants in the Oceans

Because of widespread pollution of the oceans by toxic metals and POPs and contamination by HAB toxins (discussed in the next section of this report), it is necessary to balance the risks of chemical pollutants in seafood against the benefits derived from nutrients unique to fish and shellfish. Thus, the benefits of essential fatty acids (EPA and DHA) in farmed and wild fish must be balanced against the risks for adverse health outcomes from chemical contaminants in those same fish [285,286].

To assess whether the beneficial effects of omega-3 fatty acids in seafood may mitigate the adverse effects of methylmercury on brain development, IQ was measured in 282 school-age Inuit children in Arctic Québec whose umbilical cord blood samples had been analysed for mercury and DHA [287,288]. The investigators found that prenatal mercury exposure was associated with lower IQ after adjustment for potential confounding variables. Incorporation of DHA into the model significantly strengthened the association with mercury, supporting the hypothesis that the beneficial effects of DHA intake can at least partially offset the harmful effects of mercury [65].

Similarly, some studies have noted that the beneficial effect of fish consumption on the cardiovascular system appears to be reduced by co-exposure to PCBs [289]. The risk differential between wild and farmed salmon is a prime example of these concerns. While the abundance of omega-3 as well as omega-6 fatty acids differ between wild and farmed fish, both contain high levels of these beneficial compounds. However, farmed fish tend to have higher levels of PCBs and other contaminants than wild fish, and contaminant burdens differ between fish farmed in different parts of world. Determining risk of those contaminants depends in part on which outcome is considered, and whether the risk is from one or many chemicals.

Studies comparing relative risk of cancer and other health outcomes associated with dioxin-like compounds in salmon concluded that consumption of farmed salmon would need to be limited to many fewer meals per month than for wild salmon, to reduce cancer risk to a level near the WHO “tolerable daily intake” for dioxin-like compounds [290,291].

A review examining the health risks and benefits of seafood consumption and the impact of fish consumption on sustainability of fish stocks concluded that “few, if any, fish consumption patterns optimize all domains”, but called for development of “comprehensive advice … to describe the multiple impacts of fish consumption” [292]. Several groups have disseminated such guidance [293,294,295].

### Chemical Pollutants in the Oceans and the Global Burden of Disease

Despite extensive knowledge of the toxicology of many ocean pollutants, the contribution of chemical pollutants in the marine environment to the global burden of disease (GBD) is, with the exception of mercury [296,297], largely unknown. A major impediment to developing these estimates is that detailed, population-level studies of human exposures to ocean pollutants have not been conducted, although it is unarguable that fish and other seafood are a major source of human exposure. Moreover, POPs and other toxic chemicals that are found in terrestrial meat sources can in fact originate in the oceans, because fish meal, containing POPs, is often used in animal feeds [298].

### Oil Spills

Crude oil and petroleum products are complex mixtures of light and heavy hydrocarbons, toxic metals, and other chemicals. Polycyclic aromatic hydrocarbons (PAHs) are a particularly hazardous component. When oil spills and leaks release these toxic chemicals into the marine environment, they can bioaccumulate in the food web; kill fish, birds and marine mammals; destroy commercial fisheries, aquaculture operations, and shellfish beds; release toxic volatile toxic chemicals such as benzene to the atmosphere; and foul shorelines.

Oil spills range in magnitude and visibility from massive releases such as the Deepwater Horizon disaster in the Gulf of Mexico or the Amoco *Cadiz* Oil Spill off the coast of France down to chronic, slow leaks from pipelines and aging tankers. Petroleum in the marine environment can be either fresh or highly weathered, meaning that it has undergone a variety of chemical and photochemical processes that change its composition and toxicity.

**Figure 8:**
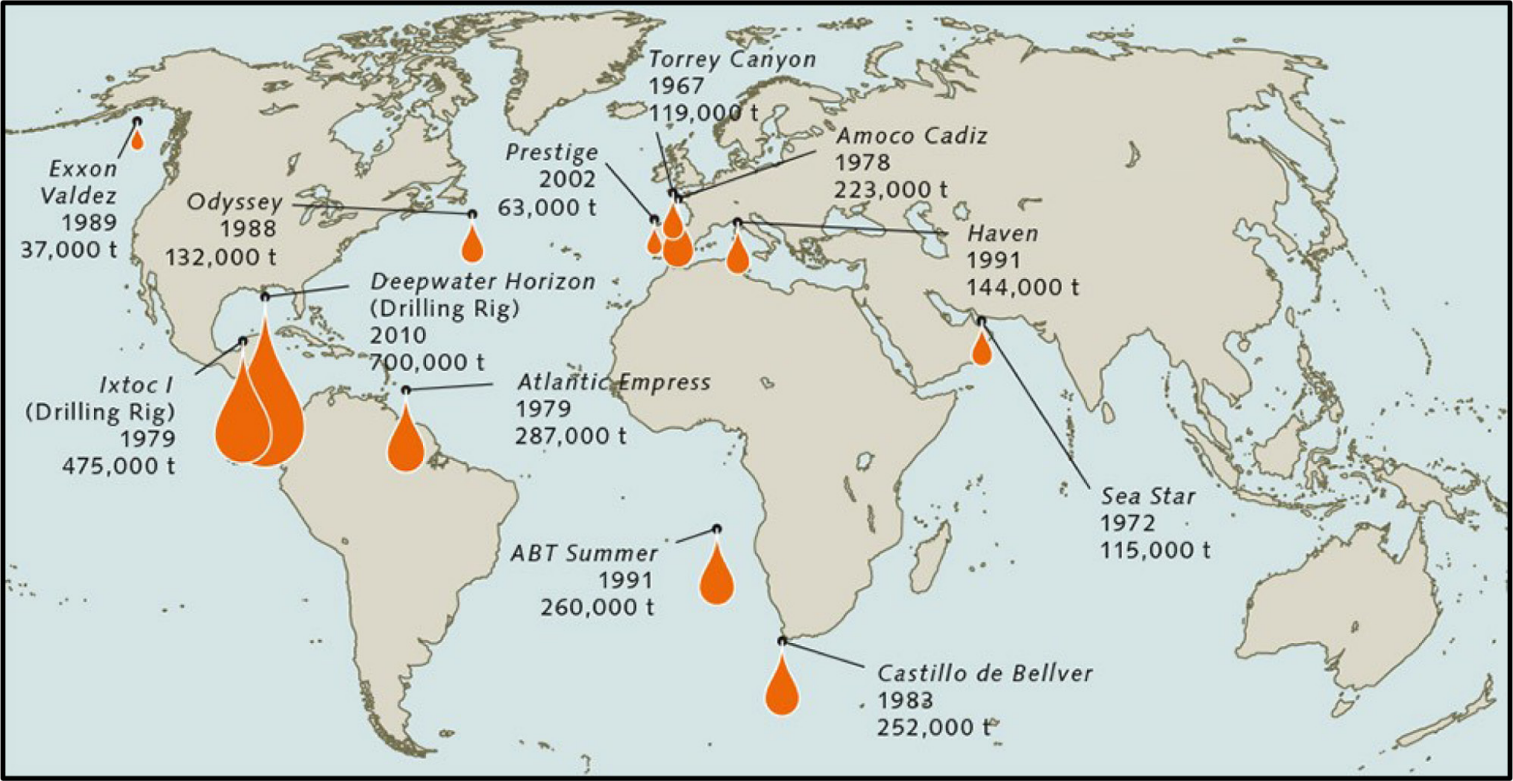
Major Oil Spills, 1967–2010. From: World Ocean Review 3, maribus gGmbH, Hamburg 2015. *Source*: Bücker et al. 2014 [314]. See also ITOPF 2019 [315].

Oil spills have occurred with increasing frequency in recent years as the result of growing global demand for petroleum. These spills have resulted in direct release of millions of tons of crude oil and other petroleum products into the oceans (Table 1, Figure 8).

Ecosystem effects of oil spills include disruption of food sources, destruction of fragile habitats such as estuaries and coral reefs, and fouling of beaches [300]. Marine and coastal wildlife, including birds and mammals, can be exposed to petroleum-based pollutants through ingestion, absorption, and inhalation. Ingestion of these materials can lead to digestive problems, ulcers, and bleeding; kidney and liver damage; reproductive failure; and anemia. Inhalation can lead to lung problems [301] that appear to persist long after initial exposures [302]. Effects on immune systems of fish predispose them to infections [303]. PAHs contained in oil spills have been shown to cause DNA damage in marine species and have been associated with hepatic, pulmonary and cardiac lesions in Arctic seals [304,305,306,307].

Human health and well-being also can be seriously affected by oil spills. Heaviest exposures and the most severe health consequences occur among occupationally exposed populations such as oil industry workers and workers involved in cleanup efforts. Cohort studies suggest that respiratory effects may persist for 2+ years post spill in some responders [308]. DNA damage has been documented in cleanup workers [309,310]. Community residents can be exposed through consumption of contaminated seafood and inhalation of volatile petrochemicals. Some studies have suggested little long-term health risk for consumption of fish or shellfish after the Deep Water Horizon spill. However, assessments of the possible health hazards of abundant alkylated PAHs have not been included in such studies [311].

In addition to their effects on physical health, major oil spills, like other disasters, can have serious impacts on mental health. Populations in areas with lower income are often at heightened vulnerability to such effects [312]. There is need for cohort studies on resilience to disasters as well as on chemical stressors [312,313].

### Biological Contamination of the Oceans

Many toxin-producing algae, pathogenic bacteria, viruses, fungi, and protozoa are native to marine and estuarine environments. Other species can be introduced to the oceans as the result of human activity.

### Marine Algae and Harmful Algal Blooms (HABs)

Algae, microscopic and macroscopic, are the foundation of the aquatic food web. They are the invaluable primary producers of fixed carbon, a vital nutrient that supports aquatic ecosystems, and of oxygen. Free-living planktonic algal species dominate the world’s oceans, and a small number of species account for the great majority of the global algal biomass. In coastal and estuarine systems, cyanobacteria, as well as dinoflagellates, diatoms, and cryptophytes emerge seasonally and are vital components of these ecosystems. Floating tropical beds of brown macroalgae (e.g., *Sargassum*) serve as habitats and nurseries for many marine species. They also sequester CO_2_ and thus mitigate global warming and ocean acidification [316,317].

Marine microalgae are of great importance to human health and well-being not only because they support the marine food web upon which all commercial fisheries depend, but also because they provide food for aquaculture, produce a range of pharmaceutical compounds [14], and are potentially a source of renewable biofuels [318].

**Figure 9:**
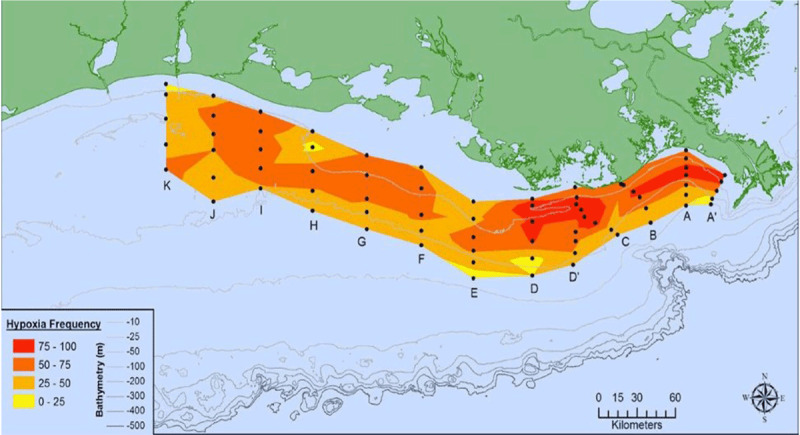
Frequency of Bottom-Water Hypoxia (‘Dead Zones’), Gulf of Mexico, 1985–2014. *Source*: Rabalais et al., 2019, CC BY 4.0 [327].

On the negative side, some algal species are noxious [319] and produce powerful toxins have potential to cause great harm [320]. When high densities of these species accumulate in an area of the ocean, they can form harmful algal blooms (HABs) – described as “red tides”, “green tides”, or “brown tides”. In these blooms, the great masses of algae that have accumulated in an area of the sea exhaust inorganic nutrients in the water column allowing bacteria move in and decompose the senescing organic material. The consequences are reduced dissolved oxygen in the ocean, dead zones, fish kills, and a broad range of adverse ecological impacts [321,322,323] (Figure 9).

HABs directly harm human health by producing toxins, potent natural compounds that can cause disease and death, most commonly through consumption of contaminated seafood [32,323,324,325,326].

#### Causes and Drivers of HAB Events

HABs are not a new phenomenon and some occur naturally. However, the frequency and magnitude of HAB events appears to be increasing [328]. These increases have been linked to three factors:

1. Increasing pollution of the oceans, and especially of coastal waters by nitrogen and phosphorus which leads to eutrophication. Sources of nitrogen include agricultural runoff, septic tank leachate and effluent from municipal deep injection wells [329,330,331];
2. Sea surface warming; and
3. Ocean acidification.

Increases in frequency and severity of HAB events have been linked to increasing coastal pollution in the Seto Inland Sea of Japan in the mid-1970s [332] and in the northwestern Black Sea in the 1970s and 1980s [333]. Both of these situations have subsequently been remediated, and case studies describing these and other successful remediation efforts are presented in the section of this report on Successes in Prevention and Control of Ocean Pollution [334].

A current example of the effect of increasing coastal pollution on HAB frequency is seen at the mouth of the Changjiang River in China, where nitrate concentrations have increased four-fold in the past 40 years and phosphate concentrations have increased by 30%. The main drivers are increases in population size and agricultural production. Significant increases in algal biomass and a change in the composition of the phytoplankton community have resulted. The frequency of local HABs has increased dramatically [335].

#### Climate Change and HABs

**Figure 10:**
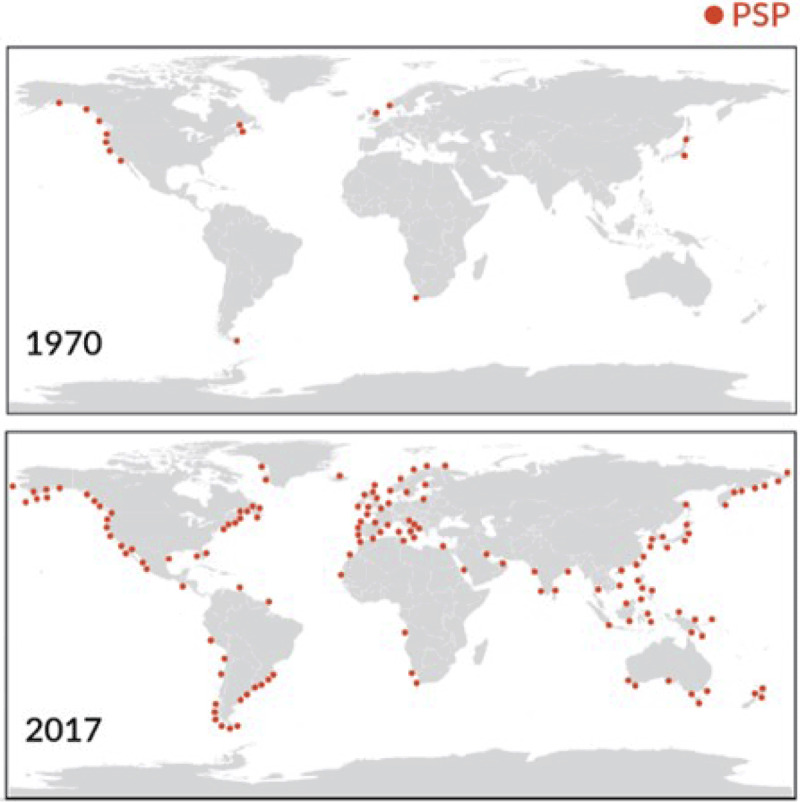
Geographical Distribution of Paralytic Shellfish Poisoning (PSP) Events, 1970 and 2017. *Source*: US National Office for HABs, Woods Hole, MA.

Increases in the frequency and severity of HABs have been linked to changing weather patterns such as major warming events, increased runoff, and changes in ocean currents (Figure 10). Examples include recent *Alexandrium* blooms in the northeastern United States [336] and massive blooms of *Pseudonitzschia* on the US west coast associated with a mesoscale warm-water anomaly termed “the blob” [337]. These events presage projected future climate scenarios [54,338,339].

Sea surface warming leads to range extensions of HAB species and to the appearance of algal toxins in previously unaffected areas [53,55,340,341,342,342]. An example is seen in the recent, first ever detection of HAB toxins in Arctic waters [343]. The movement of harmful algae into the Arctic coupled with northern indigenous peoples’ lack of experience with HAB toxins put these populations at high risk of exposure and disease. This risk is compounded by lack of knowledge about uptake of HAB toxins by species such as whales, walruses, seals, and seabirds used by northern indigenous people as food sources.

Another example of climate-driven change in HAB range that has already occurred is poleward extension in the geographic ranges of the benthic dinoflagellates responsible for ciguatera poisoning into warm-temperate habitats, for example from the Caribbean Sea northward into the Gulf of Mexico [55,342,344]. This range extension appears to be associated with warming sea surface temperatures and higher storm frequencies, and destruction of coral reefs [345,346,347,348,349]. It is reflected in increased numbers of calls about ciguatera poisoning to poison control centers in the United States.

An impact on HAB biology that appears to reflect synergy between global climate change and ocean acidification is the observation that HAB toxins can become more potent at higher temperatures or under more acidic conditions [350,351]. This change may reflect temperature-induced shifts in the relative abundance of dinoflagellate species [340,352,353].

#### Pathways of Human Exposure to HAB Toxins

Consumption of fish and shellfish that have ingested toxic algae is a major route of human exposure to HAB toxins. Filter-feeding shellfish such as oysters and mussels pose an especially high risk because these species ingest toxic algae and then accumulate algal toxins to high concentrations that can cause acute disease and sudden death in shellfish eaters. The poisoning syndromes caused by HABs in shellfish include paralytic, neurotoxic, amnesic, diarrhetic, and other gastrointestinal poisoning [354,355]. Consumption of finfish and shellfish containing ciguatera toxin may also result in ciguatera poisoning.

Human exposure to HAB toxins can also occur through skin or respiratory contact via swimming or visiting beaches during algal blooms. People have reported skin rashes, respiratory irritation such as sneezing, and a burning or itching in the nose or throat while swimming, visiting, or working at the beach during *Karenia brevis* red tide events [356,357]. People with asthma appear to be at particular risk [358]. *Karenia brevis* blooms are associated additionally with increases in emergency room admissions for respiratory, gastrointestinal, and neurologic illnesses [359,360,361]. There is evidence that people experience adverse effects also during *Sargassum* blooms [362] and from exposures to algal-derived palytoxins [363].

Macroalgal blooms, can harm human health by causing massive accumulations of algae in bays and on beaches. When these piles of algae decompose, they can release foul-smelling and hazardous gases, including hydrogen sulfide, methyl mercaptans, and dimethyl sulfide [364]. Coastal populations exposed to decomposing algal mats have reported eye and respiratory tract irritation.

#### Syndromes Associated with HAB Toxins

HABs cause a variety of human diseases, some of them extremely serious (**Text Box 3**). HAB-related illnesses are for the most part acute, and acute reference doses (ARfD) have been derived to protect the public against these acute exposure events (See Appendix Table 2 in the Supplementary Appendix). Little research has been done to evaluate chronic illness after either acute or chronic exposures to HAB toxins, and information on long-term health effects is still insufficient to allow determination of tolerable long-term daily intakes (EFSA opinions or FAO/WHO/IOC ad hoc expert consultation).

**TEXT BOX 2: *Chemical Pollution of the Oceans and Reduced Generation of Oxygen*.**

A novel mechanism by which petrochemical pollutants in the oceans may endanger human and ecosystem health is through reducing production of oxygen [197]. Beneficial marine microorganisms such as cyanobacteria of the genus *Prochlorococcus* are major producers of oxygen. Through photosynthesis, the billions of these organisms in the earth’s oceans remove CO_2_ from the atmosphere and convert it to oxygen.

Recent experimental findings from the Atlantic, Pacific, and Indian Oceans have found that mixtures of POPs and aromatic hydrocarbons in seawater at concentrations only two times above usual background levels can reduce expression of photosynthetic genes in *Prochlorococcus* and thus impede oxygen generation [6,198]. The photosynthetic toxicity of pollutant mixtures exceeds that of single chemicals by as much as three orders of magnitude [5].

**TEXT BOX 3: A Primer on Poisonings by HAB Toxins.**

Consumption of contaminated seafood is the major route of human exposure to HAB toxins. Many thousands of poisoning episodes occur worldwide each year.

***Paralytic Shellfish Poisoning (PSP)*** is caused by saxitoxins (STX), potent neurotoxins that act on voltage-gated sodium channels as well on other nervous system receptors [366,367]. PSP typically begins with tingling sensations or numbness of face, neck, fingers, and toes. These symptoms progress within 30 minutes to weakness, limb incoordination, and respiratory difficulty. In severe cases, respiratory paralysis, cardiovascular shock, and death may ensue. There is no antidote to PSP, and the only available treatment consists of artificial respiration by ventilator [368,369] and removal of non-absorbed toxins from the gut with activated charcoal. STX is listed as a Schedule 1 chemical intoxicant by the Organization for the Prohibition of Chemical Weapons (OPCW) [370]. The lethal oral dose is 1–4 mg [371].

***Amnesic shellfish poisoning (ASP)*** is caused by **domoic acid (DA)**, a potent toxin produced by planktonic diatoms that targets glutamate receptors in the central nervous system [372,373]. After initial gastrointestinal symptoms, affected persons develop confusion, lethargy, disorientation, and short-term memory loss. Severe cases evolve to coma. Deaths have occurred [368,369]. A persistent toxicity syndrome has been defined consisting of episodic seizures and permanent loss of spatial memory [374].

***Diarrhetic shellfish poisoning (DSP)*** is associated with exposures to okadaic acid and dinophysis toxins. The syndrome presents with diarrhea, nausea, vomiting and abdominal pain. Symptoms may be confused with infectious intestinal diseases. No lethal cases have been reported [368,369].

The **azaspiracid group of HAB toxins** also results in diarrhetic symptoms. Its mechanism of action is not yet known, but recent evidence suggests that mitochondrial dehydrogenase may be a major target of this toxin group [375].

The **yessotoxins** are a group of lipophilic HAB toxins. Although never associated with human illness, they are controlled in seafood based on an acute reference dose established through oral administration of yessotxins in toxicological studies in experimental animals.

***Neurotoxic shellfish poisoning (NSP)*** is caused by brevetoxins (BTX), neurotoxins that target voltage-gated sodium channels and cause depolarization of neuronal, muscular and cardiac cells [376]. NSP produces a mixture of gastrointestinal and neurologic symptoms – nausea, vomiting, diarrhea, and abdominal cramps as well as paresthesia, paralysis, convulsions, and coma [377]. Symptoms begin within 30 minutes to three hours following consumption of contaminated seafood.

***Ciguatera Fish Poisoning (CFP)*** is caused by consumption of fish and shellfish that have accumulated ciguatoxins (CTX) in their tissues [378,379,380]. CTXs are neurotoxins that target voltage-gated sodium channels. They are produced by benthic dinoflagellate plankton of the genera *Gambierdiscus* and *Fukuyoa* that live on coral surfaces and also by bottom-dwelling algae.

CFP is associated with higher sea surface temperatures and the El Nino Southern Oscillation. In the United States, the number of CFP-related calls to poison control centers appears to correlate with warmer sea surface temperatures and higher storm frequencies.

CFP is estimated to affect 50,000 to 200,000 people per year. It is the most commonly reported of the HAB-associated illnesses globally. It an important health problem in the Caribbean and Pacific regions and more recently has been reported in the Mediterranean.

Symptoms of CFP include gastrointestinal distress that may occur before or simultaneously with peripheral neurological symptoms, neuropsychiatric, and cardiovascular symptoms [381]. Symptoms generally appear within 12 hours after eating contaminated seafood [382,383]. Although rarely fatal, CFP symptoms have been reported to persist in about 20% of cases, lasting days, months or even years, with worsening symptoms of anxiety or depression [381,384].

**Clupeotoxism** is a form of HAB-related human poisoning caused by consumption of contaminated fish and crustaceans contaminated by palytoxin (PTX) [385]. Exposure can also occur through handling zoanthid corals in either private homes or aquarium shops [386]. Symptoms include gastrointestinal, neurological, and cardiovascular symptoms, as well as weakness, cough, and muscle pain.

Children may be more likely than adults to be affected by HAB toxins due to a combination of greater exposure, riskier behaviors, and sensitive developmental stage. Children also consume more food per unit body weight than do adults and thus may receive higher relative doses [365].

#### Prevention of HABs

The frequency and severity of some HAB events can be controlled by reducing releases of nitrogen, phosphorus, animal wastes, and human sewage into coastal waters. (See **Text Boxes 9–13**). Additional actions that can be taken to mitigate HABs are the following:

- Increase freshwater flows and tidal exchanges in coastal waters to increase flushing, prevent stagnation, and enhance the composition of coastal phytoplankton communities. In some instances, this will require modifying built structures such as breakwaters, jetties, and dams that impede flow of fresh and salt water [387] (See **Text Box 4**).
- Restrict activities that might result in the accidental transfer of harmful algal species into environments where they do not naturally occur (e.g., ballast water discharge) [388,389].

**TEXT BOX 4: Reduced Water Flow and Increased Frequency of HABs.**

An example of an area where changes in freshwater flow may be affecting HAB incidence is in the Bohai Sea of China. The Bohai is one of several regions in China where the number of HABs has increased in recent years. Due to droughts and water diversions for drinking water and agriculture, several of the rivers that used to flow freely into the Bohai are now dry for many days every year. This reduces the dilution of pollution loads in nearshore waters and also reduces stratification.

Dams are another factor that can increase frequency of HABs by altering fresh water flow into the ocean. Dams decrease turbidity and the availability of silicate to downstream waters due to sediment trapping within impounded waters. A decrease in the amount of silicate reaching coastal waters, concurrent with increases in water transparency can lead to shifts in the nutrient ratios that regulate phytoplankton community composition [390]. An increase in HAB frequency has been observed downstream of the massive Three Gorges Dam in China, and this increase is linked to a decrease in sedimentation and turbidity [391].

#### Prevention of HAB Poisoning

Routine monitoring for HAB toxins in shellfish is key to the prevention of human illness caused by these toxins. Monitoring programs are typically embedded within comprehensive shellfish safety programs. Details are presented in the Monitoring of Ocean Pollution section of this report.

Another strategy for mitigating the impact of HAB toxins on human health is to process harvested shellfish in such a way as to reduce toxicity to an acceptable level. An example is the removal of scallop viscera and marketing of only the adductor muscle, which generally contains little or no HAB toxins [389].

#### Economic and Social Consequences of HAB Poisoning

HABs have multiple negative economic and social effects. In the US, it is conservatively estimated that the average annual cost of marine HABs is USD $95million [392]. Health impacts are responsible for the largest component of these economic loses [331]. Economic losses attributable to HABs are estimated to $850 million (USD) annually in Europe and over $1 billion (USD) in Asia [392]. The costs of individual catastrophic HAB events can be overwhelming. Mexico, for example, spent $17 million in 2018 to remove 500,000 tons of *Sargassum* from its Caribbean beaches and declared a state of emergency. Another large HAB resulted in the largest fish farm mortality ever recorded and a loss of USD $800 million [339]. Increased frequency of respiratory ailments, aerosolized toxins, noxious gas, dead fish, proliferation of biting sand fleas from decaying piles of macroalgae, and discolored waters drive tourists away from beaches, change recreational habits, and thus reduce income from tourism in coastal communities [393,394,395,396].

### Ocean Bacteria, Viruses, and Protozoa

Bacteria are abundant in the oceans. Every cubic centimeter of seawater contains, on average, one million microbial cells and the global ocean harbor an estimated 4–6 × 10^30^ microbial cells [397]. Although the majority of bacteria in the oceans are harmless to humans, some are pathogenic. Naturally occurring marine pathogens of great significance for human health include *Vibrio cholerae, Vibrio vulnificus, Vibrio parahaemolyticus*, and *Mycobacterium marinum*.

With climate change, sea surface warming, and worsening marine pollution, the geographic ranges of naturally occurring marine pathogens as well as of microorganisms introduced to the oceans from land-based sources are expanding. Harmful bacteria are moving into estuaries, bays, and regions of the oceans they did not previously inhabit and moving poleward into cold, previously uncontaminated waters [22].

Microbial infections are contributing to degradation of fragile marine environments such as coral reefs [398,399]. They contribute to shellfish mortality in both wild and farmed areas, thereby affecting economies [400,401]. Widening geographic ranges of human diseases caused by marine microorganisms and the appearance of disease in previously unaffected populations are additional consequences [402].

#### Marine *Vibrio* Species and Human Disease

Marine bacteria of the genus *Vibrio* are particularly important causes of disease and death [403]. *Vibrio cholerae*, the causative agent of cholera, is the species of greatest concern. *Vibrio* species exhibit strong seasonality, and warmer water temperatures result in increased concentrations in estuarine and coastal waters [50,51,404,405,406,407,408]. Further warming of coastal waters caused by climate change is likely to further increase abundance of *Vibrio* bacteria and expand their geographic range [409]. These changes will likely result in increased frequency of *Vibrio* infections in coming decades and possibly to appearance of *Vibrio* infections in previously unaffected areas [52]. There is some indication that after extreme weather events such as hurricanes, droughts, and tropical storms shifts occur in the composition of *Vibrio* species and that these shifts are driven by discharges of sewage and inorganic nutrients into coastal waters [410].

**Figure 11:**
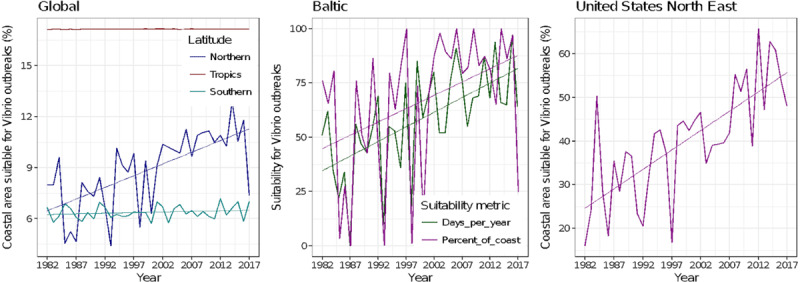
Trends in conditions favorable to *Vibrio* outbreaks in selected world regions [411]. *Source*: Reprinted from Watts et al. The 2018 report of the Lancet Countdown on health and climate change: shaping the health of nations for centuries to come. *Lancet* 392: 2479–2514, 2018, with permission from Elsevier.

**Figure 12:**
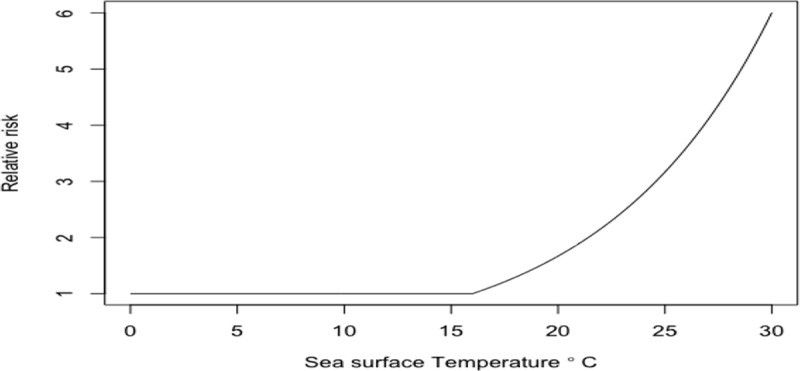
Sea surface temperature and relative risk of clinically notified cases of *Vibrio* infection, Sweden, 2006–2014 [416]. *Source*: Semenza et al. (2017), https://doi.org/10.1289/EHP2198.

*Vibrio parahaemolyticus* and *Vibrio vulnificus* are two additional *Vibrio* species that pose grave risks to human health [412,413]. These organisms are now appearing for the first time in previously cold waters at northern latitudes with major peaks occurring during warm summers (Figure 11) [411]. This trend is particularly well documented for the Baltic Sea, where the annual incidence of *Vibrio* infections is reported to almost double for every one-degree increase in sea surface temperature (Figure 12) [402,414]. Similar trends have been reported in the United States where incidence of infections by *Vibrio* species has increased by 115% in the past decade, especially along the Gulf, Northeast, and Pacific Northwest coasts [50,414,415].

*Vibrio vulnificus* can enter the human body either through ingestion of contaminated seafood or through open wounds [417]. When *V. vulnificus*, known colloquially as ‘flesh-eating bacteria’, enters an open wound it can cause severe infections such as necrotizing fasciitis (**Text Box 5**).

**TEXT BOX 5: Case Studies of *Vibrio* Wound Infection.**

*Vibrio* wound infections are generally rare, even though the bacteria are quite common in brackish, mesohaline estuarine systems [424]. Unfortunately, these infections can be very severe resulting in some cases in amputation of infected limbs and loss of life. The great majority occur in males, especially in men over 40 years of age, presumably reflecting occupational and recreational activities [425,426].

Case study. In 2011, a report was presented of three elderly men in New Caledonia who developed severe gastrointestinal illness after consumption of raw oysters during a period of particularly heavy rainfall, and regional flooding. *V. vulnificus* was confirmed as the causative agent through PCR amplification of the hemolysin gene.

Case study. In 2005, 18 cases of confirmed wound infections with *V. vulnificus* and *V. parahaemolyticus* were observed following Hurricane Katrina. Five of the patients died [427].

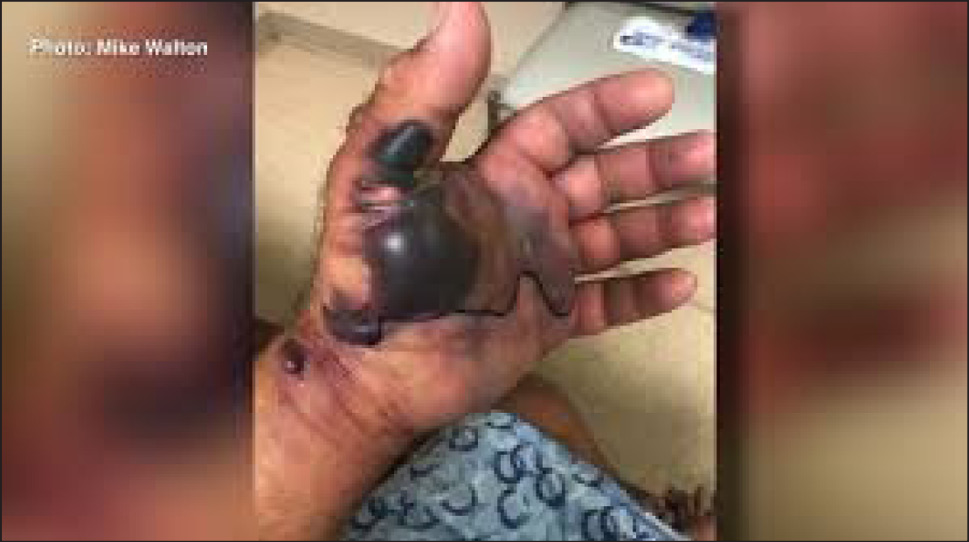

Case study. Next-generation sequencing (NGS) was used to diagnose *V. vulnificus* infection in a 55-year old man who was admitted to a hospital in Wenzhou, China hospital with severe wound infection. The man had been selecting fish at the market at 6:00 AM and developed a skin infection on his hand. The infection progressed rapidly, and the patient was admitted to hospital 11 hours later. Even though blister fluids, and wound and blood samples returned negative results by bacterial culture, tissue analyses using NGS were able to confirm *Vibrio* infection and guide treatment. After two weeks of hospitalization, the man was released.

These cases and other published literature on the emergence of pathogenic forms of *Vibrio* following flooding and tropical events indicate the need for improved warning systems in anticipation of the increased frequency of extreme weather events that is expected to accompany climate change [428,429,430].

Ingestion of shellfish contaminated by *V. vulnificus*, especially oysters, causes more than 90% of cases of *V. vulnificus* gastroenteritis [418,419]. This reflects the fact that filter-feeding shellfish such as oysters, clams, and mussels can concentrate *Vibrio* by several orders of magnitude over concentrations in seawater [412,418].

*Vibrio vulnificus* gastroenteritis can progress very rapidly to septicemia – sometimes within 24 hours after ingestion of contaminated seafood [418,420]. Even with aggressive medical treatment, the case-fatality ratio for *Vibrio vulnificus* septicemia is greater than 50%. *Vibrio vulnificus* thus has the unlovely distinction of having the highest case-fatality ratio of any foodborne pathogen [418,420]. It is the cause of 95% of seafood-borne deaths in the USA [420].

Recent data suggest that rising sea surface temperature may expand not only the temporal and spatial distribution of *Vibrio* species, but also increase the virulence and antimicrobial resistance of some *Vibrio* strains [421,422,423].

**Figure 13:**
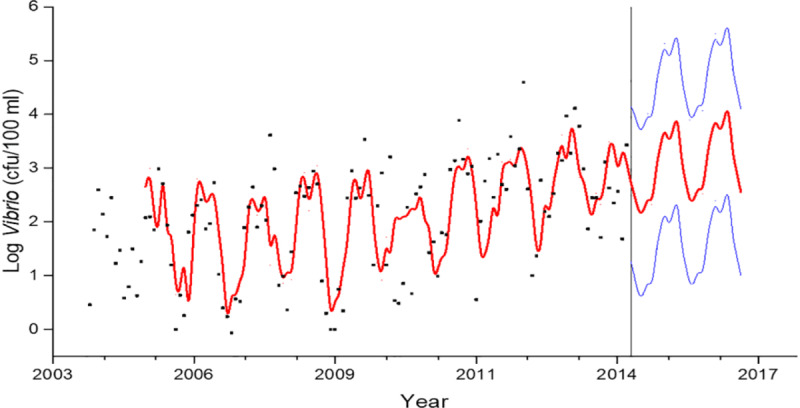
Seasonal abundance of *Vibrio* species, Neuse River Estuary, NC, USA, 2003–2017. (Autoregressive integrated moving average of mean monthly abundance at a mid-water station). Dots are actual measurements. Red line represents model abundance. Blue lines are 95% confidence intervals. *Source*: Froelich et al. (2019), https://doi.org/10.1371/journal.pone.0215254, Creative Commons, license CC BY 4.0.

Salinity is another factor that affects the abundance of *Vibrio* species in marine environments. Typically, *V. vulnificus* and *V. parahaemolyticus* are not prevalent in waters where salinity exceeds 25 parts per thousand. Recent anecdotal reports from the UK, EU, and Brazil indicate, however, that shifts in the composition of *Vibrio* communities in estuarine systems and increases in *Vibrio* infections are now being recorded in waters where salinity is greater than 30 parts per thousand [431], possibly reflecting an interaction between salinity and sea surface warming. A decade-long study of *Vibrio* conducted in the Neuse River Estuary in North Carolina, USA, has shown the temperature is not increasing in that system, and that temperature increase cannot therefore explain the significant increase observed in *Vibrio* concentrations (Figure 13) [424].

In some major river basins (i.e., the Amazon, the Ganges, the Brahmaputra, and the Congo), increased incidence of *Vibrio* infection is reported to coincide with high sea surface temperatures and high discharge events, events that typically are associated with abnormal phytoplankton growth [432]. In other marine coastal areas, the global abundance of *Vibrio* has been shown to correlate with chlorophyll, acidity, maximum sea surface temperature, and salinity [50].

#### Allochthonous Bacterial Pathogens in Marine Environments

Allochthonous bacteria are microorganisms not native to marine environments that are introduced into coastal waters from land-based sources. Allochthonous pathogens of greatest concern include virulent *Enterococcus* species, *Escherichia coli* serotypes (e.g., O157:H7), *Campylobacter* species, *Clostridium* species, *Shigella* species, and *Salmonella* species [433].

Pathogenic bacteria can enter coastal waters through sewage effluent, agriculture and storm water runoff and wastewater discharges from ships [434]. Rivers, especially those near major population centers, are an important source [434]. Through horizontal gene transfer, allochthonous bacteria can introduce harmful new genetic traits into indigenous marine microorganisms thus increasing their virulence and their capacity for anti-microbial resistance [435].

Climate change is accelerating the introduction, dispersion, and growth of allochthonous bacteria in coastal waters. For example, rising sea surface temperatures have been shown to increase the abundance of *Salmonella* species in Hawaiian coastal streams [436]. Warming may also increase the variability of salinity gradients along coastlines [437] thus affecting the growth and persistence of fecal-oral pathogens and increasing risk for major outbreaks of diarrheal disease [438].

Fecal-derived bacteria in marine environments tend to bind to particle surfaces (sediment, sand, plastics) where they form biofilms that enhance their survival. In estuarine environments, for example, the concentration of fecal bacteria is generally one or more orders of magnitude higher in surface sediments (per 100 g dry weight) than in the water column (100 ml). The survival of fecal bacteria in the oceans is thus positively linked to concentrations of pollutants and other suspended matter in the water column [439,440,441].

#### Human Diseases Caused by Allochthonous Bacterial Pathogens

Bacterial pathogens in the marine environment are responsible for a wide range of acute and chronic diseases. These include diarrhea and gastroenteritis, ocular and respiratory infections, hepatitis, and wound infection. Transmission of disease occurs mainly through ingestion of contaminated water and consumption of contaminated seafood [433].

From 1973 to 2006, 188 outbreaks of seafood-associated infections causing 4,020 illnesses were reported to the Foodborne Disease Outbreak Surveillance System in the United States [442]. Most of these outbreaks were due to bacterial agents (76.1%), a significant proportion of them linked to pathogens with a human reservoir such as *Salmonella* and *Shigella* [443,444] (Table 2).

**Table 2:** Optimal Temperature and Salinity Fecal-Oral Pathogens in Sea-Water [445].

Pathogen	Related Diseases	Salinity (ppt)	Temp (°C)	Notes

*Vibrio* spp	Vibriosis	5–25	15–30	*Vibrio* species naturally thrive in warm waters with moderate salinity
*Campylobacter jejuni*	Campylobacteriosis	0–0.5	30–45	
*Shigella*	Shigellosis	0–20	4–37	Frequent outbreaks in US
*E coli* O157:H7	Bloody diarrhea	0–34	4–37	Frequent outbreaks in US
*Legionella* sp	Legionnaire’s Disease	0–0.5	25–47	High incidence in US Typically found in freshwater, but can also survive in marine environments

#### Antimicrobial Resistance in Coastal and Ocean Environments

Antimicrobial resistance (AMR) is likely to have been present for millions or billions of years in marine microbial communities as the result of resistance mechanisms that bacteria have evolved in response to naturally occurring threats [446].

More recently, however, the prevalence of AMR has been increasing in marine environments, especially in coastal waters. These increases appear to reflect increasing introductions from land-based sources of allochthonous bacteria that carry resistance genes that can be passed to marine bacteria through horizontal gene transfer [16,447]. Such exchanges may account for the acquisition of AMR by indigenous pathogens such as *Vibrio*.

The development of confined animal feeding operations (CAFOs) to enhance livestock production and increase the profits in the poultry, beef, and swine industries have further promoted the development of AMR bacteria. These facilities are associated with poor waste treatment practices, and the vast quantities of effluent they release into waterways and directly into the ocean are associated with increased genetic encounters across “promiscuous” bacterial species able to transfer resistance genes horizontally.

An increasing body of evidence documents that significant human exposure to AMR bacteria can occur in coastal environments. A study in the UK reports that an estimated 6 million exposures occur per year to cefotaxime-resistant *E. coli* [448]. Another study found an increased probability of gut colonization by cefotaxime-resistant *E. coli*, a known risk factor for infection, in persons such as swimmers and surfers heavily exposed to contaminated recreational waters [449]. Recent studies of near-bottom waters from the Polish coastal zone reported multiple antibiotics at ng/L concentrations, with enrofloxacin reported at >200 ng/L [450,451].

#### Marine Viral Pathogens and Human Health

Viruses in coastal and estuarine systems that pose serious threats to human health include the *Picornaviridae* (enteroviruses, e.g., poliovirus, coxsackievirus, and echovirus), *Adenoviridae* (adenovirus), *Astroviridae* (astrovirus), *Reoviridae* (reovirus, rotavirus) and most significantly the *Caliciviridae*, a genus that includes norovirus and calicivirus [452]. Norovirus infections represented 21% of enteric virus infections reported from recreational water exposures across the USA from 2000–2014 [453]. Noroviruses enter coastal waters through stormwater, flooding, illicit boat discharges, and sewage system leaks and spills (E.g., **Text Box 6**).

**TEXT BOX 6: Case Studies of Gastrointestinal Illness among Swimmers and Surfers Caused by Viruses in Polluted Marine Environments.**

A recent study of gastrointestinal infections among surfers on the beaches near San Diego, California, USA, found that during rainy weather there was increased abundance of norovirus contamination in storm water runoff along the beaches [454]. Rates of gastrointestinal illness were increased among surfers during these periods of high contamination [455].

Other studies of gastrointestinal illness among swimmers during periods of heavy storm water discharge to coastal environments have found strong relationships between disease incidence and proximity to storm water pipes [36,37].

Dramatic improvements have been made in the past decade in diagnostic technologies for direct quantification of viral pathogens in marine environmental samples. These include new molecular approaches such as digital droplet PCR [454].

#### Marine Parasites and Human Health

Parasitic infections associated of marine origin are increasing in number and geographic range in response to climate change [456]. Cryptosporidiosis, giardiasis, and salt water schistosomiasis are the most common of these infections [453,457,458,459].

Two emerging human parasitic diseases of particular concern in the ocean environment are Anisakiasis (a zoonosis caused by the fish parasitic nematode, *Anisakis*) and Diphyllobothriasis (caused by the adult tapeworm, *Diphyllobothrium nihonkaiense*) [460]:

- Thousands of cases of anisakiasis have been reported, primarily from Japan but also from Europe and other parts of the world since the first case was reported in the 1960 [461,462]. An extensive survey carried out in the European anchovy *Engraulis encrasicolus* showed that rates of infection are as high as 70% among anchovy taken from fishing grounds in the Mediterranean Sea [463]. Spain is currently considered to have the highest incidence of anisakiasis in Europe [464].
- Diphyllobothriasis is associated with the consumption of raw Pacific salmon and is the most frequently occurring foodborne parasitic infection in Japan. *Diphyllobothrium nihonkaiense*, the causative agent, can grow to lengths of up to 10 meters in the human digestive tract and lay millions of eggs that are excreted in feces [460].

### Impacts of Ocean Pollution on Fish Stocks and Fisheries

Increasing pollution of the oceans, climate change and ocean acidification can cause changes in the marine food web and these changes can influence the abundance and geographic distribution of commercially significant fish species that are important human food sources. Species that are intolerant of pollution will decrease in number under the pressure of pollution and climate change, while more pollution-tolerant species will increase (**Text Boxes 7** and **8**).

**TEXT BOX 7: Climate-related collapse of a South African prawn fishery.**

A modelling study conducted off the coast of eastern South Africa showed that compromised production of penaeid prawns in the St Lucia estuary, an important nursery area, and eventual collapse of this shallow water fishery was associated with prolonged closure of an inlet [479].

The problem was that prolonged closure of an inlet to the estuary hindered the movement of post-larval shrimp into the nursery area and also blocked movement of juveniles out of the estuary to the trawling ground. Through feedback loops within the food web, these changes had knock-on effects on other commercially exploited species in the same fishing grounds, even on species that did not directly depend on estuaries, lowering their biomass and potential for commercial exploitation [480].

**Figure:**
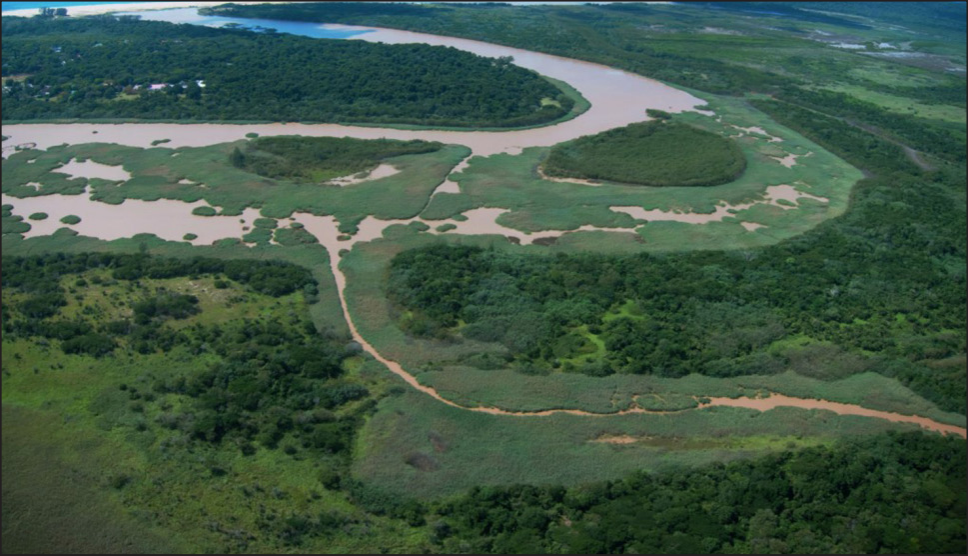
*Source*: CF MacKay, Oceanographic Research Institute, Durban, South Africa.

This case study illustrates that food security for humans can depend on the indirect effects of pollution and climate change that extend over several ecosystem types and are influenced by the geographical distribution of species across their life stages. In countries where subsistence fishers are reliant on fishing in estuaries, the effects on human food security can be devastating.

**TEXT BOX 8: Marine Viruses and Declines in Salmon Populations. Interaction with Pollution?**

The last three decades have seen large declines in salmon populations in both the Atlantic and Pacific Oceans. Recent studies investigating these declines using *in situ* hybridization, epidemiological surveys, and sequencing technologies have led to discovery of multiple new viruses. These viruses have been associated with disease among both wild and farmed salmon from different populations [495].

In these studies, fish were screened against a viral disease detection biomarker panel (VDD) that elucidates a conserved transcriptional pattern indicative of immune response to active RNA viral infection. Individual fish that were strongly VDD positive, but negative for any known salmon virus were subject to metatranscriptomic sequencing. This sequencing revealed viral transcripts belonging to members of the *Arenaviridae* (Salmon pescarenavirus: SPAV-1and 2), the *Reoviridae* (Chinook aquareovirus: CAV), and the *Nidovirales* (Pacific salmon nidovirus: PsNV), three divergent groups of highly pathogenic RNA viruses.

The distributions of the three viruses were markedly different:

- Both SPAV-1 and 2 were relatively widespread along the coast of southwestern British Columbia in ocean-caught Chinook and Sockeye salmon.
- CAV was not detected in any juvenile wild or hatchery Chinook salmon, but was detected in farmed fish on both the west and east coast of Vancouver Island.
- PsNV distribution was strongly associated with salmon-enhancement hatcheries, but was also detected in 18% of aquaculture Chinook and 3% wild Chinook. In hatchery fish, infection by PsNV was primarily localized to gill tissue, a pattern reminiscent of the respiratory disease caused by the related mammalian coronaviruses, such as MERS, SARS or COVID-19.

An unresolved question is whether spread of these viruses to salmon or severity of disease is enhanced by marine pollution.

A principal mechanism through which pollution alters the marine food web and affects fisheries is by causing changes in the abundance and composition of microalgae and other species that are the foundation of the marine food web [155,298,465,466]. Pollution that enters coastal waters through agricultural runoff and sewage discharges is typically rich in nutrients – nitrogen, phosphorus, and organic chemicals. Increased abundance of these materials results in proliferation of some, but not all species of microalgae. If the proliferating species are not the preferred food source of species above them, the composition of the entire food web can be altered and follow-on adjustments in the relative abundances of grazers and predators can ripple through multiple trophic levels [467]. If the end result is decreased species diversity, and the productivity of the few pollution-tolerant species that remain can seldom sustain food web, sharp reductions in catches of commercially important fish and food shortages can result.

Estuaries are highly sensitive to marine pollution. Estuaries are also vital nurseries for many commercially important fish species. In South Africa, for instance, 60% of exploited fish species inhabit estuaries as juveniles, and small invertebrates, which are abundant in estuaries, are the juveniles’ main food stock there [468]. The small invertebrates that populate estuaries are well able to cope with changing conditions of salinity and temperature caused by riverine and marine tidal influences [469]. However, these organisms can be highly susceptible to pollution, and coastal pollution can reduce invertebrate abundance and remove intolerant species entirely [470,471]. In these circumstances, the food security of the juveniles becomes precarious, and stocks of key fish species can decline. These estuarine effects are particularly important when pollution is widespread.

Short-term, high-impact pollution events can also result in food web alterations and reductions in seafood productivity. The most famous of these events in recent times have been the Deep Water Horizon oil spill in the Gulf of Mexico, and the Fukushima nuclear power plant accident in Japan. Both direct effects to individual species and indirect effects on the food web were apparent in these two events [472].

Climate change can also affect the health of estuaries and fish stocks. It can exert synergistic effects on marine ecosystems in concert with pollution. Climate change causes changes in rainfall that, in turn, alter runoff to estuaries and nearshore environments. In nutrient-poor areas, nutrients delivered from the land to the oceans via rivers are very important to sustain local food webs and fish production [473,474]. With changes in the global climate, estuaries in arid and semi-arid regions may receive less freshwater runoff, or receive large rainfalls over fewer days or in the wrong season. All of these changes compromise the nursery function of estuaries. These changes can result in increased or decreased salinity, more frequent or less frequent flooding, changes in energy supplies, frequent closures of inlets that hinder migration of marine species in and out of estuaries, and changes in the timing of inlet closure and opening such that they no longer synchronize with fish life stages [475,476,477,478].

Coastal marine ecosystems in and near cities, especially near rapidly growing megacities in developing countries and those with emerging economies are constantly exposed to pollution and other environmental stressors of human origin [481,482]. Losses and changes of habitat, increasing light and noise levels, and industrial chemical discharges impact fish populations in these areas, modifying their behavior and ultimately reducing the amounts of fish available to feed humans [483,484]. Dredging and coastal pollution increase turbidity, change the light regime in the water column, impact primary production, and affect migration and predator-prey interactions [481]. Increased foraging activity in artificially lit areas increases predation pressure on one trophic level, and in turn releases predation pressure on the next trophic level [485]. Noise pollution may affect fish and marine mammal communication, as well as the behavior of invertebrates. Artificial hard structures change habitat that might originally have been comprised of soft sediment. Such changes in habitat provide opportunities for invasive species [481,481]. All such modifications, especially when they are of large scale, cause changes in the food web, resulting in changed productivity patterns that alter ecosystem services to humans. Although human modifications can occasionally enhance habitat and increase fishery production (e.g., around artificial reefs), the negative impacts of human activity far outweigh their positive benefits on a global scale [481].

Reduced content of dissolved oxygen in seawater – ocean hypoxia – is another consequence of pollution and climate change that has negative impacts on fish stocks [486,487]. Ocean hypoxia is the result of terrestrial runoff that introduces nutrients to the seas, increases frequency of HABs, and leads to eutrophication and the formation of dead zones. Vast releases of organic matter from industry and waste water systems further compound these effects. Hypoxic areas and dead zones are increasing in seas across the globe [488]. Additional contributory factors are sea surface warming, which reduces oxygen solubility in the oceans and changes stratification patterns that, in turn, may reduce ocean mixing and prevent re-oxygenation [489]. All of these effects are most pronounced in coastal and continental shelf areas of the oceans – the regions of the seas that produce 90% of commercially exploited fish species [490].

Ocean acidification, a direct consequence of increasing concentrations of atmospheric CO_2_, is another environmental factor of human origin that can affect fish stocks. By inhibiting the growth of calcified primary producers (calcified phytoplankton such as coccolithophores or foraminifera) or zooplankton (krill, pteropods) at the base of the food web, ocean acidification may alter the food chain production [491,492,493].

In addition to decreasing seafood production, ocean acidification may also alter seafood quality. Researchers asked 30 volunteer testers to assess the gustatory quality (appearance, texture, and taste) of shrimp raised at different pH levels [494]. The test was conducted under the supervision of a chef. Decreased pH significantly reduced appearance and taste scores. Thus shrimp maintained at a pH of 8.0 had a 3.4 times higher likelihood of being scored as the best shrimp on the plate, whereas shrimp maintained at a pH of 7.5 had a 2.6 times higher likelihood of being scored as the least desirable shrimp on the plate, a result that may have socio-economic implications.

Increased bioaccumulation of pollutants in the food web will be a further impact of pollution, ocean acidification, and climate change on fisheries. Concentrations of PCB and MeHg in top predators such as killer whales are projected to increase by 3% to 8% by 2100 under a high-carbon-emission scenario compared to a control scenario [496]. MeHg accumulation is particularly sensitive to variations in emission scenarios with a trophic amplification factor generally ten times higher than for PCBs.

**Figure 14:**
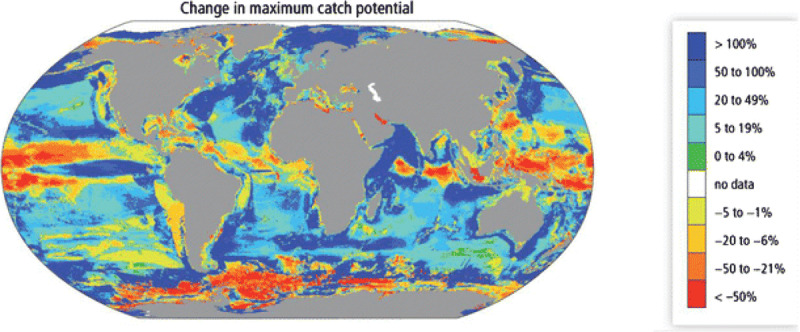
Global changes in maximum fish catch potential. *Source*: IPCC.

Most of the world’s fish stocks are already either fully or over-exploited [497]. Pollution, ocean warming and ocean acidification add to these pressures. The warming of the marine environment during the last two decades has reduced the productivity of marine fisheries worldwide and contributed to a 4.1% decrease of maximum sustainable yield of several fish populations, with some regions showing losses of as much as 15 to 35% [498] (Figure 14). Almost 90% of the large predator fish species have been removed from all seas around the globe leading to the collapse of certain species, such as Newfoundland Cod [499]. Increasing global demand for fish as a food source has driven rapid increase of aquaculture, which has resulted in high demands on capture of large wild fish used for feeding of farmed fish [500].

Reductions in fish stocks have direct impacts on human health by jeopardizing food security in coastal communities in low-resource countries [501]. Declines in fish catches deprive people of protein, as fish is a highly important source for nearly 20–30% of the human population [502]. Reduced fish consumption results not only in protein malnutrition, but also in reduced consumption of essential micronutrients, including Vitamin A, iron, Vitamin B12, and omega-3 fatty acids among vulnerable populations [502]. These impacts fall most heavily on poor countries [503], but negative impacts are seen also in areas of economically developed nations where shellfish make up a substantial part of the commercial and traditional subsistence fisheries such as Alaska, USA [504].

Continuing reductions in fish stocks and in the productivity of the oceans may be anticipated in future years due to the combined effects of pollution, sea surface warming, ocean acidification, and other wide-scale ecological impacts. Poleward migration of many commercially important marine species towards higher latitudes is occurring already and will increase further. Ocean acidification and pollution will damage tropical and subtropical coral reefs thus reducing the abundance of reef fish species [502].

Additional effects on fish stocks could be mediated through changes in major ocean currents. Thus, there is growing concern that climate change could disrupt the highly productive Eastern Boundary Upwelling Systems, such as the Humboldt and Benguela currents in the South Atlantic Ocean that rely on the upwelling of nutrient-rich water to stimulate productivity and produce large fish yields. These changes could jeopardize the security of coastal fishing communities that depend on them for their food and their livelihoods [505]. These grave dangers justify the proactive policy of designating Marine Protected Areas in critical areas of the seas.

### Impacts of Ocean Pollution on Vulnerable Human Populations

Ocean pollution, like all forms of pollution, has disproportionately severe health impacts in low-income and middle-income countries [24]. It especially affects coastal communities in low-income countries that are dependent on the oceans for their food and livelihood. The effects of pollution and climate change fall especially heavily on these populations because they do not have the resources or the infrastructure to buffer diminished ecosystem services. Thus they are highly vulnerable to the increasingly frequent HAB events and HAB toxin exposures that are the consequences of worsening coastal pollution. Poignant examples are seen in small island nations [17] and in the countries of the Western Indian Ocean region – Comoros, Mauritius, Mozambique, and Somalia [506].

Indigenous peoples are another group highly vulnerable to ocean pollution and its health effects. Their heightened vulnerability to ocean pollution reflects the fact that these groups consume up to 15 times more seafood per year as non-indigenous peoples [20,507]. They are also at high risk of exposure to plastic particles, methyl mercury, POPS, and manufactured chemicals that concentrate in marine species.

Populations in the circumpolar regions – indigenous peoples as well as non-indigenous populations such as the people of the Faroe Islands [66] – are yet another group placed at high risk by worsening ocean pollution. The increasingly heavy atmospheric deposition in northern waters of mercury, PCBs, and other POPs transported poleward on the winds from distant population centers has led to accumulations of hazardous chemicals in the tissues of the predator fish species and marine mammals that are major components of these populations’ diets. This, in turn, has led to increasing toxicity – toxicity that has been well documented through epidemiologic studies [67,68,508,509,510].

**Dietary Change.** As seafood becomes increasingly scarce and more contaminated by chemical pollutants [66] and HAB toxins [343], people in low-income countries, indigenous areas, and the circumpolar regions are forced to turn away from their traditional fish-based diets and to eat more meat and poultry. This dietary change places them at risk of all the health consequences of the “Western” diet – obesity, type 2 diabetes, cardiovascular disease, and cancer. This trend is evident in Alaska native populations and appears to have contributed to the deteriorating health status of these groups [511].

In high-income countries, consumers’ perception of the safety of seafood has led to a reduction in demand for shellfish, and this change has had severe economic consequences for the shellfish industry [512]. The lack of diagnostic tools and treatment options for HAB-related illnesses leads to increased psychological stress in fishing communities [513,514].

**Ocean Pollution as a Risk factor for Migration.** Migration is another consequence of ocean pollution, climate change and declining fish stocks. Study of environmentally induced migration has grown in recent years [515]. Of particular importance has been emergence of the concept of “environmental refugees” [516], people who have been forced to leave their homes because of pressures created directly or indirectly by anthropogenic environmental, ecological and climate change [517]. Migration and conflict are now considered key mechanisms through which climate change and other environmental stressors increase frequency of migration and thus create environmental refugees [517,518,519,520].

The 2015 Rockefeller-*Lancet* Commission on Planetary Health has identified migration as a major concern for human health and development and a priority area of research [2]. Ocean pollution and other ecosystem changes are already triggering environmental migration and will continue to do so over the coming decades [497,521,522].

While global ecological trends and climate change impacts have been a priority of the research community, complex implications at local scales are less well understood. Climate-induced triggers for migration include sea level rise, salinization of fresh water supplies, changing patterns of flooding and draughts, pest and alien species invasion, changing weather patterns, and ocean acidification [523]. These drivers can act concurrently and produce synergistic effects on human health and well-being. In combination with pollution, changes in land use, loss of biodiversity, mismanagement of resources, and collapse of the fisheries on which coastal populations rely for food and economic security [2,524,525], are multiple drivers that lead to vulnerability, threatened livelihoods, culture and political instability, and social injustice [523]. They reduce food and water security and increase risk of starvation [8,526,527]. These factors lead also to loss of property, shelter and human life [504,528,529,503,530].

### The Critical Importance of Ocean Monitoring

Robust monitoring of ocean pollution is important for protecting human health and safeguarding marine ecosystems. Need for monitoring will become increasingly great as the global climate continues to change, seas continue to warm, extreme weather events become more frequent, and human impacts on coastal, estuarine, and deep-ocean environments continue to grow.

Monitoring provides information on background levels of pollution, tracks trends, maps geographical variation, identifies ‘hot spots’, provides early warning of impending crises, guides interventions against pollution, and evaluates the effectiveness of interventions. Monitoring of chemical and physical processes in the oceans is essential to tracking sea surface warming, ocean acidification, and the consequences of these phenomena on marine ecosystems, including their impacts on the frequency of HABs and the spread of marine pathogens.

The great importance of ocean monitoring in guiding the protection of human and ecosystem health was recognized in a seminal 2002 report that recommended establishing programs to monitor ocean pollution [531]. That report called for the establishment of multidisciplinary research programs to address the intersection between ocean and human health. Such programs have now been established in the United States and Europe. They provide an essential complement to ocean monitoring.

The Health of the Oceans (HOTO) Module of the Global Ocean Observing Systems (GOOS) is a key international initiative in ocean monitoring [532]. HOTO employs a range of sampling strategies across a variety of temporal and spatial scales using agreed standards and methodologies to track the effects of anthropogenic activities, ocean pollution in particular, on human health and marine resources. HOTO and other global and regional ocean monitoring systems are generating data showing the impacts of maritime and navigation activities; trends in ocean acidification and coral reef destruction; trends in fish stocks; introductions of invasive species; changes in sea surface temperature; the spread of life-threatening bacteria and harmful algae, and trends in plastic pollution [533,534].

Improved monitoring of all forms of ocean pollution and better documentation of pollution-related patterns of human exposure and disease will improve estimates of the contribution of ocean pollution to the Global Burden of Disease [41].

#### Monitoring Toxic Chemicals and Plastics in the Ocean Environment

Monitoring of chemical and plastic pollution in the oceans has been ongoing for decades. One approach has been direct measurement of discharges of pollutants such as waste plastics into the seas from land-based sources, and tabulation of the number and frequency of discharge events such as oil spills. Under the aegis of the Horizon 2020 Initiative for a Cleaner Mediterranean, the European Environment Agency, and UNEP-MAP have defined a set of indicators that will potentially enable an integrated assessment of key land-based sources of pollution in European seas, including solid waste and marine litter.

A key monitoring strategy for toxic chemical pollutants is to measure concentrations of indicator pollutants in seawater or in organisms that are “sentinel species”. Since the 1970s, the U.S., the European Environment Agency, and the International Mussel Watch Program have measured geographic patterns and temporal trends in concentrations of organic chemical and heavy metal pollutants along the coasts, through analysis of residues in bivalve mollusks [535]. These programs have identified locations where heavy metals, POPs, and pesticides are most highly abundant and have highest potential to contaminate seafood. These programs have documented that pollutant concentrations are highest near urban areas [536].

Evaluation of molecular biomarkers of exposure to chemical contaminants is an important complement to direct measurement of chemicals [531,537]. Biomarkers have been used to assess exposures and early biological effects of exposures to oil spills, PCBs, dioxins, toxic metals, and endocrine disruptors [538]. Pollutant levels in broad areas of the open ocean can be inferred by analysis of tissue levels in large ocean species that serve as biological monitors. Thus, measurement of levels of chemical pollutants and of molecular biomarkers of exposure has been done by analysis of skin biopsies of sperm whale [536]. Studies in tissues of large sharks and finfish (yellowfin tuna) provide similar data [210,539].

Future Directions in Monitoring of Chemical and Plastic Pollution in the Oceans.

- Airborne and satellite sensors hold great promise for advancing the science of chemical and plastic pollution monitoring. There now exist many platforms and sensor technologies with the potential to scan large areas of the oceans continuously and to provide information on a range of conditions in nearly real-time. These sensors can map and track the distribution of pollutants such as oil spills and plastic waste. Plastic monitoring may be a proxy for monitoring POPs and other toxic chemicals associated with plastic. Remote sensors can also detect HABs [540,541].
- To track ocean pollution by mercury and POPs, the Group on Earth Observations (GEO) has developed the Global Observation System for Mercury (GOS4M) and is developing a Global Observation System for Persistent Organic Pollutants (GOS4POPS).
- To store and analyze data on POPs levels in marine biota, the Global Monitoring Plan (GMP) Data Warehouse established under the auspices of the Stockholm convention is a growing resource. It could be expanded and linked to data on POP levels in human milk and serum in high-risk populations such as people in the circumpolar regions.
- Systematic measurement of pollutant levels in mesopelagic or midwater fishes could be a means for assessing the global status of ocean pollution in the future, as a companion to studies of large fish and marine mammals. Midwater fishes live in the seas at intermediate depths, 200–1,000 m below the surface, and are present in all the oceans of the world.
- Passive samplers and sensors are being developed and applied to assess the distribution and concentrations of pollutants in waters around the world, and to detect new pollutant chemicals [540,541].

#### Monitoring HABs

Several international and European systems currently capture and disseminate information about HAB events, their predisposing factors, and HAB- related illnesses [542,543]. Other initiatives are being coordinated by the Intergovernmental Panel for Harmful Algal Blooms (UNESCO, IPHAB) collaboration. Specific initiatives are summarized in the following, Tables 3 and 4:

**Table 3:** European Ocean Monitoring Programs.


Data from the European Space Agency Copernicus Sentinel-3 satellite Ocean and Land Color Instruments (OLCI) are used in near real-time to make initial water quality assessments and quickly alert managers to potential problems and emerging threats related to cyanobacteria [544]. The IOC International Oceanographic Data Exchange Programme (IODE) hosts the *Harmful Algae Event Data Base* (HAEDAT) containing and summarizing complex quality-controlled, regularly updated information on HAB events worldwide. These curated open access databases are the base of the Global HAB Status report supported by IOC-UNESCO, ICES, PICES and the International Atomic Energy Agency (IAEA) [323]. The International Food Safety Authorities Network (INFOSAN) facilitates rapid information exchange across borders during events that threaten food safety [545]. The Rapid Alert System for Food and Feed allows rapid information sharing to protect food supplies and document foodborne outbreaks across Europe [546].


**Table 4:** United States Ocean Monitoring Programs.


CDC created the One Health HABs System (OHHABS) in 2016 to allow US states to report on both human and animal HAB-related illness and information about the blooms themselves [547]. Data collected through OHHABS will enable updating of case definitions for HAB-related illness, treatment regimens, and clinical analyses. The CDC’s Environmental Public Health Tracking Program [547] is collaborating with OHHABS to geographically track HAB events and link these events to illness cases and outbreaks. CDC is working with the American Association of Poison Centers to identify outbreaks of HAB-related disease using the National Poison Data System, which records data from every call made to U.S. poison centers. An algorithm identifies potential outbreaks [548]. EPA created the Cyanobacteria Assessment Network (CyAN) to support the management and use of U.S. lakes and reservoirs [549]. The Food and Drug Administration has established the Hazard Analysis and Critical Control Points (HACCP) program [550]. Elements of this programs are: 1) classification of areas for safe shellfish harvesting; 2) water quality monitoring; 3) marine biotoxin management; 4) monitoring of procedures for processing, shipping, and handling of live shellfish; 5) establishment of laboratory methods for monitoring microbiological contaminants and marine biotoxins; and 6) enforcement of shellfish safety regulations. These programs have been effective in minimizing human illnesses from consumption of toxic shellfish while allowing fisheries industries to persist in regions threatened by recurrent HABs.


#### Monitoring Bacterial, Viral, and Parasitic Pathogens

Serious challenges impede the detection, quantification and prediction of viral, bacterial, and parasitic pathogens in seafood, shellfish, and oceanic waters as well as in aquaculture operations. Although molecular diagnostics and other tools have improved dramatically over the past two decades [454,551], additional advances are required to better detect and quantify pathogens in water, seafood products, aquaculture facilities, and shellfish meats [552].

The significant relationships observed between pollution concentrations, rising sea surface temperatures, *Vibrio* infections and HABs have catalyzed the development of modeling efforts. These models incorporate multiple layers of geocoded data and are designed to generate predictive forecasts [553]. New technologies such molecular and bioinformatics-based diagnostics [410,425,554], metabarcoding, “big data” mining and machine learning may be expected to contribute to further development of these efforts [40,555,556]. Implementation of real-time PCR-based approaches has already been shown to be a useful tool for diagnosing *V. vulnificus* wound infections [554].

A mapping tool developed by the European Centre for Disease Prevention and Control (ECDC) [416] is now operational and is providing 24-hour updated *Vibrio* risk data freely available to the community. However, this system has not yet been implemented by all EU Member States. Also, it needs to be further developed to incorporate relevant variables associated to major climatic events that have been proven to have an impact.

## Successes in Prevention and Control of Ocean Pollution

A key finding of the 2018 *Lancet* Commission on Pollution and Health is that much pollution can be controlled and pollution-related disease prevented [24]. The Commission noted that most high-income countries and an increasing number of middle-income countries have curbed their most flagrant forms of pollution by enacting environmental legislation and developing regulatory policies. These policies are based on science and are backed by strict regulation. They set targets and timetables, they are adequately funded, and they are based on the “polluter-pays principle”. Air and fresh water in these countries are now cleaner, health has improved, and longevity has increased. The *Lancet* Commission concluded that pollution control is “a winnable battle” [24].

An additional benefit of pollution control is that it is highly cost-effective. Rather than stifle economic growth and depress job markets, as is often claimed, pollution control has, in fact, been shown to boost economies, increase human capital and create prosperity. It creates these gains by preventing disease and premature death, reducing productivity losses, and preventing environmental degradation. In the United States, air pollution has declined by 70% since passage of the Clean Air Act in 1970, and every $1 (USD) invested in control of air pollution has returned an estimated benefit of $30 (USD) (range of estimate, $4–88 USD) [24]. Likewise, the removal of lead from gasoline has boosted economies in countries around the world by increasing the intelligence of billions of children who have come of age in relatively lead-free environments and who are thus more intelligent and productive [24].

The strategies used to control pollution of air and fresh water are beginning to be applied to the prevention and control of ocean pollution. Key to the effectiveness of these efforts has been the recognition that 80% of ocean pollution arises from land-based sources [29]. Accordingly, successful marine pollution control programs have identified, targeted, and reduced releases from important land-based polluters. They have been guided by multi-scale monitoring that tracks pollutant discharges, measures pollutant levels in the seas and in marine biota, and assesses human exposures and health outcomes. They have been backed by strict enforcement. They have engaged civil society and the public by making their strategies, their data, and their progress reports available on open-source platforms.

These strategies are beginning to make a difference. As is described in the case studies presented below (**Text Boxes 9–13**), industrial discharges have been reduced in some areas, plastic pollution reduced, agricultural runoff mitigated, and sewage more effectively treated. Coastal contamination has been reduced, levels of toxic chemicals in marine organisms have declined, the frequency and severity of HABs have been reduced, polluted harbors have been cleaned, estuaries have been rejuvenated, shellfish beds [557] and aquaculture operations [558] have been protected, fish stocks have rebounded, and coral reefs have been restored. The successes in control of ocean pollution achieved to date demonstrate that broader prevention is possible.

**TEXT BOX 9: Control of Plastic Pollution in the Mediterranean.**

Plastic pollution is one of the most pervasive and highly visible threats to the health of the oceans today. Once discharged into the natural environment, plastic can take up to 500 years to disappear. The Mediterranean Sea is particularly vulnerable to plastic pollution because of its semi-enclosed geographical location, and the intensity of its maritime transport, fishing, industry, and tourism. With more than 3000 billion microplastic particles estimated to be in its waters, the Mediterranean is the most polluted sea in the world.

In 2015, the Prince Albert II of Monaco Foundation, the Tara Ocean Foundation, Surfrider Foundation Europe, the MAVA Foundation and the IUCN joined forces to launch the Beyond Plastic Med (BeMed) Initiative. BeMed’s objectives are to bring together and support the stakeholders involved in the fight against plastic pollution in the Mediterranean, implement sustainable solutions, encourage the search for new solutions, and mobilize stakeholders and the general public through knowledge and sharing of best practices.

To achieve its objectives, BeMed supports projects every year that aim to reduce plastic pollution at source by minimizing the use of plastic, finding alternatives, improving waste collection systems, raising awareness, collecting data, and helping to implement new regulations. To date, 53 projects in 15 countries have been supported.

In addition to providing financial support to these efforts, BeMed works to build and coordinate the network of active Mediterranean stakeholders by facilitating the sharing of experience and knowledge and by creating links between organizations. Principal Investigators of the projects supported by BeMed are gathered every year for a day of exchange during Monaco Ocean Week. In addition, stakeholders working on similar topics or in the same region are put in contact with one another to foster collaborations, share knowledge, and thus reinforce the effectiveness of their actions. Replication of successful actions is strongly encouraged.

Since early 2020, BeMed has also engaged the private sector in the fight against plastic pollution by forming of a consortium of companies committed to preventing plastic pollution of the Mediterranean. This consortium includes players at every stage in the plastics value chain – producers of plastic raw materials, plastic manufacturers, producers of plastic-containing consumer products, retailers, and waste management companies – in order to draw companies into a common dynamic of pollution reduction on a Mediterranean-wide scale. Activities of this consortium are structured around two working groups: a group promoting dialogue between scientists and industrialists to clarify the key issues, and a group dedicated to implementing pilot projects in the field. An advisory committee of scientific experts ensures the effectiveness and sustainability of the proposed solutions.

**TEXT BOX 10: Control of Persistent Organic and Metal Pollutants in European Waters.**

The European Environmental Agency [27] tracks concentrations of eight indicator pollutants in the waters surrounding Europe. These include three metals – mercury, lead, cadmium, and five persistent organic pollutants (POPs) – hexachlorobenzene (HCB), lindane, PCBs, DDT (using DDE as a proxy), and the polycyclic aromatic hydrocarbon (PAH) BAP (benzo[*a*]pyrene).

The first seven of these substances have been banned from use in Europe, and their discharges into the seas have declined, in some cases sharply. Thus mercury concentrations in North Sea blue mussels have fallen, as have PAH and PCB concentrations in monitored areas in the North Atlantic [27,208]. (See Figure)

**Figure 15:**
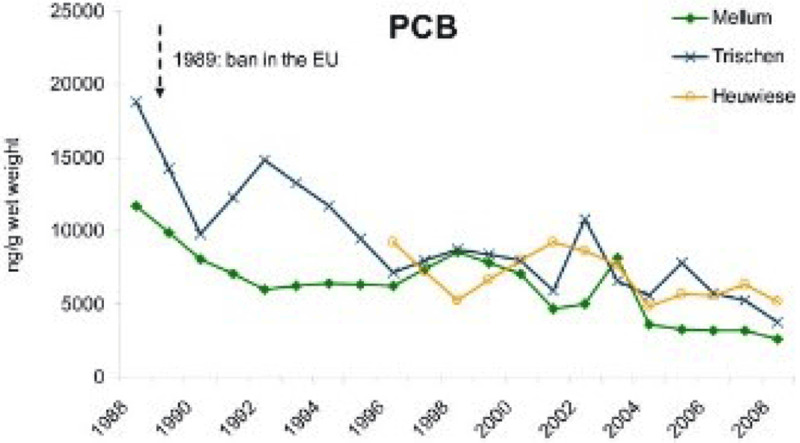
**Concentrations of PCBs in archived herring gull eggs from three locations on the North German coast, 1988–2008** [208] *Source*: Fleidner et al. (2012), https://doi.org/10.1186/2190-4715-24-7, Creative Commons, license CC BY 2.0.

These trends document the power of bans on hazardous chemicals in reducing chemical pollution of the oceans. However, despite these successes, levels of all eight of these pollutants remain elevated in European waters and are anticipated to remain unacceptably high for many decades to come. Pollutant levels will be especially slow to decline in Arctic waters where cold temperatures slow chemical degradation [208].

**TEXT BOX 11: Successful Control of Harmful Algal Blooms in Japan’s Inland Sea.**

A striking example of successful control of HABs through a science-based prevention program is seen in the case of the Seto Inland Sea in Japan.

In the Seto Inland Sea, the number of visible “red tides” (high biomass blooms) increased seven-fold between 1960 and the mid 1970s. This increase paralleled increases in industrial production and in chemical oxygen demand (COD) from domestic and industrial wastes discharged into the sea.

In 1973, Japan instituted the Seto Inland Sea Law to reduce COD loadings to half of the 1974 levels over a three-year period. As a result, the number of red tides began to decrease in 1977, dropping to, and remaining at levels approximately one-third of peak frequency [332,559]. These data demonstrate an increase in phytoplankton abundance due to over-enrichment of coastal waters, followed by a proportional decrease in blooms when that loading was reduced. Importantly, toxic blooms (in this instance, those that caused fish mortalities or other fisheries damage) also decreased after the loadings were reduced.

The legislative or policy changes implemented in the Seto Inland Sea demonstrate that control of sewage and industrial discharges has the potential to prevent some HABs. Nevertheless, there are other important sources of nutrients to coastal waters, and these are more difficult to control, given the increased population pressures and the need to feed a growing world population. In particular, the steady expansion in the use of fertilizers for agricultural production represents a significant and worrisome source of plant nutrients to coastal waters.

**TEXT BOX 12: Boston Harbor Restoration: From a “Harbor of Shame” [560] to a “Great American Jewel” [561].**

**Background.** Boston Harbor is an estuary of Massachusetts Bay that provides services worth $30–100 billion to society [562]. Beginning in the nineteenth century, industrialization, urban development, and population growth led to heavy pollution of the harbor [560,562]. The construction of wastewater treatment plants at Deer Island in the 1950s and Nut Island in the 1960s further exacerbated this problem. The amount of wastewater delivered to these plants often exceeded the plants’ capacities, and by the 1980s, they discharged 350 million gallons of untreated wastewater into the harbor daily. The wastewater devastated water quality, marine habitats, and recreational activities [562]. Boston Harbor became one of the most polluted harbors in the US [560].

**Solution.** Local organizations had already begun advocating for a cleaner Boston Harbor when Congress passed the Clean Water Act in 1972 [562]. This law catalyzed the cleanup of the polluted harbor. The City of Quincy and the Conservation Law Foundation sued the Commonwealth of Massachusetts for failing to comply with the Clean Water Act, and in 1986, a court-ordered cleanup began [563].

The cleanup strategy consisted of several steps, including [563]:

+ Improvements to the 1950s-era treatment plant on Deer Island
+ Construction of a new Deer Island Treatment Plant
+ Transfer of Nut Island Treatment Plant flows to Deer Island
+ Creation of a 9.5-mile outfall to discharge treated effluent from Deer Island into Massachusetts Bay
+ Conversion of sludge into fertilizer, rather than discharge
+ Combined Sewer Overflow projects to protect sensitive waters from overflows.

**Results.** The Boston Harbor cleanup strategy has had many accomplishments. Most notably, sewage waste that had previously undergone little or no treatment before discharge into the Harbor is now subjected to state-of-the-art treatment [561]. As a result, the harbor has steadily become cleaner, as illustrated by data taken from 70 locations throughout the harbor since 1989 [561]. The cleanup resulted additionally in elimination of hepatic neoplasia in winter flounder in the harbor, which had previously been highly prevalent [564].

**Conclusion.** The cleanup of Boston Harbor was effective, and the Harbor is now known as the “Great American Jewel” [561]. To continue the work, policymakers are now addressing current threats to the health of the harbor, including sea level rise, habitat destruction, and invasive species [560].

**TEXT BOX 13: Restoration of Coral Reefs in American Samoa.**

**Background.** American Samoa is a US territory consisting of seven islands in the South Pacific [565]. The territory contains coral reefs that are both diverse and essential: 2,700 marine species depend on the reefs for shelter, and 55,000 people depend on the reefs for sustenance and employment. Over the past several decades, several disturbances have threatened the reefs (Craig et al., 2005). In the latter half of the 20th century, tuna canneries regularly released nutrient-rich wastewater to Samoan coastal waters leading to an increase in coral-eating plankton and a decrease in corals. By the late 1970s, after an outbreak of crown-of-thorn starfish, only 10% of the corals remained. The problem was further exacerbated by the overfishing of parrotfish, which typically protect corals by consuming harmful algae [565].

**Solution.** To address the problems confronting the reefs of American Samoa, a suite of solutions was implemented. In 1986, the Fagatele Bay National Marine Sanctuary was created, thereby imposing restrictions on pollution and fishing. Then, in 1991, the government diverted wastewater pipes to combat the increase in coral-eating plankton. In 2000, spearfishing was banned to protect parrotfish [565].

**Results.** The reefs of American Samoa have slowly but surely recovered. In the past nine years, the reefs’ coral cover (proportion of the reef’s surface covered in coral) has increased from 25 to 36%. Compared to the Great Barrier Reef’s coral cover of 14%, the American Samoa reefs are faring well [565].

**Conclusion.** The reefs of American Samoa are considered to be in “good” condition [566], but they continue to face ongoing threats, such as pollution, red tides, coastal sedimentation, and ocean acidification [565,566,567]. To protect the reefs, these threats should be addressed.

Programs for the control of ocean pollution create multiple benefits. They boost economies, increase tourism, bring back commercial fisheries, and improve human health and well-being. These benefits will last for centuries.

The following Text Boxes (**Text Boxes 9–13**) present case studies of successes in control of ocean pollution. A central element in each of these examples has been careful documentation of progress against pollution through robust monitoring. Five case studies are presented here and additional studies are presented in the Supplementary Appendix to this report.

## Conclusions

Ocean pollution is a global problem. It arises from multiple sources and crosses national boundaries. It is worsening and in most countries poorly controlled. More than 80% arises from land-based sources.

Plastic waste is the most visible component of ocean pollution and has deservedly attracted much attention. It kills seabirds, fish, whales and dolphins. It breaks down into plastic microparticles and nanoparticles and fibers containing myriad toxic and carcinogenic chemicals. These chemical-laden particles are absorbed by fish and shellfish, enter the marine food chain, and can ultimately be consumed by humans. Their dangers to human health are only beginning to be assessed.

Additional components of ocean pollution include mercury released by the combustion of coal and from small-scale gold mining; petroleum discharges from oil spills and pipeline leaks; persistent organic pollutants, such as PCBs and DDT; thousands of manufactured chemicals, many of unknown toxicity; pesticides, nitrogen, and phosphorus from animal waste and agricultural runoff; and sewage discharges containing multiple microbial contaminants. In concert with sea surface warming and ocean acidification, ocean pollution leads to increasing frequency and severity of HABs, destruction of coral reefs, and spread of life-threatening infections.

Pollution of the oceans can be directly ascribed to the “take-make-use-dispose” economic paradigm that Pope Francis has termed, “the throwaway culture” [568]. This linear, economic paradigm focuses single-mindedly on gross domestic product (GDP) and on endless economic growth [569]. It views natural resources and human capital as abundant and expendable and gives little heed to the consequences of their reckless exploitation [2,8]. It ignores the precepts of planetary stewardship [102,568,570]. It is not sustainable [571].

Leaders at every level of government - city, regional and national – as well as sustained engagement by the international community and civil society will be key to the control of ocean pollution and the prevention of pollution-related disease.

Eight key conclusions that emerge from this analysis are the following:

1. **Pollution of the oceans is widespread, worsening, and poorly controlled. Human activity that releases unwanted, often dangerous waste materials into the sea is the major source.**
  - Ocean pollution is a complex mixture of plastic waste, toxic metals, manufactured chemicals, oil spills, urban and industrial wastes, pesticides, fertilizers, pharmaceutical chemicals, agricultural runoff, and sewage. More than 80% arises from land-based sources. Chemical and plastic pollutants have become ubiquitous in the earth’s oceans and contaminate seas and marine organisms from the high Arctic to the abyssal depths.
2. **Ocean pollution has multiple negative impacts on human health and well-being. The magnitude, severity and geographic ranges of these effects are increasing.**
  - Consumption of contaminated seafood is the main route of human exposure to chemical pollutants, HAB toxins, and plastic microparticles and microfibers in the oceans.
  - Mercury, PCBs and other persistent pollutants accumulate to high concentrations in fish and marine mammals consumed by humans. Exposures of infants in the womb to these toxic materials through maternal consumption of contaminated seafood can damage developing brains, reduce IQ, and increase children’s risks for autism, ADHD, and learning disorders. Adult exposures to methylmercury increase risks for dementia and cardiovascular disease.
    - Coal combustion is a major source of marine mercury pollution.
    - Artisanal, small-scale gold mining is a second source of mercury pollution.
    - Omega-3 fatty acids and other beneficial nutrients unique to seafood can partially mitigate the injuries caused by mercury and POPs. Several groups have disseminated guidance on safe, sustainable seafood consumption [293,294,295].
  - Manufactured chemical pollutants – phthalates, bisphenol A, flame retardants, organophosphorus compounds, organotin compounds, and perfluorinated chemicals, many of them released into the oceans via plastic waste – are known to have multiple negative effects on human health that include cardiovascular disease, developmental disorders, endocrine disruption, depression of immune function, decreased fertility, and cancer.
  - Plastic microbeads and microfibers formed by the breakdown of plastic waste and manufactured as plastic microbeads contain many of the above-listed manufactured chemicals. These chemical-laden microscopic particles appear capable of entering the marine food web and concentrating in species consumed by humans. The burden of disease associated with human exposures to these chemical-laden particles and fibers is not yet known.
  - Coastal pollution by industrial discharges, agricultural waste, and human sewage leads to increasing frequency and severity of HABs – “red”, “green”, and “brown tides”. These blooms produce potent natural toxins such as ciguatera toxin and domoic acid that can concentrate to high levels in fish and shellfish. When ingested, these toxins can cause severe neurological disease and rapid death. HAB toxins can also become airborne and trigger asthma and other respiratory diseases.
  - Coastal pollution in concert with sea surface warming stimulates overgrowth of dangerous pathogens, most notably *Vibrio* species. Coastal pollution also increases antimicrobial resistance (AMR) in marine pathogens. With worsening coastal pollution and rising sea surface temperatures, concern is great that diseases caused by marine pathogens could spread into new, previously unaffected areas, especially places in the Global South where infrastructure is poorly developed and public health systems are weak.
  - Declines in seafood stocks caused by pollution, ocean warming, ocean acidification and overfishing threaten the health and well-being of the millions of people worldwide who depend on the seas for their food and their livelihood.
3. **Ocean pollution has multiple harmful effects on marine ecosystems. These effects can have negative impacts on human health.** Plastic pollution kills fish, seabirds, whales, and dolphins. Pharmaceutical waste contributes to the destruction of coral reefs. Increasing absorption of carbon dioxide into the oceans causes ocean acidification, destroys coral reefs and dissolves calcium-containing plankton at the base of the marine food web. Petroleum-based pollutants and POPs impede the production of oxygen by beneficial marine microorganisms.
4. **Ocean pollution is deeply unjust.** Ocean pollution and all its negative impacts fall disproportionately on people in small island nations, indigenous communities, coastal communities in the Global South, and fishing communities worldwide. These are populations that create only miniscule amounts of pollution. Most of the pollution to which they are exposed arises from sources far away. This is environmental injustice on a global scale.
5. **Ocean pollution is inadequately charted.** Current knowledge of ocean pollution and its impacts on human health is still at a relatively early stage. Information on the geographic distribution and concentrations of pollutants in the oceans is fragmentary and confined mostly to the seas that border high-income countries. Likewise, information on the sizes of the human populations exposed to ocean pollution and their levels of exposure is scant. Data that could support the development of estimates of the contribution of ocean pollution to the global burden of disease (GBD) are only beginning to be developed.
6. **Ocean pollution can be prevented and controlled.** Like all forms of pollution, ocean pollution can be prevented. The most effective prevention strategy is to control the land-based sources responsible for 80% of the pollutants that enter the seas.
  Prevention is achieved through identifying and quantifying pollution sources and then deploying data-driven control strategies that are based on law, policy, and technology and backed by enforcement. Many countries have used these tools to successfully control air and water pollution, and these programs have proven effective as well as cost-effective. The same strategies are now being applied to prevention and control of ocean pollution. The case studies in successful control of marine pollution presented in this report demonstrate that broader control is feasible.
  Prevention of ocean pollution will require recognition by policy-makers and the global public that pollution can indeed be prevented – that it is not the unavoidable price of economic progress. It will require understanding additionally that pollution control creates many benefits. Control of ocean pollution improves the health of the oceans, boosts economies, enhances tourism, restores fish stocks, prevents disease, extends longevity, and enhances well-being. These benefits will last for centuries.
  Ultimate and sustainable prevention of chemical pollution of the oceans will be achieved through wide-scale adoption of non-polluting, renewable fuels, transition to a circular economy, and adoption of the principles of green chemistry (**Text Box 15**).
7. **Proposals for Removal of Pollutants from the Oceans are of Limited Value.** Various strategies have been proposed for removal of plastic waste from the oceans [575]. Removal of plastic pollution by passive collection or vacuuming is not a viable strategy because of the extremely wide distribution of plastic waste in the oceans, their varying sizes from visible to submicroscopic, and the likelihood of by-catch of marine species.
  Other remediation strategies have explored breaking down synthetic microplastic polymers in the oceans through the use of microorganisms [576]. A number of fungal and bacterial strains possess biodegradation capabilities and have been found capable of breaking down polystyrene, polyester polyurethane, and polyethylene. A specialized bacterium is able, for example, to degrade poly(ethylene terephthalate) (PET) [577]. Such microorganisms could potentially be applied to sewage discharges in highly localized environments, but scrupulous due diligence will be required prior to their wider deployment to avoid unintended consequences [578].
  Bloom control – actions taken to suppress or destroy HABs – has been proposed, but is challenging and controversial. The science in this area is rudimentary [331]. Physical removal of algal cells from the water column using clay flocculation is currently the only strategy in routine use. In South Korea a clay called “yellow loess” has been used since 1996 to control HAB blooms that threaten aquaculture [579]. Likewise the Chinese have used clay to control algal blooms for over 20 years, with wide-scale applications covering up to 100 km^2^ [580].
  In sum, it is far more effective and also more cost-effective to prevent the entry of pollutants into the world’s oceans than to try to remove them from the seas after they have become dispersed.
8. **Control of Ocean Pollution Will Advance the Sustainable Development Goals (SDGs).** All actions taken to control and prevent pollution of the oceans will advance attainment of multiple SDGs.
  - Most directly, control of ocean pollution will advance SDG 14, which calls on all countries to “prevent and significantly reduce marine pollution of all kinds, in particular from land-based activities, including marine debris and nutrient pollution” by 2025.
  - Control of ocean pollution will advance SDG 3, which calls for improvement of human health and well-being;
  - Additionally, control of ocean pollution will advance:
    - SDG1, which calls for an end to poverty;
    - SDG2, which calls for an end to hunger;
    - SDG 6, which calls for clean water and sanitation;
    - SDG 8, which calls for decent work and sustainable economic growth; and
    - SDG12, which calls for responsible consumption and production.

## Recommendations – The Way Forward

### Policy Priorities

- **Prevent Mercury Pollution of the Oceans.** Two actions will be key to preventing further addition of mercury to the oceans. These are:
  1. Cessation of coal combustion; and
  2. Control of inorganic mercury, especially in artisanal and small-scale gold mining (ASGM).
  Cessation of coal combustion will not only slow the pace of climate change and reduce particulate air pollution, but will also greatly reduce the atmospheric emissions of mercury, thus reducing deposition of mercury into the oceans. Actions ongoing under the Minamata Convention on Mercury are seeking to identify and control major sources of mercury pollution [34].
- **End Plastic Pollution of the Oceans and Consider a Global Ban on Production of Single-Use Plastic.** Marine plastic pollution has become one of three top priorities in global pollution identified by UN Environment [581]. Many countries have taken regulatory and social actions to control the use and disposal of plastics and reduce plastic waste. These include bans of single-use articles such as plastic bags and straws and bans on the use of cosmetic microbeads. In 2015, the United States banned the manufacture and distribution of cosmetic products containing plastic microbeads. The EU parliament has voted to ban several single-use plastic categories (cutlery, cotton buds, straws and stirrers) by 2021.
- **Promote Effective Waste Management.** Improvement in collection and management of solid waste is a key strategy for prevention and control of marine plastic pollution. UNESCO reports that seven of the EU Member States plus Norway and Switzerland now recover more than 80% of their used plastics. These countries have adopted integrated waste and resource management strategies to address each waste stream with the best options. Rwanda, Kenya, and some jurisdictions in the United Sates have banned single-use plastic bags. These are model programs and have potential to extend to other countries.
- **Reduce Releases of Nitrogen, Phosphorus, Animal Waste, Industrial Discharges and Human Sewage into Coastal Waters.** Proper management of coastal pollution can reduce the frequency of HABs, prevent eutrophication, and alleviate the burden of disease associated with HABs and marine pathogens. Monitoring seafood, including farmed fish, for human pathogens is a proven strategy for tracking the efficacy these control efforts and reducing risk of disease. The UNESCO Blueprint for Ocean and Coastal Sustainability includes proposals to green the nutrient economy and achieve these goals [578].
- **Create Marine Protected Areas.** Designation of new Marine Protected Areas around the world will safeguard critical ecosystems, protect vulnerable fish stocks, and enhance human health and well-being [586–588]. Creation of Marine Protected Areas is an important manifestation of national and international commitment to protecting the health of the seas.
- **Support Robust Monitoring of Ocean Pollution.** To safeguard human health in all countries against pollutants in the oceans and to protect consumers against pollutants in seafood, pollutant monitoring programs and monitoring capacity need to be extended worldwide. Specific needs are the following:
  - Assist countries with the establishment and certification of monitoring programs for chemical pollutants, algal toxins, microplastics, and microbial pathogens in seafood products.
  - Build and sustain strong transdisciplinary teams of scientists and strengthen analytical capabilities at the national level to provide countries with capability to respond to new and unexpected marine pollutants.
  - Develop new monitoring capabilities using networks of *in situ* sensors that can detect toxic chemical pollutants, HAB cells and their toxins, microplastics and pathogenic bacteria.
  - Support the global efforts of the IOC-UNESCO Intergovernmental Panel on Harmful Algal Blooms (IPHAB) [389].
  - Enhance communication, literacy and outreach efforts so that the risks of human illness and death from ocean pollutants is recognized and understood throughout all levels of society.
- **Extend Regional and International Marine Pollution Control Programs to all Countries.** A number of regional and international pollution control strategies have been developed and implemented in recent decades. (See **Text Box 16**). These policies recognize the reality that pollution of the oceans transcends national boundaries and that mitigation must therefore involve not only efforts within countries, but also transnational, regional and even global efforts. Effective monitoring strategies in support of these programs need to link ecological and human health data, and not keep these two streams of information separate [582,583]. In the years ahead it will be important that these beneficial programs be extended to all countries and that they be adequately funded by national governments and international organizations [12].
  **TEXT BOX 16: Regional and International Marine Pollution Control Programs.**
  - The London Convention on the Prevention of Marine Pollution by Dumping of Wastes and Other Matter (1975) and its Protocol (1996)
  - The United Nations Convention on the Law of the Sea (1982)
  - The OSPAR Convention for the Protection of the Marine Environment of the North-East Atlantic (1992)
  - The Bucharest Convention on the Protection of the Black Sea against Pollution (1992)
  - The Helsinki Convention on the Protection of the Marine Environment of the Baltic Sea Area (1992) and its Action Plan (2007)
  - The Barcelona Convention for the Protection of the Marine Environment and the Coastal Region of the Mediterranean (1995) and its Protocols (2005)
  - The Stockholm Convention on Persistent Organic Pollutants (2001)
  - The Strategic Action Plan for the Environmental Protection and Rehabilitation of the Black Sea (2009)
  - The Minamata Convention on Mercury (2013)
  - The United Nations Decade of Ocean Science for Sustainable Development (2021–2030)
- **Ultimately, prevention and control of ocean pollution can be achieved by transition to a circular, more efficient, less wasteful economy and embracing the precepts of green chemistry** [**572**,**584**]. This is a high-level strategy that will take years to accomplish. It is, however, an essential requirement for the prevention of ocean pollution and mitigation of global climate change (See **Text Boxes 14** and **15**).
  **TEXT BOX 14: *Principles of a Circular Economy***
  In a circular economy, economic, and social development is decoupled from the consumption of non-renewable resources. The generation of pollution and other forms of waste is minimized and replaced by recycling and reuse [2]. The focus is on stability and equity rather than endless growth.
  The core principles of a circular economy are preservation of natural capital by reducing use of non-renewable resources and ecosystem management; optimization of resource yields by circulating products and materials so that they are shared and their lifecycles extended; and fostering system effectiveness by designing out pollution, greenhouse gas emissions, and toxic materials that damage health [2].
  Evidence of global movement towards a circular economy is seen in policy-related recommendations to control plastic pollution of the oceans that have been proposed by the UN Food and Agriculture Organization (FAO) and the Group of Experts on the Scientific Aspects of Marine Environmental Protection (GESAMP). These bold and visionary strategies call for sweeping change in current, highly wasteful practices of plastic production and consumption and for a global move toward biodegradable, non-persistent polymers [572]. They provide a model for interventions against other marine pollutants.
  **TEXT BOX 15: *The Promise of Green Chemistry***
  Green chemistry is “the design of chemical products and processes to reduce and eliminate the use and generation of hazardous compounds” [573].
  Adoption of the principles of green chemistry will require a paradigm shift away from narrow consideration of the properties and economic viability of new molecules and chemical products towards consideration and avoidance of their potential negative impacts on humans, ecosystems, and society. This reorientation will need to take place in every stage in the design and development of new chemicals and new chemical products from their earliest inception.
  Green chemistry takes special note of the potential of new chemicals to cause low-dose toxicity through mechanisms such as endocrine disruption and developmental toxicity, and it avoids new products that will persist in the environment or in living organisms. The goal is to create safe, nontoxic materials and technologies and thus prevent future health and environmental catastrophes while building a sustainable chemical economy [574].
  Wide-scale adoption of the principles and practices of green chemistry coupled with broad movement towards a circular economy could reduce pollution of the world’s oceans by manufactured chemicals and plastic waste and end the need to balance the dangers of toxic chemicals in seafood against the clear benefits of seafood for human health.

### Research Priorities

The overall goal of the following research recommendations is to increase knowledge of the extent, severity, and human health impacts of ocean pollution. A second goal is to better quantify the contributions of ocean pollution to the global burden of disease (GBD). Findings from the GBD study have become powerful shapers of health and environmental policy and are used by international agencies and national governments to set health and environmental priorities and guide the allocation of resources. It is therefore critically important that accurate information on the disease burden attributable to ocean pollution be accurately and fully captured in the GBD analysis and made available to policy-makers. Specific recommendations are the following:

- **Improved mapping of ocean pollution and its health impacts.** A major impediment to estimating the GBD attributable to pollution of the oceans is a lack of comprehensive, geospatially coded measurements that display current information on the types and concentrations of pollution in seas around the world and their impacts on human health and well-being. Absent this information, it is not possible to estimate the sizes of the populations exposed to ocean pollutants or their levels of exposure. Opportunity exists here to apply new technologies such as satellite imaging and ocean sampling by marine saildrones and autonomous underwater vehicles coupled with big data analyses that integrate data from multiple sources.
  Monitoring for all of the chemical and biological hazards in the oceans should increase in scope and be coordinated globally. It is possible to monitor for some biological hazards, ocean pH, and temperature in sensors that are part of the Global Ocean Observing system (GOOS) within the UN system. Enhancing this capability and adding sensors for chemical hazards that incorporate new technologies and capabilities is an objective that may be achieved by partnering with programs such as the Partnership for Observation of the Global Ocean (POGO).
- **Enhanced sampling of pollutant concentrations in fish, shellfish, and marine mammals.** Because consumption of contaminated seafood is the major route by which chemical pollutants in the ocean as well as HAB toxins reach humans, better information is needed on concentrations of key pollutants in seafood. High-quality data are available from high-income countries, but much less information is available from the countries of the Global South.
- **Improved tracking of biomarkers that are early indicators of damage caused to human health and marine ecosystems by chemical pollutants.**
- **Expanded coverage of ocean sampling for marine pathogens.** Techniques have been developed for monitoring the global spread of pathogenic bacteria, such as *Vibrio* species, but these techniques have been deployed to date in only a few areas of the world. Expanded geographic coverage of marine bacterial sampling – especially into areas important for commercial fishing, shellfish harvesting and aquaculture – coupled with real-time information on sea surface temperature will be important for tracking, and predicting the spread of life-threatening bacteria and for mobilizing early responses to new outbreaks of diseases.
- **Improved studies of human exposure to ocean pollutants.** A major impediment to developing estimates of the GBD attributable to ocean pollutants is lack of detailed, population-level studies of human exposures to marine pollutants. Conducting such studies in a number of countries will elucidate the importance of such factors as geographic variation in background exposure to pollutants, in seafood consumption, in pollutant concentrations in seafood, and in exposures to toxic chemicals via routes other than consumption of contaminated seafood.
- **Improved assessments of combined effects of exposures to multiple ocean pollutants.** Humans are seldom exposed to pollutants one at a time. Instead, people are typically exposed to complex mixtures of pollutants. The limited available evidence indicates that these combined exposures can produce additive, synergistic, and antagonistic effects.
- **Implementation research.** Transdisciplinary international cooperative implementation research is needed to identify best practices and feasible, cost-effective solutions to prevention and control of ocean pollution. This research will build upon and codify the findings that have emerged from the case studies in success against ocean pollution presented in this report. Continuing research and development into biodegradable polymers will be an important component of this research [572].
- **Enhanced capacity in ocean research and monitoring.** The building of professional capacity in all countries will be of great importance to safeguarding human health against ocean pollution and its health consequences. Key elements of building professional and scientific capacity building are:
  - Build and sustain strong teams of scientists at the national level to provide each country with capacity to respond to new and unexpected marine pollutants and assess their health impacts.
  - Establish lines of communication and collaboration between marine scientists and public health agencies and institutions [531].
  - Support the global efforts of the IOC-UNESCO Intergovernmental Panel on Harmful Algal Blooms (IPHAB), which coordinates actions at a policy level and that relies on the work of institutions in many countries, and contributes to achieve the SDGs.
  - Develop new monitoring capabilities using networks of *in situ* sensors that can detect toxic chemical pollutants, HAB cells and their toxins, microplastics, and pathogenic bacteria.
  - Deploy improved analytical capabilities to document health and economic benefits of programs to control and prevent ocean pollution.
  - Assist countries with the establishment and certification of monitoring programs for chemical pollutants, algal toxins, microplastics, and pathogenic bacteria in seafood products.
  - Strengthen analytical capabilities at the national level.
  - Support research and application of technologies for control of marine pollutants.
  - Enhance communication, literacy and outreach efforts so that the risks of human illness and death from ocean pollutants is recognized and understood throughout all levels of society.

## Additional Files

The additional files for this article can be found as follows:

10.5334/aogh.2831.s1 Supplementary Appendix. This Supplementary Appendix contains additional references and documentation supporting the information presented in the report, Human Health and Ocean Pollution.

10.5334/aogh.2831.s2 Declaration of Monaco. This Declaration summarizes the key findings and conclusions of the Monaco Commission on Human Health and Ocean Pollution. It is based on the recognition that all life on Earth depends on the health of the seas. It presents a Call to Action – an urgent message addressed to leaders in all countries and to all citizens of Earth urging us to safeguard human health and preserve our Common Home by acting now to end pollution of the ocean.
