# Supplementary material for: Human Health and Ocean Pollution
Source: Ann Glob Health. 2020 Dec 3;86(1):151. doi: 10.5334/aogh.2831 (PMC7731724; doi:10.5334/aogh.2831)
Supplement: Supplementary Appendix. — This Supplementary Appendix contains additional references and documentation supporting the information presented in the report, Human Health and Ocean Pollution. [file agh-86-1-2831-s1.pdf]

Supplementary Appendix to "Human Health and Ocean Pollution", *Annals of Global Health,* 2020

This Supplementary Appendix contains additional references and documentation supporting the information presented in the report, Human Health and Ocean Pollution.

Chemical Pollution of the Oceans

Toxic chemical pollutants in the oceans have been shown capable of causing a wide range of human diseases. Toxicological and epidemiological studies document that pollutants such as toxic metals, POPs, dioxins, plastics chemicals, and pesticides can cause cardiovascular effects, developmental and neurobehavioral disorders, metabolic disease, endocrine disruption and cancer.

Table 1 in this **Supplementary Appendix** summarizes the known links between chemical pollutants in the oceans and a range of human health outcomes. The strengths of the associations listed in Table 1 vary depending on the nature of the studies establishing these associations. Some associations have been assessed in systematic reviews and meta-analyses of animal and human data.[1] [2] Some are single cross-sectional or case-control studies. There are now a growing number of relevant epidemiological studies, including powerful prospective cohort studies, such as the Nurses' Health Study II and the Prospective Investigation of the Vasculature in Uppsala Seniors (PIVUS)[3] Findings from these investigations are strengthening the evidence base for associations between exposures to organic chemical pollutants and adverse health outcomes.

Supplementary Appendix Table 1. Adverse Human Health Outcomes Linked to Chemical Pollutants in the Oceans, Including Endocrine Disrupting Chemicals

Adverse outcome	Pollutants associated	Types of Evidence	Types of epidemiological studies	Strength of Evidence
Cardiovascular<br>Disease, including hypertension, stroke and increased mortality,	• *PCBs, dioxins,<br>• BPS<br>• PBDEs<br>• Organophosphates<br>• Organochlorines	Epidemiology;<br>Toxicology	Geographic; case-control; cross-sectional;<br>Prospective cohort	Moderate
Impaired somatic development (growth and birth weight)	• POA,<br>• *PCBs<br>• Lead	Epidemiology	Cross-sectional;<br>Prospective cohort	Strong
Developmental Neurotoxicity, including Decreased IQ, learning	• Lead<br>• *PCBs<br>• Methylmercury	Epidemiology;<br>Toxicology	Systematic reviews,<br>Prospective	Strong

disabilities, conduct disorder, ADHD and Autism spectrum disorder (ASD)	• Organophosphates<br>• Organochlorines<br>• BPA<br>• Phthalates<br>• Phosphorylated and polybrominated flame retardants (PBDEs)<br>• Perchlorate (through thyroid impairment)<br>• Perfluorinated compounds (PFAS)<br>• Fine particulate air pollution ($PM_{2.5}$)		cohort	
Adult neurotoxicity, with cognitive and motor impairment	• Methylmercury<br>• Lead	Epidemiology	Cross-sectional, cohort	Strong
Endocrine effects:<br>Steroid and thyroid	• *PCBs<br>• DDT<br>• PBDEs	Epidemiology;<br>Toxicology	Cross-sectional	Moderate
Immune system effects	• *Dioxins<br>• DLC	Epidemiology;<br>Toxicology	Cross-sectional	Moderate
Metabolic disorder, including hyperlipidemia, insulin resistance, obesity and type 2 diabetes	• PFAS<br>• HCB<br>• *PCBs<br>• *Dioxins<br>• Phthalates,<br>• Polycyclic Aromatic Hydrocarbons (PAH)<br>• DES<br>• Tributyl tin	Epidemiology;<br>Toxicology	Cross-sectional,<br>Prospective cohort	Weak to moderate for early-life exposures to pesticides, PCBs, phthalates, dioxins, and polycyclic aromatic hydrocarbons (PAH) Strong experimental evidence for BPA<br>Strong for DESand tributyl tin
Male reproductive effects, including testicular dysgenesis syndrome,[4] Cryptorchidism,	• Phthalates<br>• BPA<br>• DDE<br>• PCB	Epidemiology;<br>Toxicology	Cross-sectional,<br>Prospective cohort	Moderate/Strong for phthalates and BPA<br>Moderate for DDE and PCB

Hypospadias, Decreased ano-genital distance and Decreased male fertility				
Female reproductive effects, including early onset of puberty, polycystic ovary syndrome (PCOS), decreased ovarian reserve; increased time to pregnancy; decreased fertility and endometriosis	• Low dose BPA<br>• Prenatal exposure to methoxychlor resulting in impaired ovarian reserve<br>• Prenatal exposure to high doses of DDT	Epidemiology;<br>Toxicology	Cross-sectional,<br>Clinical	Weak for BPA<br>Strong for methoxychlor, DDT and Dioxin
Female Breast Cancer	• DDT<br>• PCBs<br>• Dioxin<br>• BPA	Epidemiology;<br>Toxicology	Cross-sectional,<br>Retrospective cohort	Strong for prenatal exposure to DDT and PCBs.<br>Strong for peripubertal exposure to dioxin<br>Experimental evidence only for prenatal exposure to BPA
Prostate Cancer	• Chlordecone, an organochlorine pesticide,<br>• BPA	Epidemiology;<br>Toxicology	Cross-sectional	Weak for chlordecone<br>Moderate for BPA
Testicular Germ Cell Cancer	• Organochlorine pesticides	Epidemiology	Prospective cohort	Weak for prenatal exposure to organochlorine pesticides

*Congeners may differ in their effects

Endocrine Disruption – A Newly Recognized Mechanism of Chemical Toxicity

Classic toxicological teaching dating from the 16th century holds that "the dose makes the poison", i.e., the greater the exposure to a harmful material, the more severe and frequent are the resulting toxic effects. Accordingly, conventional safety testing of chemicals has assumed that high-dose testing would reveal all important adverse effects, and that these high-dose findings could be extrapolated down to low doses, based on a dose-response relationship, to identify a point at which no effect would be seen (called the "No Observed Adverse Effect Level, or NOAEL). The NOAEL has provided the starting point for setting legal standards for chemicals. Safety factors are applied to the NOAEL to calculate what has been assumed to be a safe exposure level, usually 1000-fold beneath the NOAEL. This strategy has been used to test chemicals for their lethal, carcinogenic, mutagenic reproductive and developmental effects.

While the dose-response relationship remains a core concept in toxicology, two fundamentally new insights that have emerged in the past twenty years have disrupted and expanded this classic paradigm. These are the concepts of **endocrine toxicity**[5,6] and of **developmental toxicity**. In both of these new constructs, even very small exposures to manufactured chemicals – exposures in the parts-per-billion range that were previously thought to be safe - have been shown capable of causing profound disruptions in organ systems that can lead to disease, disability and death. Some of these compounds, such as bisphenol A (BPA), may act at such low environmentally relevant doses that for many years they were not assessed in standard toxicological programs.[7]

Table 1 (above) in this **Supplementary Appendix** presents a summary of the human health effects that are known to be mediated through endocrine disruption. These effects include disruption of early somatic development as well as of neurobehavioral development by exposures incurred prenatally; hypospadias; cryptorchidism; testicular cancer; the metabolic syndrome, obesity and diabetes; impaired fertility in both males and females; and cancer.

Marine Algae and Harmful Algal Blooms (HABs)

The smaller, microscopic algal species in the oceans that produce toxins and cause HABs include *diatoms, dinoflagellates, coccolithophorids,* and *cyanobacteria* (also termed blue-green algae). Remarkable progress has been achieved in recent decades in refining the taxonomy of these species and in developing new technologies for detection and quantification of HAB cells and their toxins.[5] (**Supplementary** Appendix **Table 2**)

Most algal toxins can be produced by several species of algae, sometimes even several genera from very different habitats or lineages. This is the case for example of okadaic acid which is produced by several species of *Dinophysis (planktonic)*, and of *Prorocentrum (benthic)*. Also some species of al;gae may produce several different toxins (e.g., *Alexandrium ostenfeldii* can produce saxitoxins and spirolides). These complexities complicates risk assessment and interfere with the determination of the specific toxins responsible for disease outbreaks.

Some algal toxins, including saxitoxins, tetrodotoxins, and domoic acid, are highly soluble in water, and can result in death within fifteen minutes of their consumption in seafood.[8] Because of their very high water solubility, saxitoxins and domoic acid are not biomagnified in seafood, but merely accumulate through the intake of algae. Other algal toxins such as ciguatoxins are lipophilic, tend to reside for longer times in seafood and may reach high concentrations in carnivorous top predator fish such as barracuda.[6,7]

Because of their high potency, marine toxins can cause disease even at relatively low levels of exposure. Clear waters are therefore no guarantee that seafood is safe. Also, these toxins have no color, taste or smell and thus cannot be detected by visual or olfactory inspection of seafood. All toxins described in this section are stable up to 100°C, and therefore are not destroyed by cooking. [9]

Because most algal toxins are soluble to some degree in seawater, they can be detected through the use of passive seawater samplers (e.g., SPATTS).[10] Many analytical methods for testing algal toxins in seafood are now available, and they support monitoring programs to protect human health. Less is

known about the toxicity of macroalgal HABs such as the massive outbreaks caused by *Ulva* and *Sargassum*.[11,12]

<u>Supplementary Appendix Table 2</u>. Marine and estuarine HAB species, their toxins, mechanisms of action and health effects [13]

	**Causative organism (genera or species)**	**Group toxins and other mechanisms**	**ARfD**	**Syndromes and other effects**
Microalgae producing toxins controlled for seafood safety	*Amphidoma languida, Azadinium poporum, A. spinosum, A. dexteroporum*	*Tox*: Azaspiracids (**AZA**)	0.2 µg AZA1 eq/Kg b.w (EFSA)<br>0.04 µg/kg b.w. (CODEX)	*Food Ill.* **DSP (aka AZP** *azaspiracid shellfish poisoning)*<br>Gastrointestinal
	Karenia brevis, K. papilionacea	*Tox*: Brevetoxins (**BTX**)<br>*Biomass*: hypoxia/anoxia	N/A	*Food Ill.* **NSP (***neurotoxic shellfish poisoning)*<br>*Gastrointestinal*<br>*Neurologic*<br>*Resp Ill.*<br>*Marine die-offs* **(**FKT)
	Gambierdiscus australes, G. balechii, G. belizeanus, G. caribaeus, G. carolinianus, G. carpenteri, G. cheloniae, G. excentricus, G. honu, G. jejuensis, G. lapillus, G. pacificus, G. polynesiensis, G. scabrosus, G. silvae, G. toxicus, G. yasumotoi, Fukuyoa paulensis, F. ruetzleri, F. yasumotoi	*Tox:* Ciguatoxins (CTX)<br>Maitotoxins (MTX)	N/A	*Food Ill.* **CP** *(ciguatera poisoning)*<br>Gastrointestinal<br>Neurologic<br>Cardiovascular<br>Neuropsychiatric<br>n.e.p.
	Halamphora coffeaeformis, Nitzschia bizertensis, Nitzschia navis-varingica<br>*Pseudo-nitzschia australis, P. brasiliana , P. calliantha, P. cuspidata, P. delicatissima, P. fraudulenta, P. galaxiae, P. granii, P. multiseries, P. multistriata, P. pseudodelicatissima, P. pungens, P. seriata, P. turgidula,*	*Tox*: Domoic acid (**DA**)	30 µg DA eq/Kg b.w (EFSA)<br>100 µg/kg b.w. (CODEX)	*Food Ill.* **ASP** *(Amnesic shellfish poisoning)*<br>Gastrointestinal<br>Neurologic
	Dinophysis acuminata, D. acuta, D. caudata, D. fortii, D. norvegica, D. ovum, D. sacculus, D. miles<br>*Phalacroma rotundatum, P. rapa, P. mitra, Prorocentrum belizeanum, P. concavum, P. faustiae, P. hoffmanianum, Prorocentrum leve, P. texanum, P.*	*Tox*: Okadaic acid (**OA**) and *Dinophysis* toxins<br>Tox: Pectenotoxins (PTX)	0.3 µg OA eq/Kg b.w. (CODEX and EFSA)<br>0.8 µg PTX2 eq/Kg	*Food Ill.* **DSP** *(Diarrhetic shellfish poisoning)*<br>Gastrointestinal<br>n.e.p.

	maculosum[§], *P. rhathymum, P. Lima*	Tox: Prorocentrolides	b.w. (EFSA)	
	Ostreopsis lenticularis, O. mascarenensis, Ostreopsis ovata, O. cf. *ovata, O. siamensis, Trichodesmium erythraeum*	*Tox*: Palytoxins (**PLTX**) :<br>Mascarenotoxins, Ovatoxins and Ostreocins	0.2 µg PlTX eq/kg b.w. (EFSA)	*Resp Ill.*<br>*Food Ill:* **CPT** *(clupeotoxism)*<br>*Gastrointestinal*<br>*neurologic*<br>*Cardiovascular*
	Alexandrium affine, A. ostenfeldii, A. acatenella , A. catenella, A. cohorticula, A. peruvianum, A. tamiyavanichii, A. andersonii, A. fundyense, A. tamarense, A. leei, A. minutum, Gymnodinium catenatum, Pyrodinium bahamense, Trichodesmium erythraeum	*Tox:* Saxitoxins (**STX**)	0.5 µg STX eq/Kg b.w (EFSA)<br>0.7 µg STX eq/Kg b.w (CODEX)	*Food Ill:* **PSP** Paralytic Shellfish poisoning<br>*Marine die-offs* (FKT)
	Protoceratium reticulatum, Lingulodinium polyedra, Gonyaulax spinifera*	*Tox:* Yessotoxins (**YTX**)<br>Adriatoxin	25 µg YTX eq/Kg b.w (EFSA)<br>50 µg YTX eq/Kg b.w	n.e.p.<br>*in mice: neurological, cardiovascular, hepatic*
Other microalgae	*Alexandrium ostenfeldii, A. peruvianum*	Toxins: Spirolides	N/A	n.e.p.
	Alexandrium hiranoi, A. monilatum, A. Pseudogonyaulax	Toxins: Goniodomine A		Marine die-offs (FKT)
	Coolia malayensis, Coolia monotis, Coolia tropicalis	Toxins: Cooliatoxin	N/A	*n.e.p.*
	Heterocapsa triquetra	ß-methyl-amino alanine	N/A	Food Ill. *Neurologic*
	Karenia mikimotoi	Toxins: Gymnocins	N/A	Marine die-offs (FKT)
	Karenia selliformis, Alexandrium peruvianum	Toxins: Gymnodimines	N/A	n.e.p.
	Karenia brevisulcata	Brevesulcenals	N/A	Resp Ill.<br>Marine die-offs FKT
	Karenia breviulcata	Toxins: Karenia brevisulcata toxins	N/A	Resp Ill.<br>Marine die-offs FKT
	Karlodinium veneficum, K. conicum	Toxins: Karlotoxins	N/A	Marine die-offs (FKT)
	Vulcanodinium rugosum	Toxins: Pinnatoxins	N/A	n.e.p.
cyanob	*Lynbya majuscula*	Toxins: Lyngbyatoxins, antillatoxins, aplysiatoxins,	N/A	**TSD** *(toxic seaweed dermatitis)*

		barbamides, curacins, kalkitoxins, kalkipyrone, hermitamides, manauealides		*Dermatological*
	Microcystis spp<br>*Anabaena spp*<br>*Nostoc spp*	Toxins: Microcystins	N/A	**HPT**<br>*Hepatic*<br>*Gastrointestinal*<br>*Allergy, irritation*
	Nodularia spumigena	Toxins: Nodularins	N/A	**HPT**<br>*Hepatic*
	Prymnesium parvum	Toxins: Prymnesins	N/A	Marine die-offs (FKT)
	Rivularia sp.	Toxins: Viequeamides (=Kulolides)	N/A	n.e.p.
Macro-Algae	*Chondria armata*	Domoic acid	N/A	
	Laurencia intricata, Spyridia filamentosa, Dictyota species, Enteromorpha species, *Codium isthmocladum,*<br>*Halimeda* species, Caulerpa species, *Codium isthmocladum*	Biomass, hypoxia and anoxia	N/A	Marine die-offs emigration of reef fishes
	Ulva prolifera, Sargassum filipendula	Biomass, hypoxia and anoxia, H_2S	N/A	

**The table reports the harmful species, their harmful mechanism (Tox: Toxin group; biomass: hypoxia, anoxia), the corresponding direct harmful effect to human (foodborne, waterborne or airborne illnesses, respectively Food Ill, Water Ill and Resp Ill), and to fish or shellfish (marine die-offs). Abbreviations: FKT (fish killing toxins), HPT: hepato-toxicity, n.e.p. = no effect proven in human, ARfD Acute reference dose (amount that can be ingested in a period of 24hr); b.w. body weight. Acute reference doses (ARfD) have been derived for HAB toxins from lowest- or no-observed adverse effect levels observed in animal species.

Bacterial Pathogens in the Oceans

Bacterial Survival Strategies in a Changing Marine Environment

Bacterial pathogens have high capacity to adapt to changing environments. Adaptive strategies used by marine bacteria include the following:

- Horizontal gene transfer (HGT). In HGT, genes are exchanged between bacteria and also between bacteria and other marine microorganisms such as viruses through the processes of conjugation, transduction, and transformation. Bacterial genomes are rich in mobile, transferrable genetic elements such as self-transmissable plasmids, transposable elements, and temperate bacteriophages.[14] HGT allows bacteria to acquire new genetic material [15] and develop new traits.
- Production of adhesion molecules. Bacteria can produce adhesive molecules and structures on their outer surfaces that allow them to attach to plastic particles and other pollutants in the ocean,[16] thus

aiding their dispersal through the action of tides and currents.[17] The ability to make physical attachments also aids bacteria in colonizing marine organisms, particularly phytoplankton, thereby augmenting HGT and increasing access to nutrients.

- Biofilm production. Bacteria in in marine and coastal environments can produce biofilms that enable them to resist dispersal by tides and currents and to mitigate the effects of chemical and UV stress as well as the effects of antimicrobial agents.

These adaptive properties enable bacteria to disrupt ecosystems and cause disease. For example, indigenous marine bacteria can gain genetic material from allochthonous bacteria introduced into the oceans from land-based sources and thereby acquire anti-microbial resistance and increased virulence.[18,19] Bacteria with acquired virulence factors have caused disease in humans and have disrupted aquaculture by causing infections among farmed fish causing substantial economic losses.[20]

Tracking bacterial pathogens and their evolutionary strategies will be a major focal point of research in the coming decade. Information gained from this research will have great relevance to both ecosystem health and human health.

Successes in Prevention and Control of Ocean Pollution

A key finding of the 2018 *Lancet* Commission on Pollution and Health is that much pollution can be controlled and pollution-related disease prevented.[21] The Commission noted that most high-income countries and an increasing number of middle-income countries have curbed their most flagrant forms of pollution by enacting environmental legislation and developing regulations.

The strategies used to control pollution of air and water have been applied successfully to the prevention and control of ocean pollution. Key to the effectiveness of these efforts has been the recognition that 80% of ocean pollution arises from land-based sources. Accordingly, these programs have identified, targeted, and reduced releases from their most important land-based polluters. They have been guided by multi-scale monitoring that tracks pollutant discharges, measures pollutant levels in the seas and in marine biota, and assesses human exposure and health outcomes. They have been backed by strict enforcement. They have engaged civil society and the public by making their strategies, their data, and their progress reports available on open-source platforms.

This following Text Boxes present case studies of success in control of ocean pollution. A central element in each of these examples has been careful documentation of progress against pollution through robust monitoring.

SUPPLEMENTARY APPENDIX TEXT BOX 1. Using Seagrass Meadows to Mitigate Pathogen Pollution
Seagrass meadows are found along the coasts of all continents except Antarctica, and are considered the world's third most valuable ecosystem. Seagrass meadows are critical for coastal protection, they serve as nurseries for commercially relevant seafood species, and they sequester significant amounts of carbon thus combating climate change and ocean acidification. Seagrass meadows can provide additional ecosystem services by contributing to control of microbial contamination of the oceans.

A recent study found that seagrass meadows can reduce the abundance of bacterial pathogens capable of causing disease in humans and marine organisms by 50% and at the same time improve the health of nearby coral reef ecosystems.[22] A further benefit is taht yields of agarophyte farming for the production of agar for use in the cosmetics industry are up to 25% higher when the algae are grown in seagrass meadows due to a significant reduction in disease levels.

The capacity of seagrass meadows to eliminate pathogens and mitigate disease in nearby areas shows their potential as a natural filtration system that may be applied to clean up waste water and improve the health of organisms in the aquaculture and mariculture industries.

Despite these enormous ecological, economical and human health benefits, the global surface cover of seagrass is declining each year. Conservation and restoration of seagrass ecosystems is therefore urgently needed to sustainably reduce ocean pollution and improve the health and livelihoods of local human populations.

SUPPLEMENTARY APPENDIX TEXT BOX 2. Reduction in Harmful Algal Blooms (HABs) in the Black Sea through Reductions in Fertilizer Use

A striking, though unplanned example of the impact of fertilizer use on HAB incidence is seen in the case of the northwestern Black Sea.

In the 1960s, algal blooms were rare in the Black Sea**.** However, in the 1970s and '80s heavy pollution loading with nitrogen- and phosphorus-based fertilizers occurred in the eight countries within the Black Sea watershed. This was the result of the Soviet government's provision of economic subsidies for chemical fertilizers; the government's goal was to sharply increase agricultural production in the region.[27] A consequence of this great increase in fertilizer use and the subsequent increase in coastal runoff of nutrients was a striking increase in eutrophication of the Black Sea and in the frequency and magnitude of algal blooms, which became recurrent with cell densities greatly exceeding past abundance levels. Decreased abundance of diatoms and larger algae and their replacement by flagellates and nanoplankton was also noted.

In a striking reversal, algal blooms began to decrease in 1991, both in number and in size. Diatoms became more dominant, and abundances of nanoplankton and flagellates decreased. These changes coincided with significant decreases in use of chemical fertilizer that were the consequence of the reductions in economic subsidies that accompanied the breakup of the former Soviet Union. [28]

While this chain of events was the result of unplanned political disruption and not the consequence of a deliberate intervention, it nonetheless provides a clear illustration of the link between some HAB events and coastal pollution.

SUPPLEMENTARY APPENDIX TEXT BOX 3. Pollution Clean-Up in Hong Kong's Victoria Harbour. A Success story

Background. Victoria Harbour is a deep natural harbor that separates Hong Kong Island from the Kowloon Peninsula. It lies at the center of Hong Kong has been key to the city's rise as a trading center and global metropolis. In the 1960s, major land reclamation projects were begun on the shores of Victoria Harbour, and by 1970 almost the entire coastline had been filled and the shoreline extended. This large expansion of Hong Kong's landmass decreased tidal flushing while at the same time rapid industrialization and population growth resulted in increased industrial effluents and led to the daily dumping into Victoria Harbour of several hundred tons of untreated sewage.[29] Oil spills from marine traffic further degraded the environment. The harbor became highly polluted and unsafe for swimming.

Solution. In 1989, the Strategic Sewage Disposal Scheme (SSDS) for Victoria Harbour was launched and later renamed the Harbor Area Treatment Scheme (HATS). This was a multi-stage pollution control plan intended to decrease pollution levels, allow re-opening of beaches and coastal promenades to swimming and recreation, and improve public health and safety. HATS Stage 1 commenced in 1994 and was designed to chemically treat sewage from Kowloon, Kwai Tsing, Tseung Kwan O, and Northeastern Hong Kong islands prior to discharge into the harbor. The main elements of Stage 1 were the construction of a sewage tunnel network, upgrading of seven Preliminary Treatment Works (PTWs), construction of the Stonecutters Island Sewage Treatment Works (SCITW), and construction of a tunnel and pipeline in the southwest of Stonecutters Island. HATS Stage 2 commenced in 2001 and Phase 2A in December 2015.[29] Its goal was to treat the last 25% of sewage from the northern and southwestern parts of Hong Kong. This involved upgrading of the PTWS and the SCISTW as well as construction of a deep tunnel that transferred sewage from PTWs for secondary treatment. A disinfection facility was built to remove 99% of *E.coli* from sewage. HATS Stage 2B will be the next phase of the project to be launched.[29] It will have the goal of biologically treating all effluent discharged into Victoria Harbour.

Results. Stage 1 resulted in a 10% increase of dissolved oxygen levels in Victoria Harbour; decreases in concentrations of major pollutants; and reductions in concentrations of ammonia by 25%, inorganic nitrogen by 16%, inorganic phosphorus by 36%, and *E.coli* by 50%. Phase 2A built on these advances.[29] It resulted in Victoria Harbour meeting its water quality objectives, which in turn led to improvements in the health of the marine environment and to re-opening of the harbor beaches.[30]

Conclusion. Hong Kong has made great progress in cleaning up Victoria Harbour, but there is still further work needed to achieve full restoration

SUPPLEMENTARY APPENDIX TEXT BOX 4. Saving Chesapeake Bay

Background. Chesapeake Bay, situated on the US East Coast between the states of Maryland and Virginia is the largest estuary in the U.S. The Bay is home to a variety of underwater plants that guard shorelines against erosion and storms, store carbon, and provide sustenance and shelter for multiple marine species. It is estimated that these plants provide trillions of dollars in "ecosystem services" to society each year.[38]

From the 1950s to the 1970s, agriculture and urbanization threatened the Bay's plants through increasing coastal pollution with nitrogen and phosphorus. These pollutants fueled the growth of algae

that prevented light from reaching the plants.[39] Consequently, tens of thousands of acres of underwater plants disappeared, representing the Bay's largest decline in over four centuries.[40]

Solution. The Clean Water Act led to establishment of a "pollution diet" for the Chesapeake Bay. The act requires the identification of waterways impaired by pollutants, as well as the development of Total Maximum Daily Loads (TMDLs) for these waterways (TMDLs are "pollution diets" that establish the maximum amount of a pollutant that can enter a waterway each day). Beginning in 2000, the seven jurisdictions in the Chesapeake Bay watershed, the Environmental Protection Agency, and the Chesapeake Bay Commission formulated a Chesapeake Bay TMDL to counter the negative effects of nitrogen and phosphorus.[41]

Results. The establishment of legally mandated pollution limits in Chesapeake Bay as well as other efforts to reduce nitrogen and phosphorus loadings, have been highly effective. Since 1984, average nitrogen concentrations in the Bay have dropped by 23%, and phosphorus levels have dropped by 8%. In the same time, underwater plants in the Bay have increased their geographic coverage by four-fold.[39] Water quality has improved as well: about 42% of the Bay and its tidal tributaries met clean water standards from 2015 to 2017–the highest percent compliance with clean water standards since 1985.[42,43]

Conclusion. Efforts to reduce the negative effects of nitrogen and phosphorus have been successful, but there is still work to be done for the Chesapeake Bay. To protect the Bay and its underwater plants, continuing efforts to reduce the agricultural and urban sources of nitrogen and phosphorus should be a top priority.

SUPPLEMENTARY APPENDIX TEXT BOX 5. Addressing the Mental Health Consequences of the Deepwater Horizon Oil Spill

Background: After the massive 2010 Deepwater Horizon Oil Spill in the Gulf of Mexico, USA, sharp increases were reported in incidence of post-traumatic stress disorder, depression and substance abuse among disaster response workers and people living in nearby communities.[44-46] The regional mental and behavioral health infrastructure was insufficient to meet the needs of the exposed population.[47]

Solution: Using funds generated in large lawsuit against the companies responsible for the spill, Mental and Behavioral Health Capacity Projects were created in coastal regions of Louisiana, Alabama, Florida, and Mississippi. These projects improved access to mental and behavioral health services in Gulf Coast communities by placing psychologists and psychiatrists in primary care clinics, developing a telepsychiatry network, and building sustainable, long-term capacity in mental and behavioral health. Community engagement was a key pillar of the program. A stepped-care approach ensured that each patient received an appropriate level of care, and "care managers" provided longitudinal support services.[47]

Results: The Mental and Behavioral Health Capacity Projects have now been sustained for nearly a decade, and they have enabled creation of a robust mental healthcare infrastructure in a low-income community that has faced recurrent natural disasters and pollution episodes.[47] In the final quarter of

2019, these programs provided over 12,000 direct services.[48] Quantifiable improvements in mental and behavioral health have resulted.

Conclusions: Ocean pollution has multiple effects on mental and behavioral health, and these effects become especially obvious in the aftermath of acute pollution events such as the Deepwater Horizon Oil Spill. The success of the Mental and Behavioral Health Capacity Projects in the US Gulf Coast region documents the importance of providing mental and behavioral health interventions to communities affected by ocean pollution. This program provides a model for a public-health-based approach to mental and behavioral health care that is rooted in community engagement. It is a framework that can be replicated in addressing future episodes of ocean pollution.

References:

1. Dorman DC, Chiu W, Hales BF, et al. Polybrominated diphenyl ether (PBDE) neurotoxicity: a systematic review and meta-analysis of animal evidence. *Journal of Toxicology and Environmental Health, Part B.* 2018;21(4):269-289.
2. Pessah IN, Lein PJ, Seegal RF, Sagiv SK. Neurotoxicity of polychlorinated biphenyls and related organohalogens. *Acta neuropathologica.* 2019:1-25.
3. Lind PM, Salihovic S, Stubleski J, Kärrman A, Lind L. Association of Exposure to Persistent Organic Pollutants With Mortality Risk: An Analysis of Data From the Prospective Investigation of Vasculature in Uppsala Seniors (PIVUS) Study. *JAMA network open.* 2019;2(4):e193070-e193070.
4. Skakkebaek NE, De Meyts ER, Main K. Testicular dysgenesis syndrome: an increasingly common developmental disorder with environmental aspects. *Apmis.* 2001;109(S103):S22-S30.
5. Reguera B, Alonso R, Moreira Á, Méndez S, Dechraoui Bottein M-Y. Guide for designing and implementing a plan to monitor toxin-producing microalgae. 2016.
6. Clausing RJ, Losen B, Oberhaensli FR, et al. Experimental evidence of dietary ciguatoxin accumulation in an herbivorous coral reef fish. *Aquatic toxicology.* 2018;200:257-265.
7. Dechraoui M-YB, Tiedeken JA, Persad R, et al. Use of two detection methods to discriminate ciguatoxins from brevetoxins: Application to great barracuda from Florida Keys. *Toxicon.* 2005;46(3):261-270.
8. Rossini GP, Hess P. Phycotoxins: chemistry, mechanisms of action and shellfish poisoning. In: *Molecular, clinical and environmental toxicology.* Springer; 2010:65-122.
9. Lawrence J, Loreal H, Toyofuku H, Hess P, Iddya K, Ababouch L. Assessment and management of biotoxin risks in bivalve molluscs. *FAO Fisheries and Aquaculture Technical Paper.* 2011(551):I.
10. Zendong Z, Bertrand S, Herrenknecht C, et al. Passive sampling and high resolution mass spectrometry for chemical profiling of French coastal areas with a focus on marine biotoxins. *Environmental science & technology.* 2016;50(16):8522-8529.
11. Liu D, Keesing JK, Dong Z, et al. Recurrence of the world's largest green-tide in 2009 in Yellow Sea, China: Porphyra yezoensis aquaculture rafts confirmed as nursery for macroalgal blooms. *Marine Pollution Bulletin.* 2010;60(9):1423-1432.

12. Wang M, Hu C, Barnes BB, Mitchum G, Lapointe B, Montoya JP. The great Atlantic Sargassum belt. *Science.* 2019;365(6448):83-87.
13. Lassus P, Chomérat N, Hess P, Nézan E. Toxic and Harmful Microalgae of the World Ocean (Micro-algues toxiques et nuisibles de l'océan mondial) IOC Manuals and Guides 68 (English/French). In: Copenhagen: International Society for the Study of Harmful Algae (ISSHA …; 2016.
14. Piotrowska M, Popowska M. Insight into the mobilome of Aeromonas strains. *Frontiers in microbiology.* 2015;6:494.
15. Dubnau D, Blokesch M. Mechanisms of DNA uptake by naturally competent bacteria. *Annual review of genetics.* 2019;53:217-237.
16. Oberbeckmann S, Labrenz M. Marine Microbial Assemblages on Microplastics: Diversity, Adaptation, and Role in Degradation. *Annual review of marine science.* 2019;12.
17. Labrenz M, Kesy K, Oberbeckmann S, Kreikemeyer B. Spatial environmental heterogeneity determines young biofilm assemblages on microplastics in Baltic Sea mesocosms. *Frontiers in microbiology.* 2019;10:1665.
18. Phillips KE, Satchell KJ. Vibrio vulnificus: from oyster colonist to human pathogen. *PLoS pathogens.* 2017;13(1):e1006053.
19. Weynberg KD, Voolstra CR, Neave MJ, Buerger P, Van Oppen MJ. From cholera to corals: viruses as drivers of virulence in a major coral bacterial pathogen. *Scientific reports.* 2015;5:17889.
20. Dallaire-Dufresne S, Tanaka KH, Trudel MV, Lafaille A, Charette SJ. Virulence, genomic features, and plasticity of Aeromonas salmonicida subsp. salmonicida, the causative agent of fish furunculosis. *Veterinary microbiology.* 2014;169(1-2):1-7.
21. Landrigan PJ, Fuller R, Acosta NJ, et al. The Lancet Commission on pollution and health. *The Lancet.* 2018;391(10119):462-512.
22. Lamb JB, Van De Water JA, Bourne DG, et al. Seagrass ecosystems reduce exposure to bacterial pathogens of humans, fishes, and invertebrates. *Science.* 2017;355(6326):731-733.
23. European Environmental Agency E. Contaminants in Europe's Seas Moving Towards a Clean, Non-Toxic Marine Environment. 2019.
24. Fliedner A, Rüdel H, Jürling H, Müller J, Neugebauer F, Schröter-Kermani C. Levels and trends of industrial chemicals (PCBs, PFCs, PBDEs) in archived herring gull eggs from German coastal regions. *Environmental Sciences Europe.* 2012;24(1):7.
25. Okaichi T. Red tide problems in the Seto Inland sea, Japan. *Red tides: biology, environmental science, and toxicology.* 1989:137-142.
26. Okaichi T, Yanagi T. Sustainable development in the Seto Inland Sea, Japan. *Terra Scientific Publishing Company, Tokyo.* 1997.
27. Bodeanu N. Microalgal blooms in the Romanian area of the Black Sea and contemporary eutrophication conditions. *Toxic phytoplankton blooms in the sea.* 1993:203-209.
28. Bodeanu N, Moncheva S, Ruta G, Popa L. Long-term evolution of the algal blooms in Romanian and Bulgarian Black Sea waters. *Cercetari Marine= Recherches Marines.* 1998.
29. HATS. Harbour Area Treatment Scheme. https://www.dsd.gov.hk/others/HATS2A/en/hats-background/hats-stage-2a/benefits-of-hats-stage-2a.html, 2019.
30. Brownlee I. Hong Kong harbour's return to health should be a green light for more water sports events. 2015.
31. Bowen J, Baillie C, Grabowski J, et al. Boston Harbor, Boston, Massachusetts, USA: Transformation from 'the harbor of shame'to a vibrant coastal resource. *Regional Studies in Marine Science.* 2019;25:100482.
32. MWRA. Boston Harbor Project: An Environmental Success Story. 2014; http://www.mwra.com/01news/2008/bhpenvironentalsuccess/bhpenvsuccess.htm.

33. Jin D, Watson C, Kite-Powell H, Kirshen P. Evaluating Boston Harbor Cleanup: An Ecosystem Valuation Approach. *Frontiers in Marine Science.* 2018;5:478.
34. Levy PF, Connor MS. The Boston Harbor cleanup. *New England Journal of Public Policy.* 1992;8(2):7.
35. D. F. The Reefs of American Samoa: A Story of Hope. 2014; https://ocean.si.edu/ecosystems/coral-reefs/reefs-american-samoa-story-hope.
36. NOAA. Coral reef condition: A status report for American Samoa. 2018; https://www.coris.noaa.gov/monitoring/status_report/docs/AmerSamoa_status_report_forweb.pdf.
37. Craig P, DiDonato G, Fenner D, Hawkins C. The state of coral reef ecosystems of American Samoa. *The state of coral reef ecosystems of the United States and pacific freely associated states.* 2005:312-337.
38. Minogue K. *Chesapeake Bay's Underwater Plants Stage Record-Breaking Comeback, Thanks to Nutrient Diet.* Smithsonian Environmental Research Center. 2018.
39. Lefcheck JS, Orth RJ, Dennison WC, et al. Long-term nutrient reductions lead to the unprecedented recovery of a temperate coastal region. *Proceedings of the National Academy of Sciences.* 2018;115(14):3658-3662.
40. Orth RJ, Moore KA. Chesapeake Bay: an unprecedented decline in submerged aquatic vegetation. *Science.* 1983;222(4619):51-53.
41. EPA. *Chesapeake Bay TMDL Executive Summary.* Chesapeake Bay TMDL2010.
42. Zhang Q, Murphy RR, Tian R, et al. Chesapeake Bay's water quality condition has been recovering: Insights from a multimetric indicator assessment of thirty years of tidal monitoring data. *Science of the Total Environment.* 2018;637:1617-1625.
43. Program CB. Water Quality Standards Attainment and Monitoring. n.d.
44. Osofsky HJ, Osofsky JD, Hansel TC. Deepwater horizon oil spill: mental health effects on residents in heavily affected areas. *Disaster medicine and public health preparedness.* 2011;5(4):280-286.
45. Kwok RK, McGrath JA, Lowe SR, et al. Mental health indicators associated with oil spill response and clean-up: cross-sectional analysis of the GuLF STUDY cohort. *The Lancet Public Health.* 2017;2(12):e560-e567.
46. SAMHSA. *Behavioral Health in the Gulf Coast Region Following the Deepwater Horizon Oil Spill.* 2013.
47. Osofsky HJ, Osofsky JD, Wells JH, Weems C. Integrated care: meeting mental health needs after the Gulf oil spill. *Psychiatric Services.* 2014;65(3):280-283.
48. GRHOP. Mental and Behavioral Health Capacity Project. 2020; http://www.grhop.org/MentalBehavioral.html.
