# Supplementary material for: Human Health and Ocean Pollution
Source: Ann Glob Health. 2020 Dec 3;86(1):151. doi: 10.5334/aogh.2831 (PMC7731724; doi:10.5334/aogh.2831)
Supplement: Declaration of Monaco. — This Declaration summarizes the key findings and conclusions of the Monaco Commission on Human Health and Ocean Pollution. It is based on the recognition that all life on Earth depends on the health of the seas. It presents a Call to Action – an urgent message addressed to leaders in all countries and to all citizens of Earth urging us to safeguard human health and preserve our Common Home by acting now to end pollution of the ocean. [file agh-86-1-2831-s2.pdf]

December 3, 2020

Declaration of Monaco:

Advancing Human Health & Well-Being by Preventing Ocean Pollution

On 2-3 December 2020, the Prince Albert II de Monaco Fondation, the Centre Scientifique de Monaco and Boston College convened the *Monaco International Symposium on Human Health & the Ocean in a Changing World* in partnership with the Government of the Principality of Monaco, the World Health Organization (WHO), UN Environment (UNEP), the International Agency for Atomic Energy (IAEA) , the Monaco Oceanographic Institute, the French National Centre for Scientific Research (CNRS), the Mediterranean Science Commission (CIESM), the European Marine Board, the Woods Hole Oceanographic Institution, and the Scripps Institution of Oceanography.

Symposium participants presented comprehensive, up-to-date information on all forms of ocean pollution and their effects on human health. They examined trends and geographic patterns of ocean pollution and pollution-related disease. They proposed recommendations for the prevention and control of ocean pollution and the improvement of human health and well-being.

Major Conclusions of the Symposium are these:

1. **Pollution of the oceans is widespread, worsening, and in many places poorly controlled. Human activity that releases unwanted wastes into the sea is the major source.**
   - Ocean pollution is a complex mixture of plastic waste, toxic metals, manufactured chemicals, oil spills, urban and industrial wastes, pesticides, fertilizers, pharmaceutical waste, agricultural runoff and sewage.
   - More than 80% arises from land-based sources.
   - Chemical and plastic pollutants have become ubiquitous in the earth's oceans. They contaminate seas and marine organisms from the high Artic to the abyssal depths.

2. **Ocean pollution has multiple negative impacts on human health and well-being. The magnitude, severity and geographic ranges of these effects are increasing**.
   - Petrochemicals and persistent organic pollutants (POPs) in the oceans threaten the marine microorganisms that produce much of the earth's oxygen supply.
   - Mercury pollution of the oceans causes high levels of contamination in tuna and other widely eaten fish. When pregnant mothers eat mercury-contaminated fish, mercury enters their bodies and can damage their children's developing brains. The consequences are lifelong reductions in intelligence (IQ), developmental delays, and increased risk of attention deficit/hyperactivity disorder (ADHD).
   - Coal combustion in power plants and factories is the main source of marine mercury pollution. Gold mining is a second source.

- In adults, mercury pollution increases risk of cardiovascular disease and accelerates cognitive decline, thus increasing risk of dementia.
- Plastic microparticles and microfibers – the microscopic breakdown products of plastic pollution – persist in the oceans for years, enter the marine food web and concentrate in fish and shellfish consumed by humans
- Plastic microparticles carry multiple toxic chemicals– PCBs, phthalates, bisphenol A, brominated flame retardants, organophosphorus compounds, organotin compounds, and perfluorinated chemicals. When they enter the human body in plastic microparticles, these chemicals can reduce male fertility, increase risk of heart disease, disrupt endocrine signaling, depress immune function, and cause cancer.
- Agricultural runoff. Industrial waste and human sewage released into harbors and coastal waters trigger Harmful Algal Blooms (HABs), increase incidence of ciguatera fish poisoning and toxic shellfish poisoning, build antibiotic resistance, and accelerate the spread of life-threatening infections.

3. **Ocean pollution has multiple harmful effects on marine ecosystems. Climate change and ocean acidification exacerbate these effects.**
   - Plastic pollution kills seabirds, fish and marine mammals.
   - Pharmaceutical waste, chemical pollution and sewage discharges damage fragile estuaries and mangrove swamps that are the nurseries of the sea.
   - Chemical pollutants and pharmaceutical wastes destroy coral reefs.
   - Increased absorption of carbon dioxide into the oceans – the direct consequence of fossil fuel combustion - results in ocean acidification. Ocean acidification destroys coral reefs, dissolves oysters, and dissolves calcium-containing plankton at the base of the marine food web.
   - Pollution contributes to declines in fish stocks and threatens food security of millions.

4. **Ocean pollution is deeply unjust.**
   - Ocean pollution and all its impacts fall disproportionately on people in small island nations, indigenous communities in the far North, coastal communities in the Global South, and fishing communities worldwide - populations that create only miniscule amounts of pollution.
   - This is environmental injustice on a global scale.

5. **Ocean pollution is not well mapped.**
   - Current knowledge of ocean pollution and its impacts on human health is incomplete.
   - Information on the geographic distribution and concentrations of pollutants in the oceans and on the sizes of the human populations exposed to ocean pollution is fragmentary and confined mostly to the seas that border high-income countries.
   - Conference participants note that this lack of complete information provides no excuse for delaying action to control ocean pollution

6. **<u>The Good News</u>. Ocean pollution can be prevented and controlled.**
   - Like all forms of pollution, ocean pollution can be prevented and controlled.
   - The key first step is to identify and control the land-based sources that account for 80% of ocean pollution

- Targeted, data-driven strategies based on law, policy, and technology and backed by strong enforcement are essential to achieve control.
- These strategies are highly effective and have achieved significant successes against ocean pollution.
- Polluted harbors have been cleaned, estuaries rejuvenated, and coral reefs restored.
- Interventions against ocean pollution are highly cost-effective. They have boosted economies, increased tourism, and restored fisheries. These benefits will last for centuries
- Prevention and control of ocean pollution have improved human health, prevented disease and extended longevity.

World leaders and global citizens who recognize the gravity of ocean pollution, acknowledge its growing dangers, engage civil society and the global public, and take bold, evidence-based action to stop pollution at source will be critical to preventing ocean pollution and safeguarding human health.

A Call for Action to End Ocean Pollution and Protect Human Health and Well-Being

Acting on the above Conclusions, the participants in the *Monaco International Symposium on Human Health & the Ocean in a Changing World* call upon leaders in all countries to safeguard human health and to preserve the beautiful, but fragile planet that is our Common Home. We urge the following actions:

- **Transition Rapidly from Fossil Fuels to Renewable Energy Sources – Wind, Solar and Tidal Power**

- **Prevent Mercury Pollution of the Oceans** by eliminating coal combustion and controlling all industrial uses of mercury, especially in gold mining.

- **End Plastic Pollution of the Oceans by Reducing Plastic Production and Imposing a Global Ban on Production of Single-Use Plastic.**

- **Promote Effective Waste Management and Plastic Recycling**

- **Reduce Releases of Nitrogen, Phosphorus, Animal Waste, Industrial Discharges and Human Sewage into Coastal Waters.**

- **Support Robust Monitoring of Ocean Pollution.**

- **Extend Regional and International Marine Pollution Control Programs to all Countries.**

- **Support Research Programs that Increase Knowledge of the Extent, Severity and Human Health Impacts of Ocean Pollution.**

- **Create and Safeguard Marine Protected Areas.**
